# National, subnational and risk attributed burden of chronic respiratory diseases in Iran from 1990 to 2019

**DOI:** 10.1186/s12931-023-02353-1

**Published:** 2023-03-11

**Authors:** Mahsa Heidari-Foroozan, Alisam Aryan, Zahra Esfahani, Mohammad Amin Shahrbaf, Sahar Saeedi Moghaddam, Mohammad Keykhaei, Erfan Ghasemi, Mohammad-Mahdi Rashidi, Nazila Rezaei, Seyyed-Hadi Ghamari, Mohsen Abbasi-Kangevari, Sahar Mohammadi Fateh, Yousef Farzi, Negar Rezaei, Bagher Larijani

**Affiliations:** 1 Non-Communicable Diseases Research Center, Endocrinology and Metabolism Population Sciences Institute, Tehran University of Medical Sciences, No. 10, Al-E-Ahmad and Chamran Highway Intersectionsection, Tehran, 1411713137 Iran; 2 Student Research Committee, School of Medicine, Shahid Beheshti University of Medical Sciences, Tehran, Iran; 3 Faculty of Medicine, Shahid Beheshti University of Medical Sciences, Tehran, Iran; 4 Kiel Institute for the World Economy, Kiel, Germany; 5 Division of Cardiology, Department of Medicine, The Johns Hopkins University School of Medicine, Baltimore, MD USA; 6 Endocrinology and Metabolism Research Center, Endocrinology and Metabolism Clinical Sciences Institute, Tehran University of Medical Sciences, Tehran, Iran

**Keywords:** Asthma, Attributable risk factor, Chronic respiratory disease, COPD, DALYs, Decomposition, Global burden of disease, Iran, Pneumoconiosis

## Abstract

**Introduction:**

Data on the distribution of the burden of diseases is vital for policymakers for the appropriate allocation of resources. In this study, we report the geographical and time trends of chronic respiratory diseases (CRDs) in Iran from 1990 to 2019 based on the Global burden of the Disease (GBD) study 2019.

**Methods:**

Data were extracted from the GBD 2019 study to report the burden of CRDs through disability-adjusted life years (DALYs), mortality, incidence, prevalence, Years of Life lost (YLL), and Years Lost to Disability (YLD). Moreover, we reported the burden attributed to the risk factors with evidence of causation at national and subnational levels. We also performed a decomposition analysis to determine the roots of incidence changes. All data were measured as counts and age-standardized rates (ASR) divided by sex and age group.

**Results:**

In 2019, the ASR of deaths, incidence, prevalence, and DALYs attributed to CRDs in Iran were 26.9 (23.2 to 29.1), 932.1 (799.7 to 1091.5), 5155.4 (4567.2 to 5859.6) and 587,911 (521,418 to 661,392) respectively. All burden measures were higher in males than females, but in older age groups, CRDs were more incident in females than males. While all crude numbers increased, all ASRs except for YLDs decreased over the studied period. Population growth was the main contributor to the changes in incidence at a national and subnational levels. The ASR of mortality in the province (Kerman) with the highest death rate (58.54 (29.42 to 68.73) was four times more than the province (Tehran) with the lowest death rate (14.52 (11.94 to 17.64)). The risk factors which imposed the most DALYs were smoking (216 (189.9 to 240.8)), ambient particulate matter pollution (117.9 (88.1 to 149.4)), and high body mass index (BMI) (57 (36.3 to 81.8)). Smoking was also the main risk factor in all provinces.

**Conclusion:**

Despite the overall decrease in ASR of burden measures, the crude counts are rising. Moreover, the ASIR of all CRDs except asthma is increasing. This suggests that the overall incidence of CRDs will continue to grow in the future, which calls for immediate action to reduce exposure to the known risk factors. Therefore, expanded national plans by policymakers are essential to prevent the economic and human burden of CRDs.

**Supplementary Information:**

The online version contains supplementary material available at 10.1186/s12931-023-02353-1.

## Introduction

Chronic respiratory diseases (CRDs) is a general term that includes a range of diseases that affect the airways and the other structures of the lungs [1]. Common CRD types include chronic obstructive pulmonary disease (COPD), asthma, pneumoconiosis, interstitial lung diseases, and pulmonary sarcoidosis [2]. CRDs are a leading concern worldwide; based on the Global Burden of Disease (GBD) study, the number of deaths due to CRDs has increased to approximately 3.7 million deaths in 2019. They were also cited as the third cause of death, only behind cardiovascular diseases and cancers, accounting for 7% of all mortality globally. Furthermore, with a 39.8% increase, the prevalence of CRDs has risen to 544·9 million in 2019 [3].

Although CRDs are among the most prominent contributors to the burden of non-communicable diseases (NCDs) and low-cost interventions can prevent or treat these diseases, they have received less attention from researchers and policymakers than other NCDs [4]. In contrast, clinicians and epidemiologists have reported the scarcity of data on the dispersion of CRDs, which makes implementing cost-effective preventive plans challenging [5]. Nationwide preventive programs are essential for reaching Sustainable Development Goal (SDG) 3.4, which was set by the United Nations in 2015 and states that the number of deaths caused by NCDs, such as CRDs, is expected to be cut to one-third by 2030 [6]. Like other countries, Iran is committed to reaching the SDG 3.4 by 2030, and precise epidemiological data of the burden of diseases such as CRDs aids in tracking the progress toward SDG 3.4. Moreover, it assists the governors and policymakers in the implementation of national plans to control the burden of diseases and have a better prediction of a disease economic burden in the future. Furthermore, by having an accurate estimation of the contribution of risk factors to the uprising burden of diseases, preventive measures can be taken [7].

As one of the most prominent groups of NCDs in Iran, CRDs are responsible for a non-negligible proportion of the disease burden. Based on Varmaghani et al*.* study, the pooled incidence of asthma in Iran was 7.95% in 2016 (5.85% to 10.06%), which is higher than in numerous countries such as Pakistan, Oman, and India [8]. Moreover, based on a study conducted in the north of Iran, it was reported that 5% of the population suffer from COPD [9].

Considering the burden of CRDs in Iran, it is crucial for policymakers to develop suitable action plans to control its humane and economic burden. This goal can be persuaded by having a precise knowledge of the condition of CRDs in the country., thus our study provides a comprehensive knowledge of the national and subnational Burden of CRDs in Iran based on the GBD findings from 1990 to 2019 by reporting crude counts and age-standardized (ASR) of burden measures disability-adjusted life years (DALYs), mortality, incidence, and prevalence, Years of Life lost (YLL), and Years Lost to Disability (YLD) and the burden attributed to its risk factors which are vital for resource allocation and policy-making. To the best of our knowledge, there has been no up to date estimate of the burden of CRDs in Iran [8].

## Methods

### Overview

GBD 2019 estimates the burden of 369 diseases and injuries in seven super regions, 21 regions, and 204 countries and territories from 1990 to 2019 in terms of incidence, prevalence mortality, YLL, YLD, and DALYs. It also reports the burden of disease attributed to 87 behavioral, environmental, metabolic, and occupational risk factors. Details on the data components, data gathering, resources, analytics, and population health metrics for the GBD 2019 have been discussed in detail elsewhere [10, 11]. All burden measures are presented as count and age-standardized based on the GBD reference population to remove the effect of age structure [12]. All counts and rates are reported with a 95% uncertainty interval taking into account the potential errors in measurement, modelling and possible biases. Decomposition analysis was applied between 1990 and 2019 to identify the effect of age structure, population growth, and incidence rate changes on the observed incidents cases [13]. This study is in accordance with the Guidelines for Accurate and Transparent Health Estimates Reporting (GATHER).

### Burden estimation framework

According to the GBD dictionary CRDs include the following five categories: asthma, COPD, interstitial lung disease and pulmonary sarcoidosis, pneumoconiosis (including silicosis, asbestosis, coal worker pneumoconiosis, and other pneumoconioses), and other chronic respiratory diseases. International Classification of Diseases (ICD)-10 was mapped to define CRDs; mortality and non-fatality (Additional file 1) [10, 14]. We obtained our data from the GBD results tool, https://ghdx.healthdata.org/gbd-results-tool.

### Risk factor estimation framework

GBD provides a comprehensive estimation of the burden attributed to 87 risk factors in 204 countries, via the comparative risk assessment (CRA); it’s a systematic and comparable approach to risk factor quantification that offers a valuable tool for synthesizing evidence on risks and risk-outcomes associations. GBD CRA updates each GBD round, integrating new data on risk-outcome pairs, risk exposure levels, and risk-outcome associations. There are critical steps to CRA: inclusion of risk-outcome causal connections in the analysis; estimation of relative risk as a function of exposure; estimation of exposure levels and distributions; determination of the counterfactual level of exposure, the level of exposure with minimum risk called the theoretical minimum risk exposure level (TMREL); decomposition of population attributable fractions and attributable burden; and estimation of mediation of different risk factors [11]. Since 2010, the risk-outcome pairs have been bound to fulfill the World Cancer Research Fund criteria in order to prove their causal relationship [11]. The identified risk factors for CRDs were ambient ozone pollution, ambient particulate matter pollution, high body mass index, high temperature, household air pollution from solid fuels, low temperature, occupational asthmagens, occupational exposure to asbestos, occupational exposure to silica, occupational particulate matter, gases, and fumes, second hand smoke and smoking. To calculate the burden of each risk factor, the level of exposure to each risk factor known as population attributable fraction, was multiplied by the whole burden of CRD by age, sex, location and year [11].

### Socio-demographic index (SDI)

SDI is a composite indicator that is calculated from the geometric mean of three measures, including average years of educational attainment in individuals older than 15 years, income per capita, and total fertility rate in individuals younger than 25 years in a country which is expressed on the scale of zero to one with one correlating with a lower rate of fertility, higher income and educational level [10] All of the provinces were classified based on SDI into high SDI, high-middle, middle, low-middle and low SDI. The SDI strongly correlates with variables such as mortality, life expectancy, and DALY thus acts as a tool to predict health outcomes and compare regional health outcomes [15].

### Burden measures

Counts and rates of incidence, mortality, and DALY are reported as the primary measurements of CRD's burden. Data from the death registry, disease registry, and scientific literature were the input for the original estimations of mortality by GBD [9]. Incidence was calculated by dividing deaths by death-to-incidence ratio. Prevalence estimations were provided using Dis-Mod-MR version 2.1, which is a Bayesian regression tool used by the GBD study. We calculated ten year prevalence and then multiplied the prevalence of each period by its disability weight to estimate the YLD. We used the death number by age and normative worldwide age expectancy to calculate YLL. And at last, DALYs were calculated by adding of YLDs and YLLs to each other.

### Statistical analysis

We applied a universal age structure from 2019 to calculate Age-standardized rates (ASR). We reported the ASR of each burden measure per 100,000 population using a population distribution with the age composition of the GBD reference population [12]. Uncertainty intervals (UIs) were defined as the 2.5th, and 97.5th percentiles of the uncertainty distribution by randomly selecting 1000 draws from the posterior distribution Differences between point estimates were reported as significant if more than 95% of values for the difference were either positive or negative.

### Decomposition analysis

In order to perform decomposition analysis, two scenarios were calculated in order to determine the proportion of changes in incidence which is attributed to population growth, age structure, and age-specific incidence rate in the country; at first the number of mentioned elements in 1990 was applied to the whole population of 2019. The difference between the obtained number and the number of cases in 1990 was associated with population growth. In the following scenario, the age-specific rates in 1990, were applied to the population size, age structure and sex structure of 2019. The gap between the second scenario and the number of cases in 2019 was associated to age-specific rates, and the difference of these two scenarios was associated to population aging.

### Software

R software version 4.0.5 was used to perform Data visualization and calculation.

## Results

### Incidence and prevalence

The number of people living with CRDs has increased 48.9% (38.2 to 61.1) and reached 4,076,810 (3,634,959 to 4,620,503) in 2019 (Table 1). 65.09% of which was due to asthma, 37.93% due to COPD, 0.70% due to Interstitial lung disease, pulmonary sarcoidosis, and due to 0.14% pneumoconiosis. It is of note that counts and age-standardized prevalence rate (ASPR) of all CRD types increased, except for asthma (− 22.6% (− 26.7 to − 18.0)) (Additional file 5: Table S1).

**Table 1:** Burden measures at national level, 1990 vs 2019

Measure	Metric	Both			Female			Male		
		1990	2019	% Change (1990 to 2019)	1990	2019	% Change (1990 to 2019)	1990	2019	% Change (1990 to 2019)
Incidence	All ages number	545,475 (438,127 to 678,389)	731,231 (633,931 to 845,833)	34.1 (22.2 to 48.7)*	255,144 (208,070 to 314,014)	355,020 (309,913 to 411,176)	39.1 (26.4 to 54.2)*	290,332 (230,056 to 365,901)	376,211 (324,274 to 438,955)	29.6 (18.4 to 43.9)*
	Age-standardized rate (per 100,000)	948.8 (820.2 to 1108.5)	932.1 (799.7 to 1091.5)	− 1.8 (− 4.8 to 1.1)	924.3 (805 to 1073.9)	915.4 (797.9 to 1063.5)	− 1 (− 4 to 2)	972.4 (829.6 to 1133.8)	948 (805 to 1112.1)	− 2.5 (− 5.9 to 0.8)
Prevalence	All ages number	2,737,620 (2,317,641 to 3,270,976)	4,076,810 (3,634,959 to 4,620,503)	48.9 (38.2 to 61.1)*	1,283,937 (1,093,692 to 1,514,276)	1,926,747 (1,716,073 to 2,197,384)	50.1 (39.7 to 62)*	1,453,683 (1,220,040 to 1,760,773)	2,150,063 (1,912,497 to 2,434,525)	47.9 (36.5 to 60.8)*
	Age-standardized rate (per 100,000)	5670.3 (5033.6 to 6394.6)	5155.4 (4567.2 to 5859.6)	− 9.1 (− 12.1 to − 6.1)*	5519.2 (4881.7 to 6239.1)	4908.4 (4353.4 to 5582.1)	− 11.1 (− 14.4 to − 8.1)*	5818.2 (5150.7 to 6548.9)	5400.5 (4774.5 to 6134)	− 7.2 (− 10.5 to − 3.7)*
Deaths	All ages number	8207 (7169 to 9874)	16,835 (14,588 to 18,193)	105.1 (67.2 to 137.4)*	3362 (2545 to 4272)	6764 (5356 to 7883)	101.2 (54.6 to 202)*	4845 (4272 to 6048)	10,071 (8775 to 10,865)	107.9 (60.7 to 140.2)*
	Age-standardized rate (per 100,000)	42.6 (36.1 to 52.6)	26.9 (23.2 to 29.1)	− 36.8 (− 49 to − 26.6)*	36.2 (27.2 to 47.8)	22.4 (17.5 to 26.1)	− 38.1 (− 55.1 to − 10.1)*	49.4 (42.9 to 62.7)	31.4 (27.6 to 34)	− 36.4 (− 50.7 to − 27.3)*
DALYs	All ages number	365,429 (315,783 to 425,585)	587,911 (521,418 to 661,392)	60.9 (42.1 to 77)*	159,388 (129,921 to 190,367)	252,949 (219,784 to 289,416)	58.7 (37.3 to 102.7)*	206,041 (178,519 to 241,938)	334,963 (296,527 to 374,789)	62.6 (38.4 to 81.1)*
	Age-standardized rate (per 100,000)	1113.2 (983.7 to 1275.4)	794.1 (705.2 to 886.5)	− 28.7 (− 38.1 to − 21.2)*	979.1 (792.3 to 1180.9)	690.4 (600.6 to 784.4)	− 29.5 (− 40.4 to − 9.6)*	1241.6 (1100.8 to 1472.1)	898.6 (801.8 to 997.7)	− 27.6 (− 39.9 to − 20.1)*
YLLs	All ages number	220,281 (194,885 to 259,953)	321,132 (283,709 to 347,041)	45.8 (18.9 to 69.8)*	89,918 (64,689 to 111,292)	124,668 (103,502 to 147,168)	38.6 (7 to 121.8)*	130,363 (115,386 to 161,137)	196,464 (174,269 to 210,647)	50.7 (17.2 to 75.3)*
	Age-standardized rate (per 100,000)	786.2 (687.2 to 946.8)	456 (405.2 to 492.8)	− 42 (− 52.6 to − 32.9)*	655.8 (496 to 828.4)	362.5 (295.3 to 426.8)	− 44.7 (− 56.8 to − 17)*	911.4 (804.5 to 1136.2)	550.1 (492.9 to 589.9)	− 39.6 (− 52.9 to − 30.5)*
YLDs	All ages number	145,147 (105,334 to 194,582)	266,779 (206,944 to 330,855)	83.8 (65.4 to 106.4)*	69,469 (50,894 to 92,923)	128,280 (100,303 to 159,560)	84.7 (66.5 to 105.6)*	75,678 (54,579 to 102,579)	138,499 (106,122 to 174,294)	83 (63.7 to 107.4)*
	Age-standardized rate (per 100,000)	327 (247.1 to 419.3)	338.1 (262.7 to 420.2)	3.4 (− 1.4 to 8.7)	323.3 (248.1 to 410.7)	327.9 (255.1 to 408.5)	1.4 (− 3.7 to 6.7)	330.3 (246.9 to 424.5)	348.5 (267.8 to 437.6)	5.5 (0.3 to 12)*

Data in parentheses are 95% Uncertainty Intervals (95% UIs)

DALYs = Disability-Adjusted Life Years; YLLs = Years of Life Lost; YLDs = Years Lived with Disability

*Significant change from 1990 to 2019

**Fig. 1:**
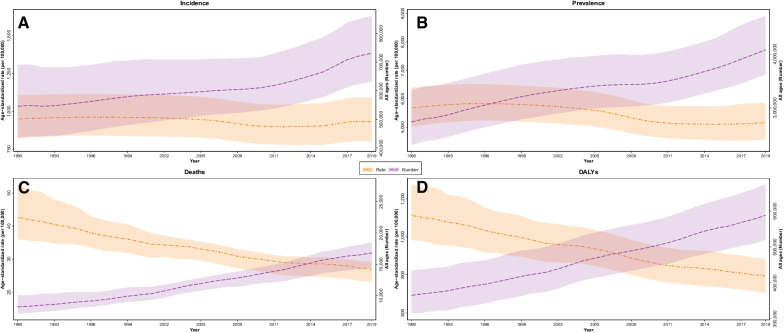
Time trend of age-standardized rate and all ages number at national level, 1990 to 2019 **A **Incidence **B **Prevalence **C **Deaths **D **DALYs

In 2019, there were 731,231 (633,931 to 845,833) incident cases of CRD with an age-standardized incidence rate (ASIR) of 932.1 (799.7 to 1091.5) per 100,000 people, and it was more common in males (948 (805 to 1112.1)) than females (915.4 (797.9 to 1063.5)). While the number of incident cases increased by 34.1 (22.2 to 48.7), the ASIR decreased fell slightly compared to 1990 (948.8 (820.2 to 1108.5) (Table 1, Fig. 1A).

In 2019, south Khorasan was the province with the highest ASIR (1017.8 (884.3 to 1185.5)), while Hormozgan possessed the lowest ASIR (858.7 (728.5 to 1019.2)). In almost all provinces, ASR of CRDs incidence displayed a falling pattern until 2015; however, it acquired a rising pattern after 2015 (Additional file 2: Fig. S2). Moreover, in all provinces except North Khorasan, the incidence rate was slightly higher in males compared to females (Additional file 6: Table S2).

**Fig. 2:**
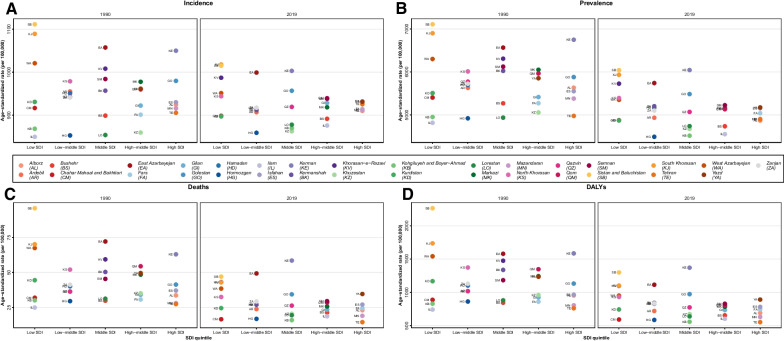
The rate of burden measures by SDI quantiles and province. 1990 vs 2019 **A** Incidence **B** Prevalence **C** Deaths **D** DALYs

Like prevalence, the highest incident CRD was asthma with an ASIR of 3280.1 (2717.3 to 3978.4). However, the second most common occurring CRD was interstitial lung disease and pulmonary sarcoidosis (245 (198.5 to 298.1)), which is followed by COPD (103,602 (94,771 to 113,265)) (Additional file 5: Table S1).

With regards to SDI quantiles in 2019 in high and high-middle SDI quantiles, the ASIRs were relatively similar within each quantile. However, the gap between ASIRs of provinces of all quantiles became narrower during the studies period, while the ASR of incidence in provinces of middle, low-middle, and low SDI quantiles was more scattered. Generally, the highest ASIRs belonged to low SDI provinces such as South Khorasan (1017.8 (884.3 to 1185.5)) and Sistan and Baluchistan (Sistan and Baluchistan, 1015.1 (883.1 to 1176.3)) (Fig. 2A). However, Sistan and Baluchistan had the most prominent percentage of decrease during the studies period (− 8.7 (− 13.6 to − 4) followed by South Khorasan (6.6 (− 11.4 to − 1.6)).

**Fig. 3:**
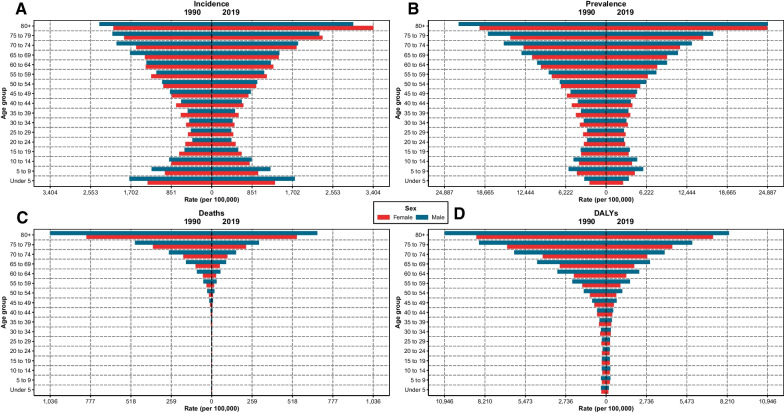
The rate of burden measures by sex and age group at national level. 1990 vs 2019 **A** Incidence **B** Prevalence **C** Deaths **D** DALYs

Concerning age groups, the highest incidence rate belonged to the + 70 age group with an ASIR of 2407.9 (2072.2 to 2784.9) per 100,000. Moreover, the incidence of CRDs had another peak in the under five age group with an ASIR of 1546.7 (929.5 to 2416.1) but decreased until the age 30, and after that, it started rising again. It is of note that in younger age groups the CRDs occur more in males, while it is more common in females in older age groups (Fig. 3A). All of provinces also displayed the national trend for incidence (Additional file 3: Fig. S3).

Decomposition analysis revealed that the incidence of CRDS has increased 34.1% during the period between 1990 to 2019; population growth was responsible for 44%, expected new cases were responsible for − 5.7%, and at last incidence rate change accounted for − 4.2% of the observed difference. Nearly all provinces displayed a similar pattern. However, in Ardebil (− 4.4%) and Hamadan (− 4.5%) the overall incidence had decreased compared to 1990, and in Gilan, the age structure had a minimal positive effect (0.2%), and in Lorestan (1.6%), Tehran (1.3%), Ilam 0.7% and the incidence rate contributed positively to the incidence change (Table 2).

**Table 2:** Decomposition analysis of the change in incidence number at national and subnational levels, 1990 vs 2019

Location		Sex	New cases		Expected new cases in 2019		% 1990–2019 new cases change cause			% 1990–2019 new cases overall change (%)
			1990	2019	Population growth	Population growth + aging	Population growth (%)	Age structure change (%)	Incidence rate change (%)	
Iran (Islamic Republic of)		Both	545,475	731,231	785,486	754,242	44	− 5.7	− 4.2	34.1
		Female	255,144	355,020	369,824	366,126	44.9	− 1.4	− 4.4	39.1
		Male	290,332	376,211	415,449	387,904	43.1	− 9.5	− 4	29.6
Subnational	Alborz	Both	13,665	24,684	26,702	25,720	95.4	− 7.2	− 7.6	80.6
		Female	6305	11,873	12,549	12,531	99	− 0.3	− 10.4	88.3
		Male	7360	12,811	14,134	13,222	92	− 12.4	− 5.6	74.1
	Ardebil	Both	11,480	10,979	12,729	11,655	10.9	− 9.4	− 5.9	− 4.4
		Female	5360	5361	5946	5729	10.9	− 4	− 6.9	0
		Male	6120	5618	6783	5899	10.8	− 14.4	− 4.6	− 8.2
	Bushehr	Both	6388	10,041	10,953	10,324	71.4	− 9.8	− 4.4	57.2
		Female	3055	4789	5032	4942	64.7	− 3	− 5	56.7
		Male	3333	5253	5932	5351	78	− 17.4	− 2.9	57.6
	Chahar Mahaal and Bakhtiari	Both	6829	8459	9243	8710	35.3	− 7.8	− 3.7	23.9
		Female	3158	4097	4293	4178	36	− 3.7	− 2.5	29.8
		Male	3671	4362	4947	4519	34.8	− 11.7	− 4.3	18.8
	East Azarbayejan	Both	35,671	38,713	42,151	41,494	18.2	− 1.8	− 7.8	8.5
		Female	17,061	18,938	20,154	20,445	18.1	1.7	− 8.8	11
		Male	18,611	19,774	21,998	20,978	18.2	− 5.5	− 6.5	6.3
	Fars	Both	33,084	42,539	44,972	42,520	35.9	− 7.4	0.1	28.6
		Female	15,586	20,750	21,277	20,796	36.5	− 3.1	− 0.3	33.1
		Male	17,499	21,789	23,688	21,718	35.4	− 11.3	0.4	24.5
	Gilan	Both	21,235	23,666	23,694	23,743	11.6	0.2	− 0.4	11.4
		Female	10,022	11,582	11,196	11,560	11.7	3.6	0.2	15.6
		Male	11,214	12,084	12,497	12,196	11.4	− 2.7	− 1	7.8
	Golestan	Both	13,356	17,390	19,224	18,173	43.9	− 7.9	− 5.9	30.2
		Female	6326	8547	9129	8959	44.3	− 2.7	− 6.5	35.1
		Male	7031	8843	10,092	9196	43.5	− 12.7	− 5	25.8
	Hamadan	Both	16,145	15,418	16,700	16,007	3.4	− 4.3	− 3.7	− 4.5
		Female	7372	7412	7751	7674	5.1	− 1.1	− 3.6	0.5
		Male	8773	8006	8934	8310	1.8	− 7.1	− 3.5	− 8.7
	Hormozgan	Both	8163	15,063	16,734	15,204	105	− 18.7	− 1.7	84.5
		Female	3864	7168	7926	7462	105.1	− 12	− 7.6	85.5
		Male	4300	7895	8809	7748	104.9	− 24.7	3.4	83.6
	Ilam	Both	3922	4658	5177	4631	32	− 13.9	0.7	18.8
		Female	1822	2285	2435	2282	33.7	− 8.4	0.2	25.4
		Male	2100	2373	2739	2348	30.4	− 18.6	1.2	13
	Isfahan	Both	34,842	46,284	47,761	46,930	37.1	− 2.4	− 1.9	32.8
		Female	16,143	22,455	22,554	22,533	39.7	− 0.1	− 0.5	39.1
		Male	18,699	23,829	25,172	24,486	34.6	− 3.7	− 3.5	27.4
	Kerman	Both	19,479	30,913	34,729	33,003	78.3	− 8.9	− 10.7	58.7
		Female	9052	14,647	15,930	15,545	76	− 4.2	− 9.9	61.8
		Male	10,427	16,266	18,823	17,423	80.5	− 13.4	− 11.1	56
	Kermanshah	Both	16,046	17,075	18,669	18,181	16.3	− 3	− 6.9	6.4
		Female	7405	8382	8794	8895	18.7	1.4	− 6.9	13.2
		Male	8641	8694	9859	9265	14.1	− 6.9	− 6.6	0.6
	Khorasan-e-Razavi	Both	47,835	62,526	67,630	64,840	41.4	− 5.8	− 4.8	30.7
		Female	22,727	30,480	32,196	32,040	41.7	− 0.7	− 6.9	34.1
		Male	25,108	32,045	35,429	32,729	41.1	− 10.8	− 2.7	27.6
	Khuzestan	Both	28,030	39,767	42,663	40,117	52.2	− 9.1	− 1.2	41.9
		Female	12,797	18,856	19,605	19,120	53.2	− 3.8	− 2.1	47.3
		Male	15,233	20,911	23,041	20,995	51.3	− 13.4	− 0.6	37.3
	Kohgiluyeh and Boyer-Ahmad	Both	4418	5938	6712	6006	51.9	− 16	− 1.5	34.4
		Female	2058	2839	3124	2895	51.9	− 11.1	− 2.7	38
		Male	2360	3099	3588	3120	52	− 19.8	− 0.9	31.3
	Kurdistan	Both	11,892	13,903	15,915	14,603	33.8	− 11	− 5.9	16.9
		Female	5667	6772	7605	7183	34.2	− 7.4	− 7.3	19.5
		Male	6225	7131	8309	7392	33.5	− 14.7	− 4.2	14.6
	Lorestan	Both	13,515	14,663	15,450	14,441	14.3	− 7.5	1.6	8.5
		Female	6308	7227	7287	7121	15.5	− 2.6	1.7	14.6
		Male	7207	7437	8156	7311	13.2	− 11.7	1.7	3.2
	Markazi	Both	11,647	12,827	13,884	13,757	19.2	− 1.1	− 8	10.1
		Female	5424	6232	6430	6602	18.5	3.2	− 6.8	14.9
		Male	6223	6595	7459	7131	19.9	− 5.3	− 8.6	6
	Mazandaran	Both	23,693	29,993	31,150	30,599	31.5	− 2.3	− 2.6	26.6
		Female	11,279	14,923	14,809	15,076	31.3	2.4	− 1.4	32.3
		Male	12,414	15,070	16,342	15,497	31.6	− 6.8	− 3.4	21.4
	North Khorasan	Both	6201	7795	8526	8198	37.5	− 5.3	− 6.5	25.7
		Female	2975	3846	4095	4059	37.6	− 1.2	− 7.2	29.3
		Male	3226	3949	4431	4137	37.3	− 9.1	− 5.8	22.4
	Qazvin	Both	8930	11,267	12,490	11,767	39.9	− 8.1	− 5.6	26.2
		Female	4226	5496	5934	5814	40.4	− 2.8	− 7.5	30.1
		Male	4705	5771	6555	5944	39.3	− 13	− 3.7	22.7
	Qom	Both	6881	11,819	12,665	12,304	84.1	− 5.3	− 7.1	71.8
		Female	3229	5720	6000	5995	85.8	− 0.2	− 8.5	77.2
		Male	3652	6099	6661	6318	82.4	− 9.4	− 6	67
	Semnan	Both	4687	6494	7261	6937	54.9	− 6.9	− 9.4	38.6
		Female	2181	3210	3426	3366	57.1	− 2.8	− 7.2	47.2
		Male	2505	3284	3830	3565	52.9	− 10.6	− 11.2	31.1
	Sistan and Baluchistan	Both	17,114	28,359	34,562	32,162	101.9	− 14	− 22.2	65.7
		Female	7828	13,418	15,815	15,150	102	− 8.5	− 22.1	71.4
		Male	9287	14,942	18,746	17,043	101.9	− 18.3	− 22.6	60.9
	South Khorasan	Both	7730	8195	9604	8864	24.2	− 9.6	− 8.6	6
		Female	3444	3924	4274	4157	24.1	− 3.4	− 6.8	13.9
		Male	4286	4272	5330	4687	24.4	− 15	− 9.7	− 0.3
	Tehran	Both	74,204	121,555	121,568	120,569	63.8	− 1.3	1.3	63.8
		Female	34,442	59,460	57,629	58,210	67.3	1.7	3.6	72.6
		Male	39,762	62,094	63,829	62,538	60.5	− 3.2	− 1.1	56.2
	West Azarbayejan	Both	23,475	30,645	34,424	33,373	46.6	− 4.5	− 11.6	30.5
		Female	11,098	14,845	16,288	16,212	46.8	− 0.7	− 12.3	33.8
		Male	12,377	15,799	18,135	17,108	46.5	− 8.3	− 10.6	27.7
	Yazd	Both	6532	10,234	11,049	10,678	69.2	− 5.7	− 6.8	56.7
		Female	2999	4859	5106	5005	70.3	− 3.4	− 4.9	62
		Male	3533	5375	5940	5707	68.1	− 6.6	− 9.4	52.1
	Zanjan	Both	8385	9366	10,192	9727	21.6	− 5.5	− 4.3	11.7
		Female	3933	4626	4804	4748	22.1	− 1.4	− 3.1	17.6
		Male	4452	4741	5387	4962	21	− 9.5	− 5	6.5

### Mortality

The number of CRD-attributed deaths more than doubled and raised to 16,835 (14,588 to 18,193) deaths in 2019. Meanwhile, the age-standardized death rate (ASDR) declined to 26.9 (23.2 to 29.1) deaths in 100,000 people with a -36.4% (− 50.7 to − 27.3) change.

ASDR was significantly higher in males (31.4 (27.6 to 34)) compared to females (22.4 (17.5 to 26.1)) in 2019, and concordantly, the change increments were more in females (− 38.1 (− 55.1 to − 10.1)) in comparison to males (− 36.4 (− 50.7 to − 27.3)) (Table 1, Fig. 1C).

Similar to the national trend, all provinces showed a decreasing ASDR. Furthermore, in 2019, the province with the highest ASDR was Kerman, with 58.5 (29.4 to 68.7)) deaths per 100,000 people, while Tehran possessed the lowest ASDR (14.5 (11.9 to 17.6)), which decreased − 47.1% (− 62.2 to − 26.4) compared to 1990. Moreover, deaths attributed to CRDs were higher in males of all provinces in comparison to females (Additional file 6: Table S2).

The CRD causing the most deaths in 2019 and 1990 was COPD, with an ASDR of 20.3 (17.7 to 22.1) per 100,000 people. The death rates due to all CRDs remained relatively stable with negligible increases, except for asthma which its ASDR experienced a − 69.7% (− 78.9 to − 59.7) fall and decreased to 5.6 (4.8 to 6.2) (Additional file 5: Table S1).

In 2019, In all SDI quantiles, ASRs of mortality attributed to CRD lessened compared to 1990, and provinces within each quantile had relative values with some exceptions. Kerman (58.54 (29.42 to 68.73)), as a middle-SDI, showed a higher ASDR than other provinces in the same quantile; also, east Azarbayejan (49.3 (34.2 to 57.8)) had a higher ASDR than other low-middle SDI provinces (Fig. 2C, Additional file 6: Table S2). Regarding the changes in mortality rate, Sistan and Baluchistan (− 50.9% (− 62.7 to − 33.8) and Chahar mahaal and Bakhtiari (− 48.1% (− 61.5 to − 32.8) showed the highest change increments.

Regarding the age groups, unlike incidence that had a peak in younger age groups, death rates were insignificant until age 40. After that, the death rates increased and peaked in the + 70 age group (329.9 (279.8 to 359)). Moreover, the ASDRs are reduced in all age groups compared to 1990. It is noteworthy that in all age groups, deaths attributed to CRDs are more in males, while in the older age groups, CRDs were more common in females (Fig. 3). Interestingly, the same pattern was detected at the subnational level in all provinces (Additional file 1: Fig. S1).

### DALY, YLL, and YLD

In 2019, CRDs were responsible for 587,911 (521,418 to 661,392) DALYs, which is significantly higher than the associated DALYs in 1990 (365,429 (315,783 to 425,585)). In contrast, the ASR of DALYs was substantially lower in 2019 (794.1 (705.2 to 886.5)) compared to 1990 (1113.2 (983.7 to 1275.4)) (Fig. 1D).

Furthermore, it was significantly higher in males (334,963 (296,527 to 374,789) than females (252,949 (219,784 to 289,416)) at a national level. A similar pattern to DALYs was observed for both sexes combined and separated in YLLs and YLDs except for a subliminal increase in YLDs (both sexes: 3.4% − 1.4 to 8.7; females: 1.4 (− 3.7 to 6.7); males: 5.5 (0.3 to 12)) (Table 1).

All provinces displayed a downward trend for DALYs, with Kerman and Tehran having the highest (1371 (885.8 to 1571.6)) and lowest (553.2 (470.1 to 644.2)) DALYs, respectively. YLLs also exhibited a similar pattern to national level. On the contrary, when studying YLDs, provinces such as Alborz (8.3 (− 13.3 to − 2.8), Kermanshah (− 3.3 (− 8.6 to 2.7)), Markazi (− 2.3 (− 8.4 to 4.8)), Qazvin (− 1.5 (− 7.3 to 4.6)), Qom (− 5.2 (− 10.5 to 0.4)), Sistan and Baluchistan (− 3.2 (− 8.7 to 3.4)), Semnan (− 2.8 (− 8.7 to 3.6)) and west Azarbayejan (3.8 (− 9.7 to 2.6)) had a opposite trend to national trend, and their ASR YLDs decreased. Furthermore, males suffered from non-negligibly more DALYs than females in all provinces (Additional file 6: Table S2).

The contribution of YLLS to DALYs was slightly more in comparison to YLDs in both 2019 and 1990 (Table 1). The same trend was observed in all provinces in both years, except Tehran, with a higher YLD than YLL in 2019. (Additional file 4: Fig. S4).

The CRD that resulted in the highest DALY was COPD with an ASR of 517.2 (471 to 560.8), followed by asthma (232.3 (185 to 299.3)), other chronic respiratory diseases (26.8 (19.6 to 32.5), Interstitial lung disease and pulmonary sarcoidosis (14.7 (10.3 to 17.7)) and Pneumoconiosis 3.1 (2.6 to 3.7). YLDs also exhibited the same order, but in the context of YLLS, Interstitial lung disease and pulmonary sarcoidosis (11.2 (6.9 to 13.4)) accounted for more YLLs than other chronic respiratory diseases (9.6 (3.9 to 13.2) (Additional file 5: Table S1).

The number of DALYs increased gradually with the increasing age groups until the 50–69 age group (189,923 (168,499 to 209,733), then declined briefly to 185,770 (165,389 to 200,540) in the + 70 age group. Meanwhile, the DALYs rate was the highest in the + 70 age group with 5350.6 (4763.6 to 5776) per 100,000 people. It is of note that in almost all age groups, males suffered from more DALYs than females in the national landscape and also in most of the age groups in all provinces (Fig. 3D).

Regarding the SDI quantiles, the DALY of provinces in all SDI quantiles decreased compared to 1990. The highest DALYs belonged to middle (Kerman (1371(885.8 to 1571.6)) and low (Sistan and Baluchistan (1300.1 (923.2 to 1513.1)) and low-middle SDI (East Azarbayejan (1114.5 (894.4 to 1264.6) provinces, while the lowest DALY was observed in the high SDI province Tehran (553.2 (470.1 to 644.2)). Furthermore, the most noticeable decreases were detected in the low SDI quantile countries such as Sistan and Baluchistan (− 42.6 (− 53.9 to − 21.6)). (Fig. 2, Additional file 6: Table S2).

### Risk factors

At the national level, the number of all DALYs attributed to the risk factors experienced a 114.5% (84.9 to 137.9) change and reached 310,187 (274,601 to 344,635) years in 2019. In contrast, the rate of DALYs declined over the period by − 21.9% (− 33.1 to − 13) and fell to 423.3 (376.3 to 468.4). The major part of DALYs attributed to risk factors is formed by males (561.5 (496.6 to 616)) rather than females (285 (240.5 to 332.7)) (Table 3).

**Table 3:** Burden measures attributed to all risk factors combined at national level, 1990 vs 2019

Measure	Metric	Year						% Change (1990 to 2019)		
		1990			2019					
		Both	Female	Male	Both	Female	Male	Both	Female	Male
Deaths	All ages number	4365 (3739 to 5260)	1386 (978 to 1862)	2979 (2542 to 3775)	10,555 (9350 to 11,549)	3413 (2719 to 4033)	7142 (6175 to 7821)	141.8 (98.1 to 177.9)	146.2 (88.2 to 278.1)	139.8 (85.2 to 181.8)
	Age-standardized rate (per 100,000)	22.7 (19.3 to 27.6)	15.3 (10.8 to 21.3)	30.6 (26 to 38.5)	16.8 (14.7 to 18.5)	11.3 (8.9 to 13.3)	22.3 (19.4 to 24.5)	− 25.9 (− 38.9 to − 14.8)	− 26.3 (− 45 to 9.8)	− 27.4 (− 43.8 to − 15.4)
DALYs	All ages number	144,592 (125,314 to 169,105)	47,705 (36,138 to 60,179)	96,887 (83,223 to 115,636)	310,187 (274,601 to 344,635)	103,632 (87,166 to 121,847)	206,554 (182,367 to 228,277)	114.5 (84.9 to 137.9)	117.2 (84.3 to 190.9)	113.2 (74.9 to 141.4)
	Age-standardized rate (per 100,000)	541.8 (470.2 to 633.9)	367.9 (277.7 to 471.1)	711.7 (614.1 to 850.8)	423.3 (376.3 to 468.4)	285 (240.5 to 332.7)	561.5 (496.6 to 616)	− 21.9 (− 33.1 to − 13)	− 22.5 (− 36.1 to 3.1)	− 21.1 (− 35.4 to − 10.8)
YLLs	All ages number	104,434 (89,891 to 125,428)	32,291 (22,161 to 42,088)	72,144 (61,682 to 90,621)	197,382 (175,426 to 216,329)	60,998 (50,337 to 72,482)	136,384 (119,476 to 148,785)	89 (55.3 to 117.6)	88.9 (51.4 to 198.2)	89 (45 to 123.1)
	Age-standardized rate (per 100,000)	414.2 (354 to 497.4)	269.5 (189.4 to 358.9)	555.8 (475.1 to 699.7)	281 (250 to 306.8)	178 (144.5 to 210.8)	384 (336 to 419.1)	− 32.2 (− 44.3 to − 22.1)	− 34 (− 48.4 to 1.6)	− 30.9 (− 46.6 to − 19.3)
YLDs	All ages number	40,158 (30,719 to 50,865)	15,415 (11,515 to 20,168)	24,743 (18,704 to 31,158)	112,804 (88,430 to 138,240)	42,635 (32,420 to 53,602)	70,170 (54,863 to 86,346)	180.9 (163.7 to 200.4)	176.6 (153.6 to 200.3)	183.6 (165.8 to 206.5)
	Age-standardized rate (per 100,000)	127.6 (99 to 157)	98.4 (74.6 to 125.4)	156 (119.2 to 194.3)	142.3 (111.9 to 172.3)	107.1 (82.4 to 133.3)	177.4 (140.1 to 216.4)	11.5 (5.2 to 18)	8.9 (0.3 to 17.2)	13.8 (7.4 to 21)

Data in parentheses are 95% Uncertainty Intervals (95% UIs)

*DALYs* Disability-Adjusted Life Years; *YLLs* Years of Life Lost; *YLDs* Years Lived with Disability

**Fig. 4:**
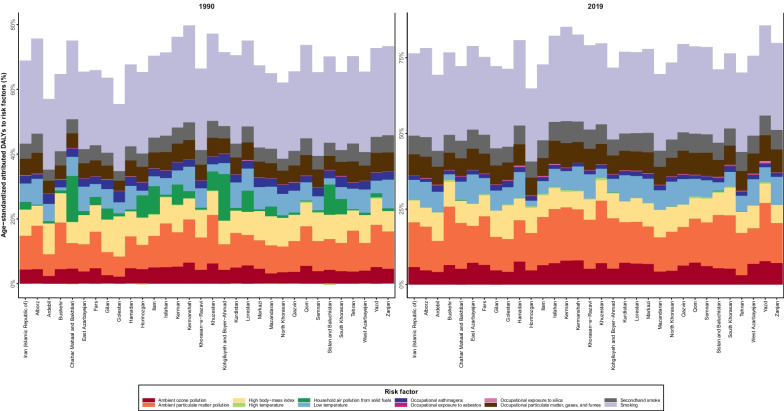
The proportion of DALYs of each province attributed to each risk factor. 1990 vs 2019

In 2019, the risk factor with the most associated rate of DALYs was smoking (216 (189.9 to 240.8)), followed by Ambient particulate matter pollution (117.9 (88.1 to 149.4)), high body mass index (BMI) (57 (36.3 to 81.8)) and Occupational particulate matter, gases, and fumes (54 (44 to 64.7)). The rate of DALYs attributed to high temperature, ambient particulate matter pollution, and occupational exposure to silica and asbestos grew over the studied period, whereas the other eight risk factors had a declining pattern. All risk factors except high BMI, occupational asbestos exposure, and household air pollution from solid fumes imposed more DALYs on males (Additional file 7: Table S3). In a subnational landscape, smoking was similarly the leading risk factor. The percentage of DALYs attributed to household air pollution from fuels decreased over the studied period in all provinces, but for other risk factors was nearly the same (Fig. 4).

## Discussion

This study provides a comprehensive assessment of the burden of CRDs, including their prevalence, incidence, mortality, YLLS, and YLDs from 1990 to 2019 in Iran and its provinces. While in 1990, about 2,700,000 million people were suffering from CRDs, which gave rise to approximately 360,000 thousand DALYs and 8000 deaths, in 2019, these measures raised to 4,100,000 million, 587,900 thousand, and 16,800 thousand, respectively. While the crude counts of burden measures increased during the studied period, all ASRs except for YLDs decreased during the two decades. The same pattern was also observed at a global level for prevalence, incidence, mortality, and DALYs attributed to CRDs [3, 16]. Both changes in the ASRs and the decomposition analysis confirm that the crude numbers' changes were mainly caused by population growth.

This study showed that the incidence, prevalence, deaths, and DALY rates among males were consistently higher than in females, matching the worldwide pattern [3]. Several environmental and physiological reasons can be counted for this finding. menopause is positively associated with more alveolar loss and declined lung function, which explains the higher incidence of CRDs in higher age groups in females than males [17] and based on a meta-analysis by Gan et al*.,* differences in physiology between men and women make women more susceptible to lung function decline when adjusted for the number of cigarettes smoked [18].

Also, due to differences in job distribution between the sexes, males experience more exposure to occupational pollutants [3]. This phenomenon can be partly attributed to males being in more contact with the risk factors in comparison to females. For instance, the prevalence of smoking which is the primary risk factor for CRDs is eightfold more in males compared to females [19]. Another assumption posits that dissimilar metabolism of tobacco components in men and women results in prolonged exposure of women to toxic substances [20]. With the increasing prevalence of smoking among women, a higher burden for CRDs is imagined for females in the future. Furthermore,

The findings of this study indicate that at a subnational level, South Khorasan and Kerman provinces had the highest ASIR and ASR mortality attributed to CRDs. This is in accordance with Varmaghani et al. study, which showed a higher rate of CRDs in southern regions of Iran. There is a higher rate of cigarrete and hookah smoking in the mentioned areas [8, 21]. Moreover, several studies have highlighted the importance of occupational exposure in these provinces. Kerman is a highly industrial and mining province, and because of that, exposure to harmful occupational factors is relatively high [22, 23].

While the overall incidence and prevalence of CRDs saw a minimal decrease, the ASIR and ASPR of all CRDs but asthma had increased compared to 1990; but despite its downward trend, asthma remained the most incident and prevalent CRD in Iran, followed by COPD. However, at the global level, the most common CRD was COPD, which accounted for 55·1% of all CRDs, and in contrast to Iran, the ASPR of all CRDs experienced a fall [3]. Furthermore, the study of Xie et al*.* revealed a decreasing pattern for ASIR of COPD and pneumoconiosis in addition to asthma, which was not the same pattern we found for Iran [16]. The opposite trend of COPD's ASIR in Iran might be partly due to the increased exposure to risk factors such as smoking, air pollution, and occupational situations [24].

However, when considering ASR of deaths and DALYs, COPD also showed a downward trend; in the case of deaths and DALYs, asthma was the second leading disease, preceded by COPD. Moreover, COPD also gave rise to more deaths and DALYs in the global landscape. The major contributor to COPD DALYs in Iran was YLL, while overall in North Africa and the Middle East, the contribution of YLLs and YLDs was almost equal [3]. The decreased ASDR but higher YLLs suggest that although the quality of care for COPD in Iran has improved, more attention needs to be paid to its situation. However, our findings were not in line with Varmaghani et al. 2015 study reported a rising trend for COPD incidence rate; the observed difference might be due to the different time frames and data sources [25].

Various studies have shown that several risk factors affect the incidence of CRDs, such as smoking, air pollution, and high blood pressure [26]. Based on comprehensive research conducted on the GBD database, we report the burden of 12 environmental, metabolic, occupational, and behavioral risk factors [11].

Globally smoking is the leading risk factor for chronic non-communicable diseases such as CRD [27]. It is also the primary risk factor for CRDs at the national level. The previous studies showed that the trend of smoking prevalence did not change significantly during 2004–2016 [28], but at the beginning of this period, the trend of CRDs prevalence was decreasing, and after the change-point year of 2010, it started increasing. Thus, change in the prevalence of other risk factors can be considered as the reason behind the trend of the age-standardized burden measures of CRDs during the 1990–2019 study period. Moreover, smoking results in secondhand smoke, which is another risk factor for CRDs. In a study conducted by Korsbæk et al*.* it was concluded that individuals who experienced exposure to secondhand smoke in adulthood had an Odds Ratio (OR) of 1.49 (1.09–2.05) and 1.25 (0.90–1.74) for developing COPD and asthma respectively [29].

Another group of risk factors for CRDs is pollution, which is divided into air pollution and occupational exposure to pollution. The second leading risk factor was ambient air pollution. Air pollution gives rise to an inflammatory situation that limits the lungs' function [30]. Moreover, various studies have demonstrated that with the progress of global warming, the detrimental effects of air pollution on the respiratory system's health are becoming worse because the concentration of pollutants increases and also, hot temperature, which itself is a risk factor for CRDs, acts in synergy with air pollution to exacerbate health conditions [26]. Between the occupational risks, occupational particulate matter and exposure to asthmagens caused the most DALYs. The other two occupational risk factors which have a minor contribution to CRD DALYs are exposure to silica and asbestos. These particles limit the airflow in the lungs and increase the risk of CRDs, in particular COPD [31]. Our findings were consistent with the results of the De Matteis et al*.* study, which confirmed that the COPD rate was higher in gardners, sculptors and warehouse workers [32]. However, on the contrary, in the study by Ratanachina et al*.* it was reported that occupational exposure did not affect lung function, however, it gave rise to respiratory symptoms such as cough and wheezing [33].

Most of the risk factors for CRD are preventable, and by applying some underused but straightforward strategies, CRDs burden attributed to risk factors can be reduced. The burden of smoking can be reduced by implementing some national constraints and nationwide programs. By stating public bans, the number of smokers and also people exposed to secondhand smoke can be reduced. Moreover, increased tax on tobacco products might lower their target population by increasing the expense of smoking. And at last, a complete ban on their promotion and advertising might reduce the number of smokers, too [34, 35].

Detrimental effects of ambient air pollution can be lowered by regulating daily activity according to the air quality index (AQI), which yields necessary health policies [30]. There is still uncertainty over using personal protective equipment (N95 mask or equivalent) during haze settings, and to date, there are no recommended evidence-based arguments for masks in preventing the effects of air pollution. Novel approaches to mitigating CRDs burdens, such as dietary recommendations and antioxidant supplements, still need to be backed by more robust randomized studies, as previous investigations have brought contrary results [36].

Government and elite policymakers should implement novel initiatives and make use of previous successful stratagem to reduce the burden of CRDs; Moreover, CRDs are closely associated with worse prognosis of COVID-19; and considering the fluctuations in incidence of COVID-19 and its uprisings in different countries and its massive health and economic burden; it is of pivotal importance to control the rate of CRDs which can be achieved[37]by determining the gaps in healthcare services to which can be identified by such epidemiological studies [38].

### Strengths and limitations

This study had its strengths and limitations. One of the strengths of GBD 2019 was the implementation of improved health information coded using the ICD system. GBD 2019 also prevents compositional bias of national estimates by adjusting variance and weighting [39].

One of the limitations of this study which was specific to CRDs, is the controversies over clear case definitions, which often happen as underdiagnoses, particularly in patients at an early stage of the disease, and even over-diagnosis in some groups [40]. Also, since our data is from the GBD study, we could not consider the coexistence of several CRDs simultaneously. Furthermore, due to the existing lag in data acquisition, the data for recent years are projected from the previous years' trends; therefore, more precise data improves the quality of the estimations.

## Conclusions

While the ASR of burden measures of CRD, except YLD has decreased over the studied period, the crude rates of all burden measures have had an upward trend. This phenomenon is explained by the changes in age structure and population aging. The increase in ASIR of almost all CRD types except asthma calls for attention by policymakers to control the rising burden of CRDs. The primary risk factors for CRDs are smoking and ambient particulate matter pollution. Nationwide initiatives and bans can prevent the population's exposure to these two risk factors. By reducing the population's exposure to these risk factors, the burden of CRDs attributed to them is expected to diminish.

## Supplementary Information

**Additional file 1: Figure S1.** Burden rate by subtype at national level.

**Additional file 2: Figure S2.** Burden rate by province during 1990-2019.

**Additional file 3: Figure S3.** The rate of burden measures by sex and age group at subnational level. 1990 vs 2019.

**Additional file 4: Figure S4.** Comparison of YLDs and DALYs by province.

**Additional file 5: Table S1.** Burden measures of CRDs for all ages number and ASR with percentage change by sex at national level, 1990 vs 2019. Data in parentheses are 95% Uncertainty Intervals (95% UIs); DALYs= Disability-Adjusted Life Years; YLLs= Years of Life Lost; YLDs= Years Lived with Disability

**Additional file 6: Table S2.** Burden measures of CRDs for all ages number and ASR with percentage change by sex in all provinces, 1990 vs 2019. Data in parentheses are 95% Uncertainty Intervals (95% UIs); DALYs= Disability-Adjusted Life Years; YLLs= Years of Life Lost; YLDs= Years Lived with Disability

**Additional file 7: Table S3.** Attributed burden measures of CRDs to all risk factors for all ages number and ASR with percentage change by sex at national level, 1990 vs 2019. Data in parentheses are 95% Uncertainty Intervals (95% UIs); DALYs= Disability-Adjusted Life Years; YLLs= Years of Life Lost; YLDs= Years Lived with Disability

## Data Availability

The dataset generated and/or analyzed during the current study is available in the GBD results tool, [https://ghdx.healthdata.org/gbd-results-tool].
