# Supplementary figures and images for: National, subnational and risk attributed burden of chronic respiratory diseases in Iran from 1990 to 2019
Source: Respir Res. 2023 Mar 11;24:74. doi: 10.1186/s12931-023-02353-1 (PMC10006557; doi:10.1186/s12931-023-02353-1)

Incidence

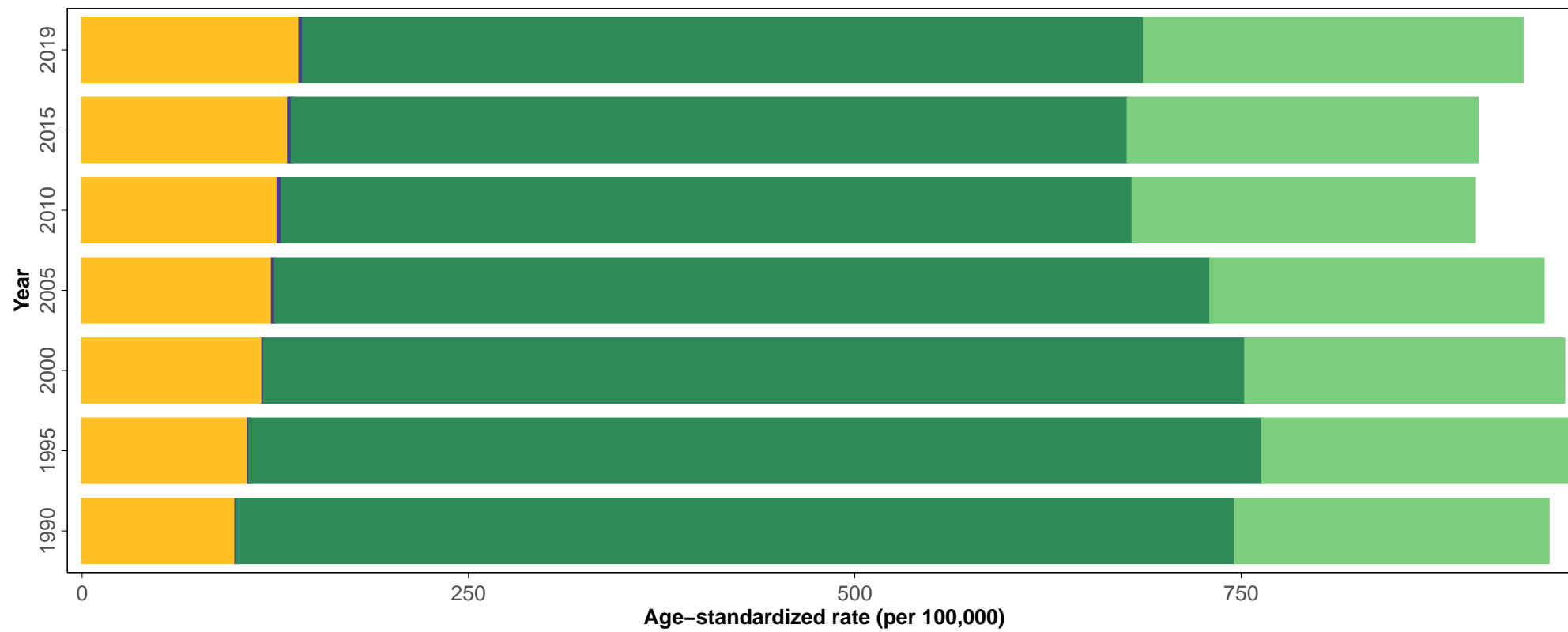

Prevalence

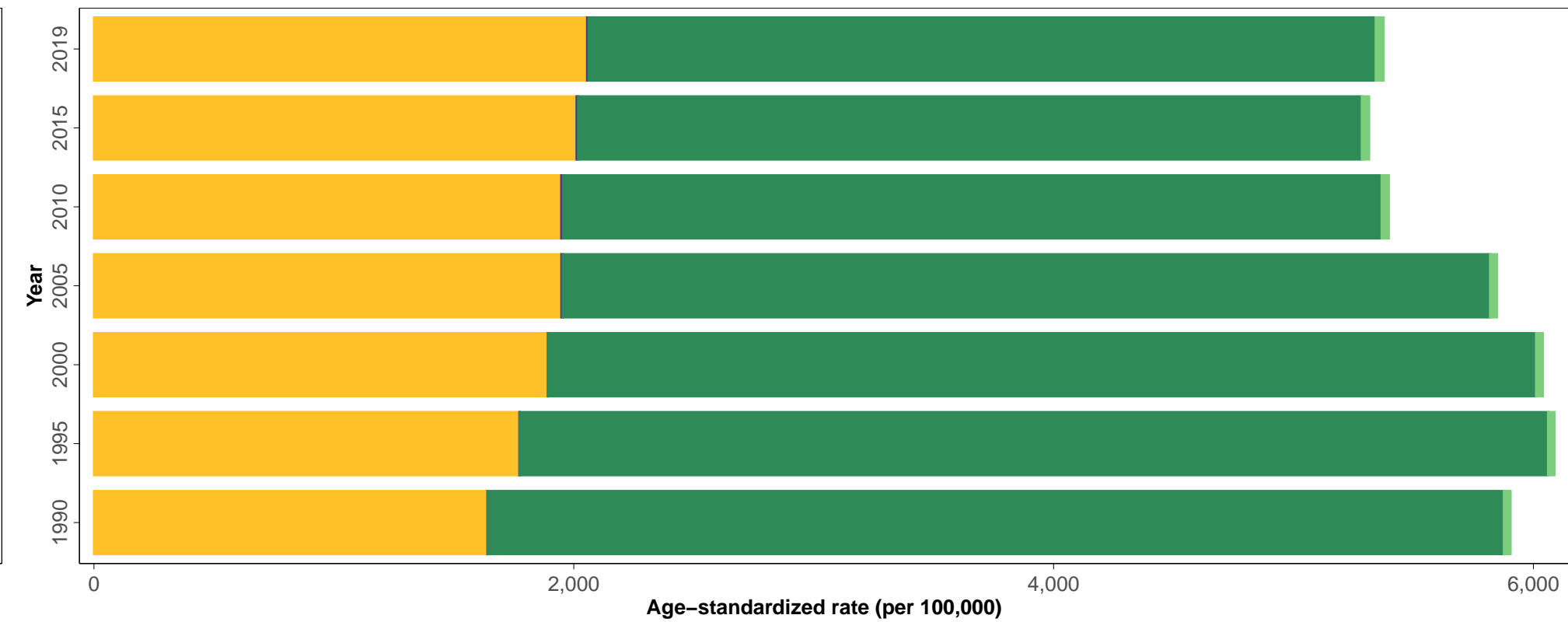

Deaths

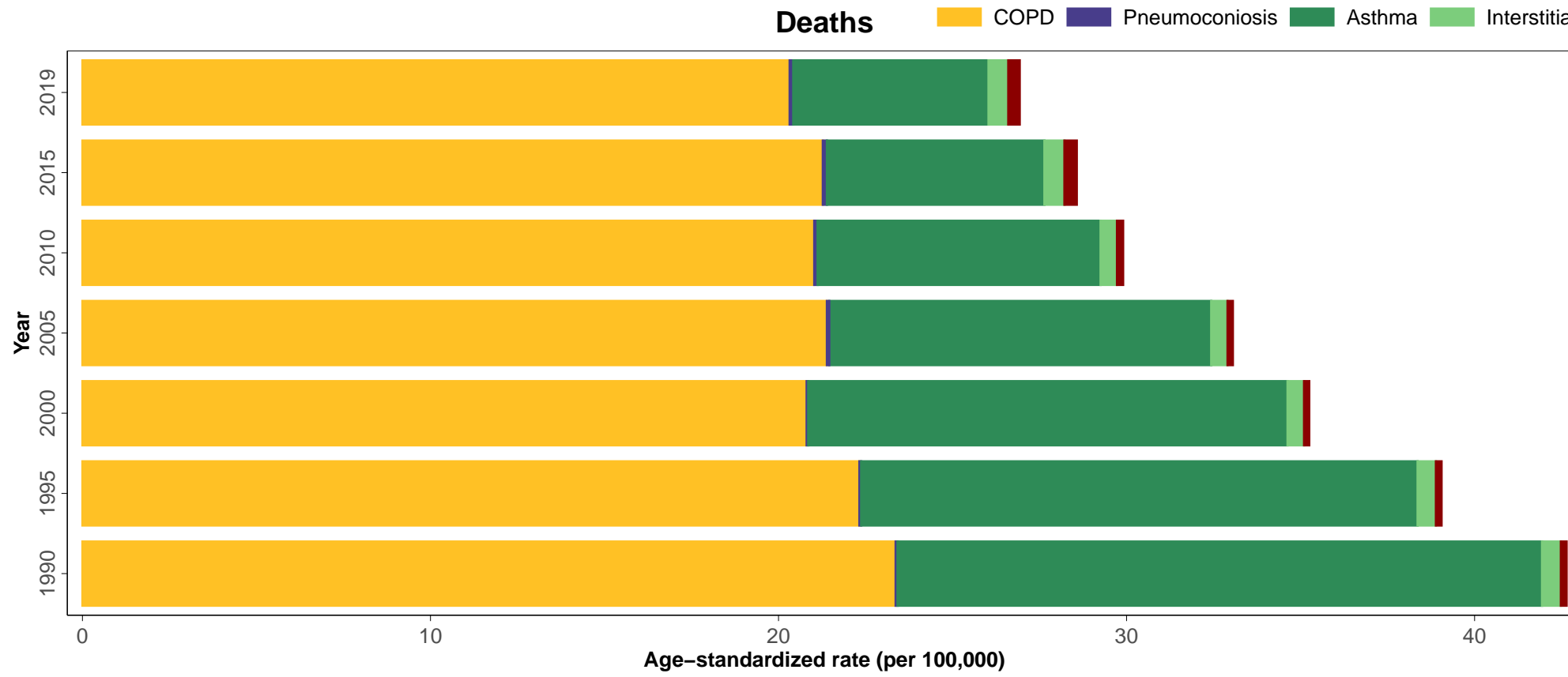

DALYs

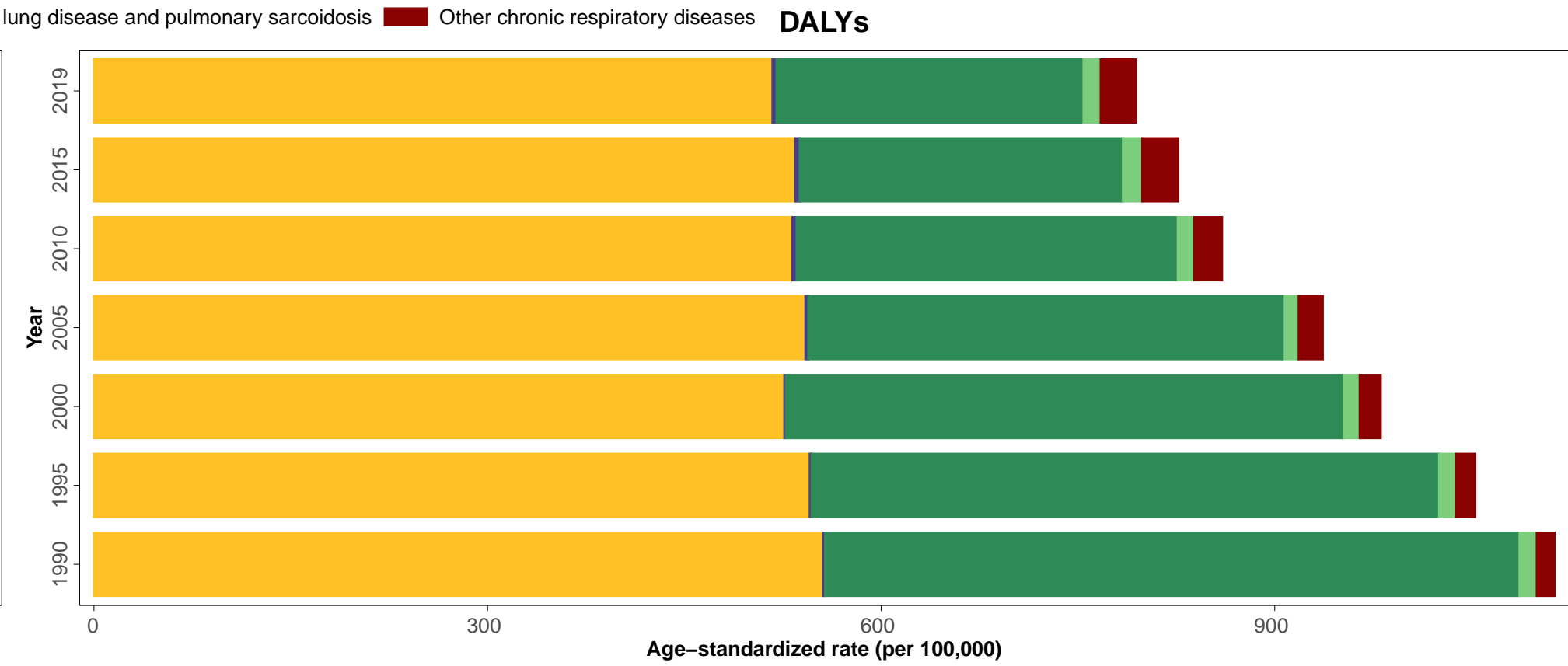

Supplement: Supplementary file 1 — Additional file 1: Figure S1. Burden rate by subtype at national level. [file 12931_2023_2353_MOESM1_ESM.pdf]

Incidence

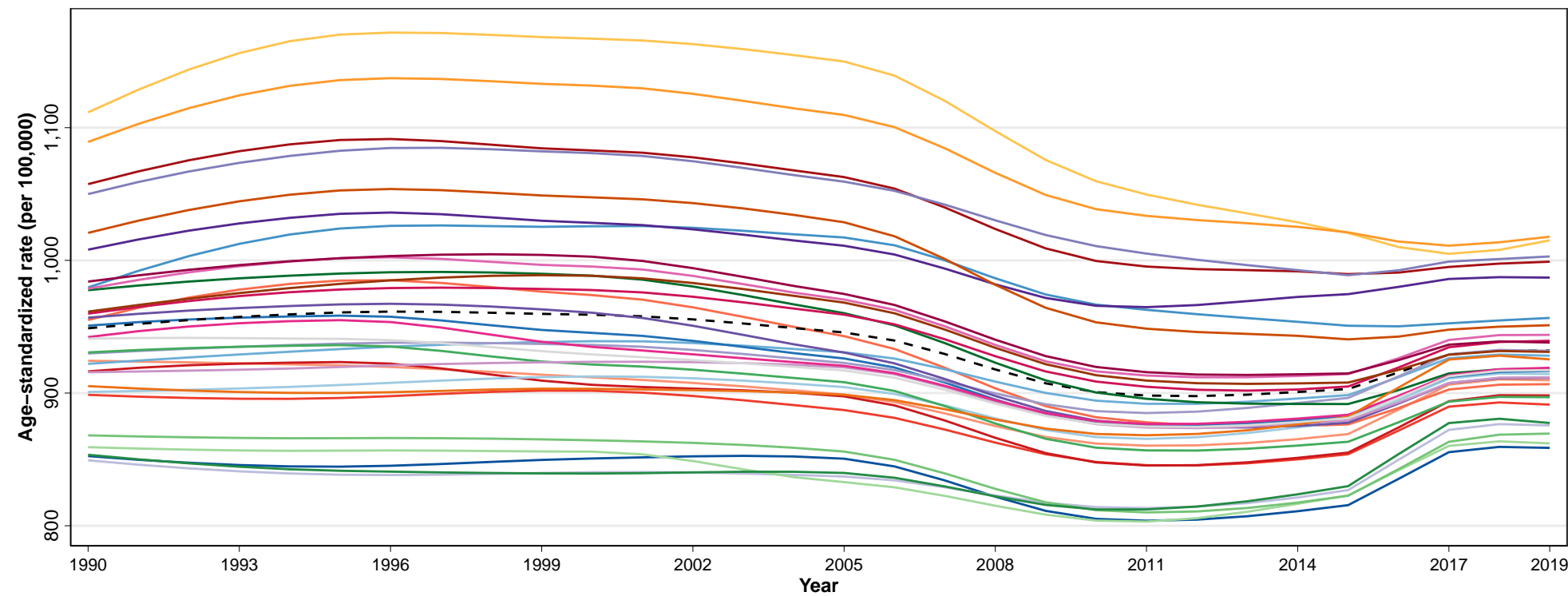

Prevalence

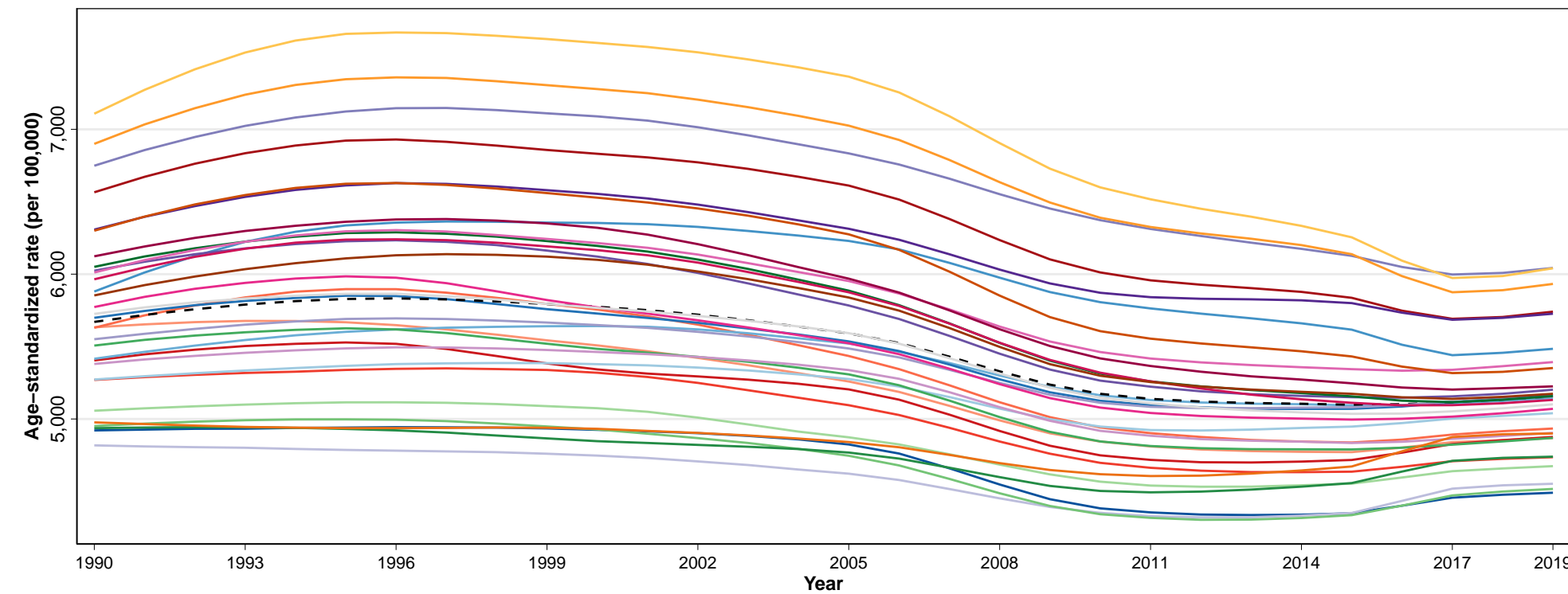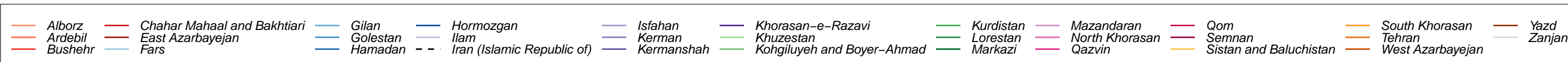

Deaths

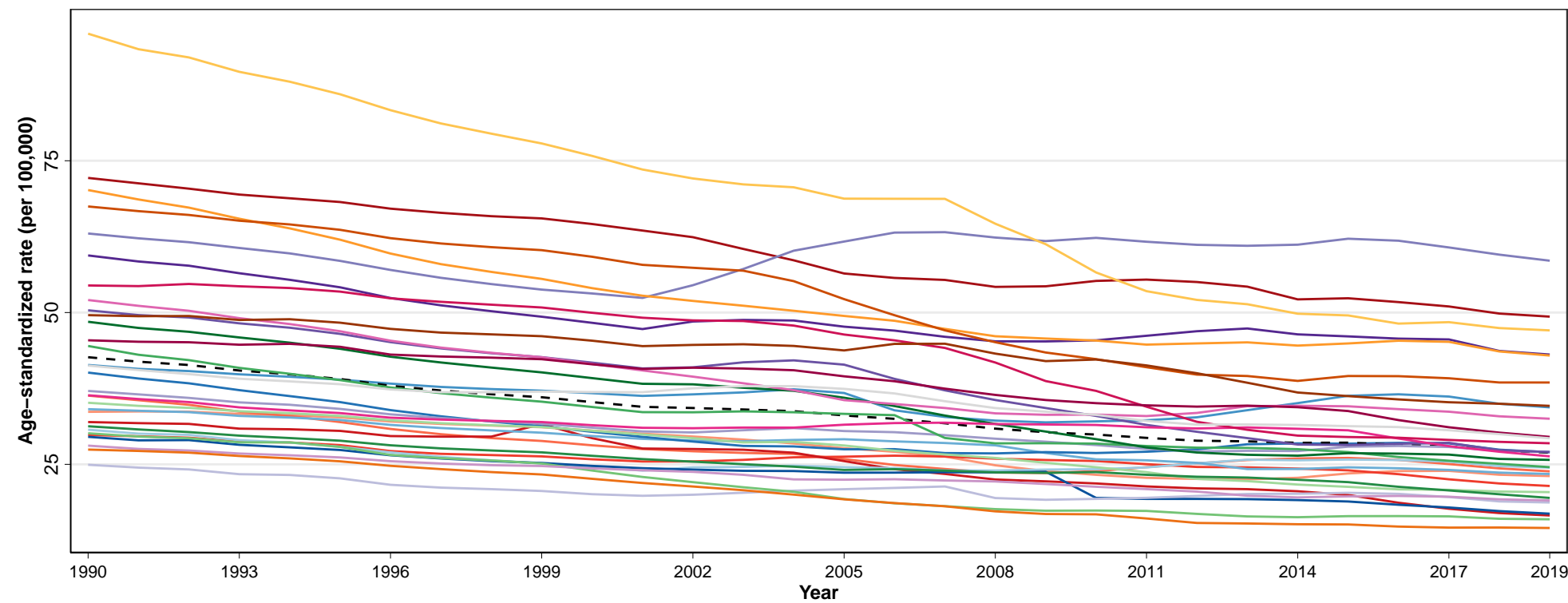

DALYs

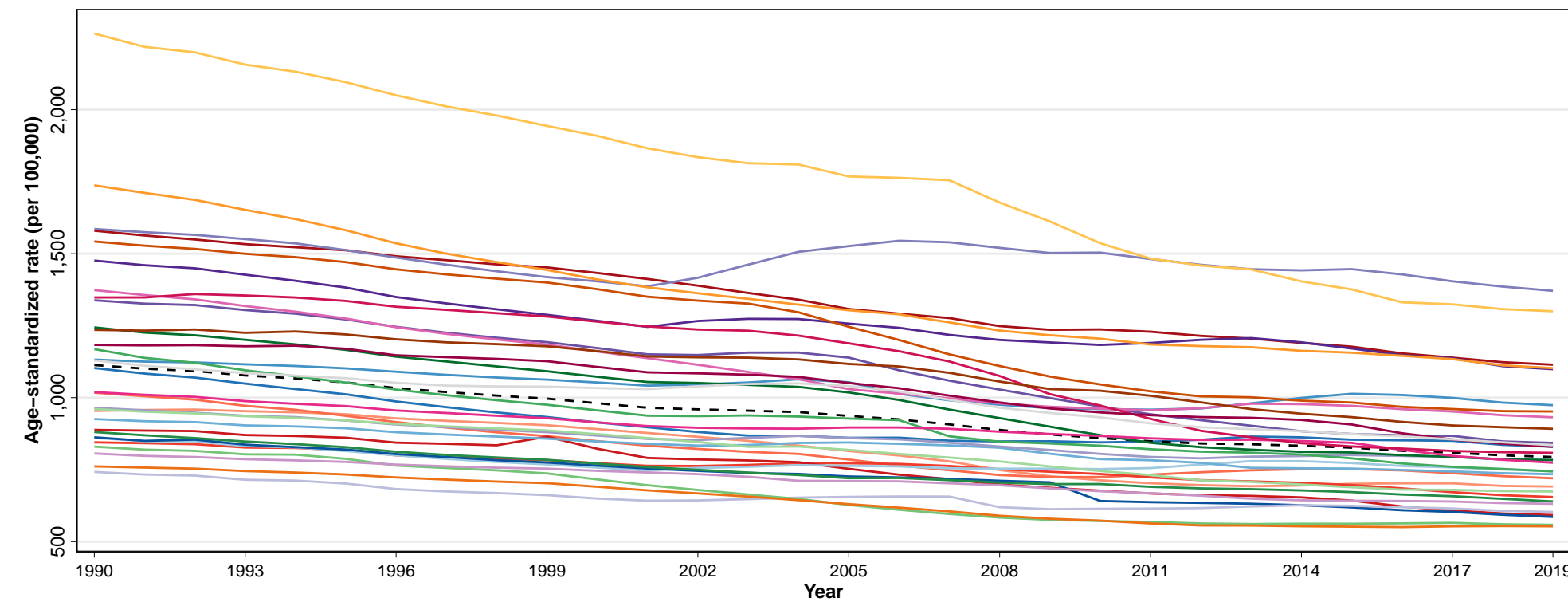

Supplement: Supplementary file 2 — Additional file 2: Figure S2. Burden rate by province during 1990-2019. [file 12931_2023_2353_MOESM2_ESM.pdf]

# Age-standardized

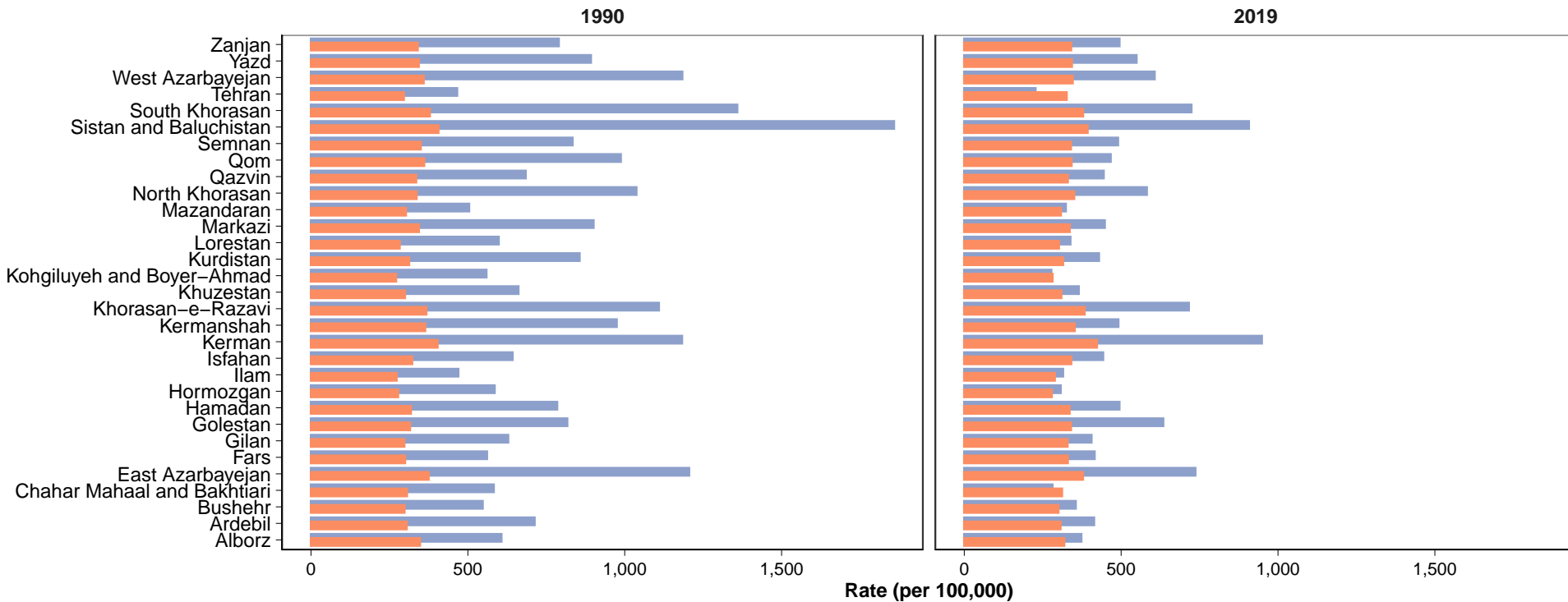

# <20 years

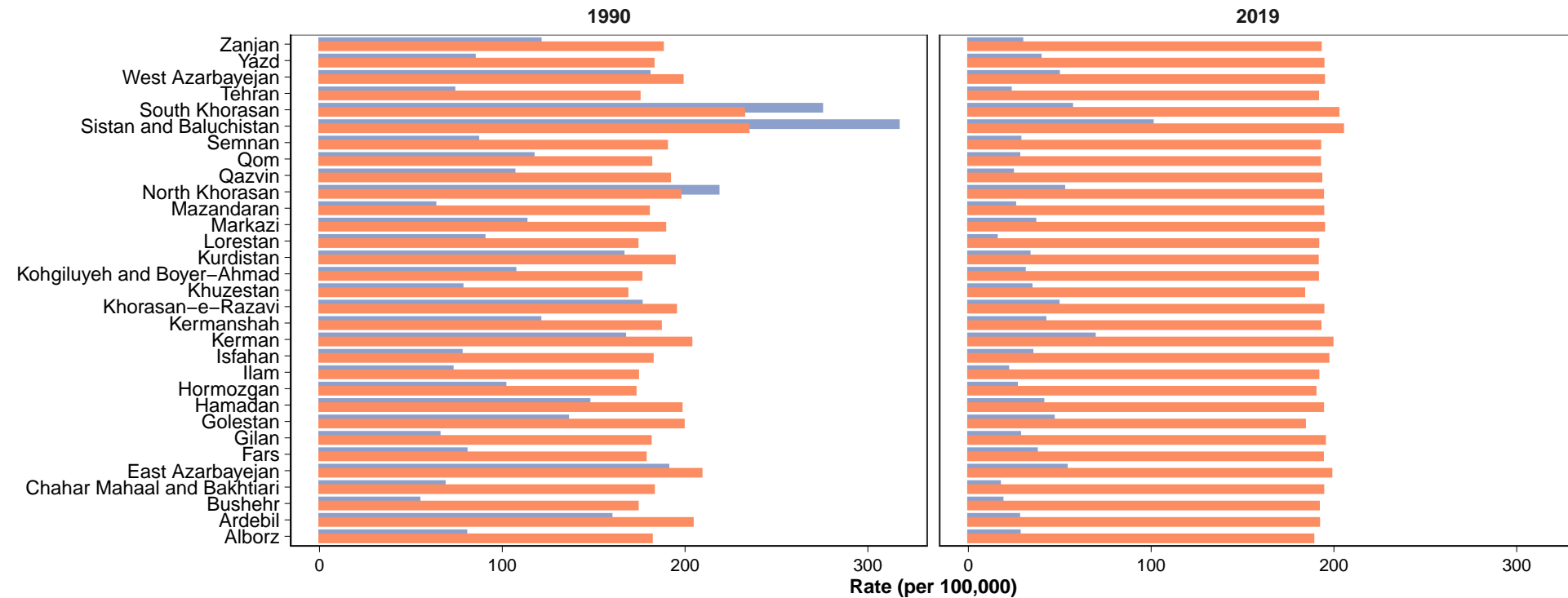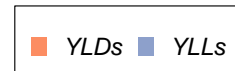

# 20 to 54 years

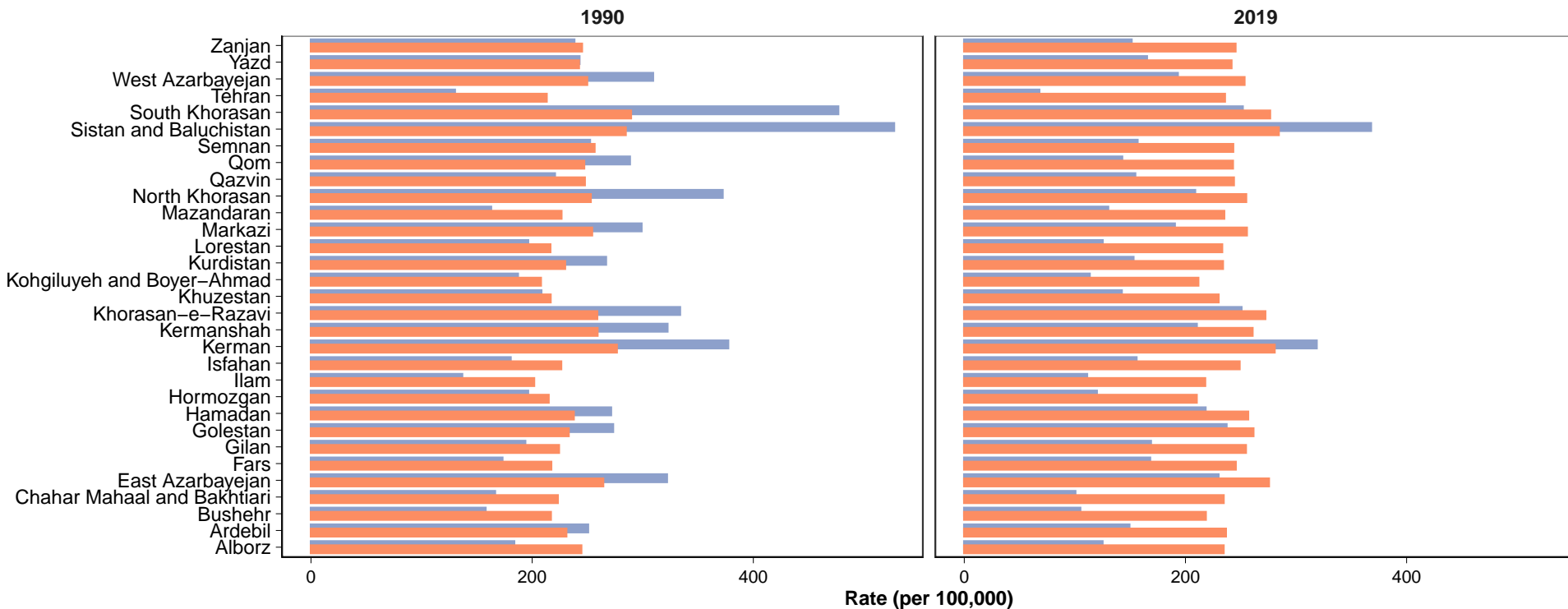

# 55 plus

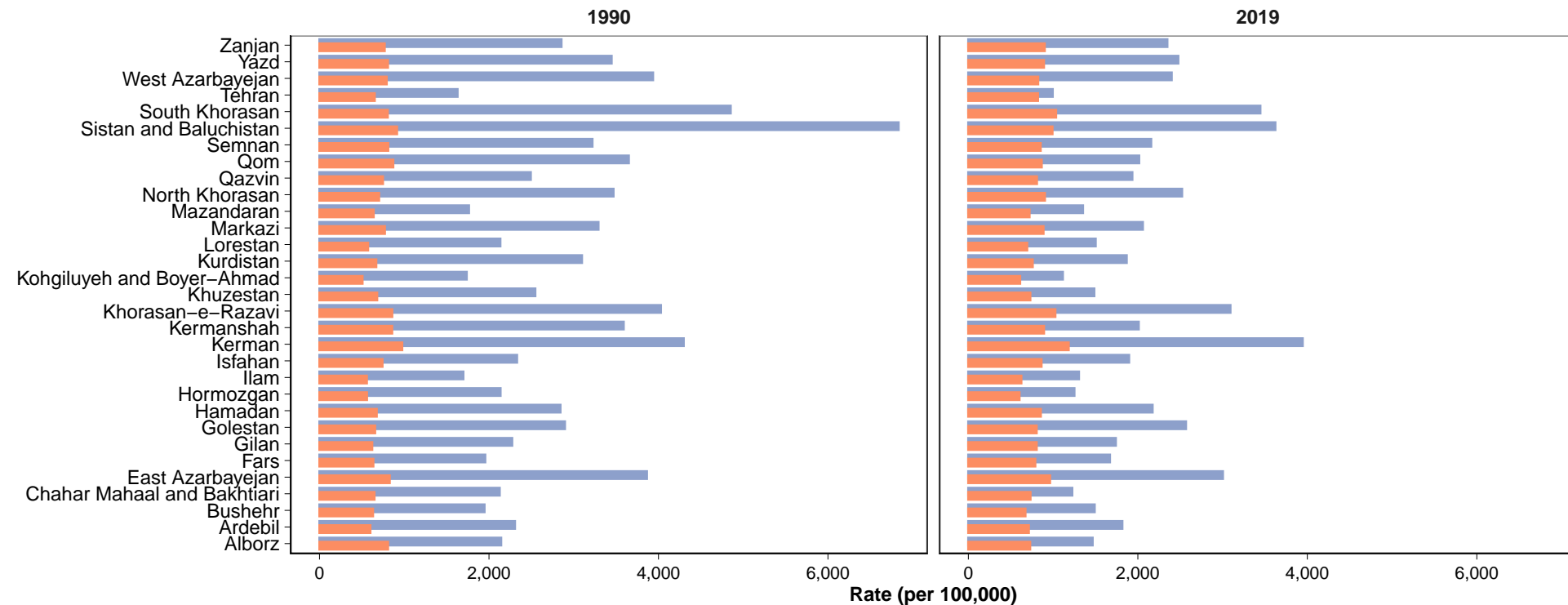

Supplement: Supplementary file 4 — Additional file 4: Figure S4. Comparison of YLDs and DALYs by province. [file 12931_2023_2353_MOESM4_ESM.pdf]
