# Supplementary material for: National, subnational and risk attributed burden of chronic respiratory diseases in Iran from 1990 to 2019
Source: Respir Res. 2023 Mar 11;24:74. doi: 10.1186/s12931-023-02353-1 (PMC10006557; doi:10.1186/s12931-023-02353-1)
Supplement: Supplementary file 3 — Additional file 3: Figure S3. The rate of burden measures by sex and age group at subnational level. 1990 vs 2019. [file 12931_2023_2353_MOESM3_ESM.pdf]

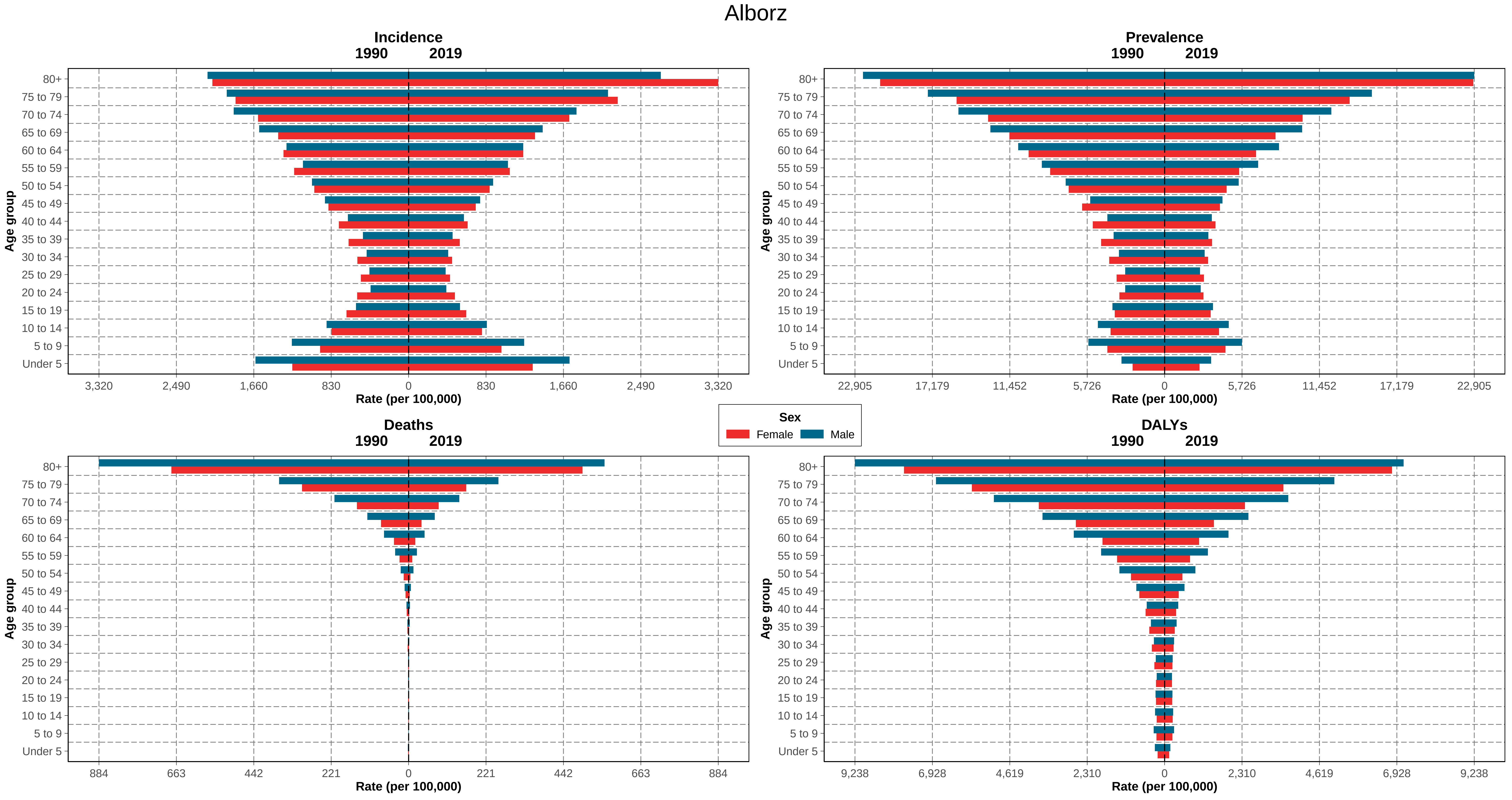

Alborz
Incidence
1990
2019
Prevalence
1990
2019
Deaths
1990
2019
DALYs
1990
2019
Age group
80+
75 to 79
70 to 74
65 to 69
60 to 64
55 to 59
50 to 54
45 to 49
40 to 44
35 to 39
30 to 34
25 to 29
20 to 24
15 to 19
10 to 14
5 to 9
Under 5
3,320
2,490
1,660
830
0
830
1,660
2,490
3,320
22,905
17,179
11,452
5,726
0
5,726
11,452
17,179
22,905
884
663
442
221
0
221
442
663
884
9,238
6,928
4,619
2,310
0
2,310
4,619
6,928
9,238
Rate (per 100,000)
Sex
Female
Male

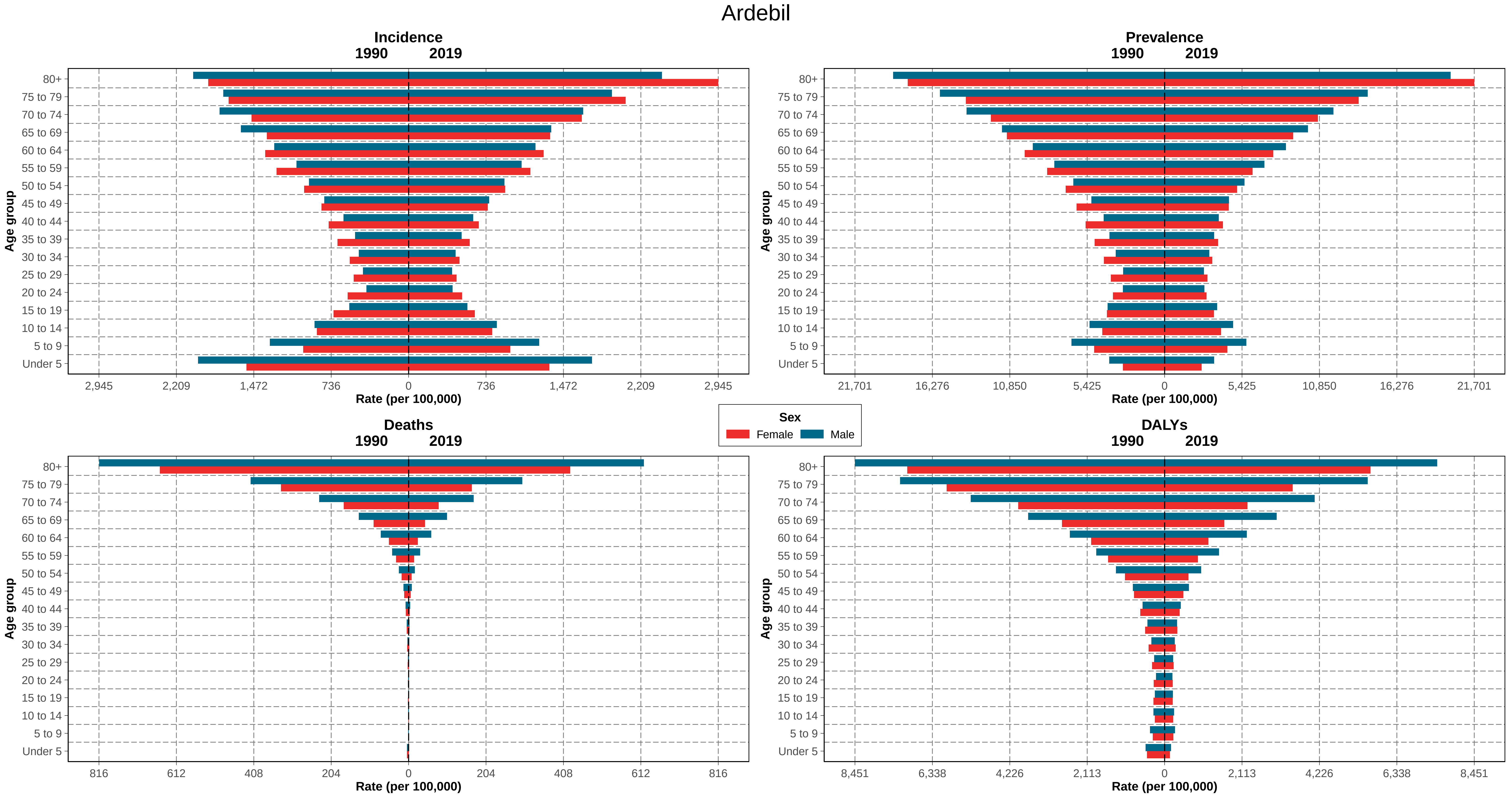

Ardebil
Incidence
1990 2019
Prevalence
1990 2019
Deaths
1990 2019
DALYs
1990 2019
Age group
80+
75 to 79
70 to 74
65 to 69
60 to 64
55 to 59
50 to 54
45 to 49
40 to 44
35 to 39
30 to 34
25 to 29
20 to 24
15 to 19
10 to 14
5 to 9
Under 5
2,945 2,209 1,472 736 0 736 1,472 2,209 2,945
21,701 16,276 10,850 5,425 0 5,425 10,850 16,276 21,701
816 612 408 204 0 204 408 612 816
8,451 6,338 4,226 2,113 0 2,113 4,226 6,338 8,451
Rate (per 100,000)
Sex
Female
Male

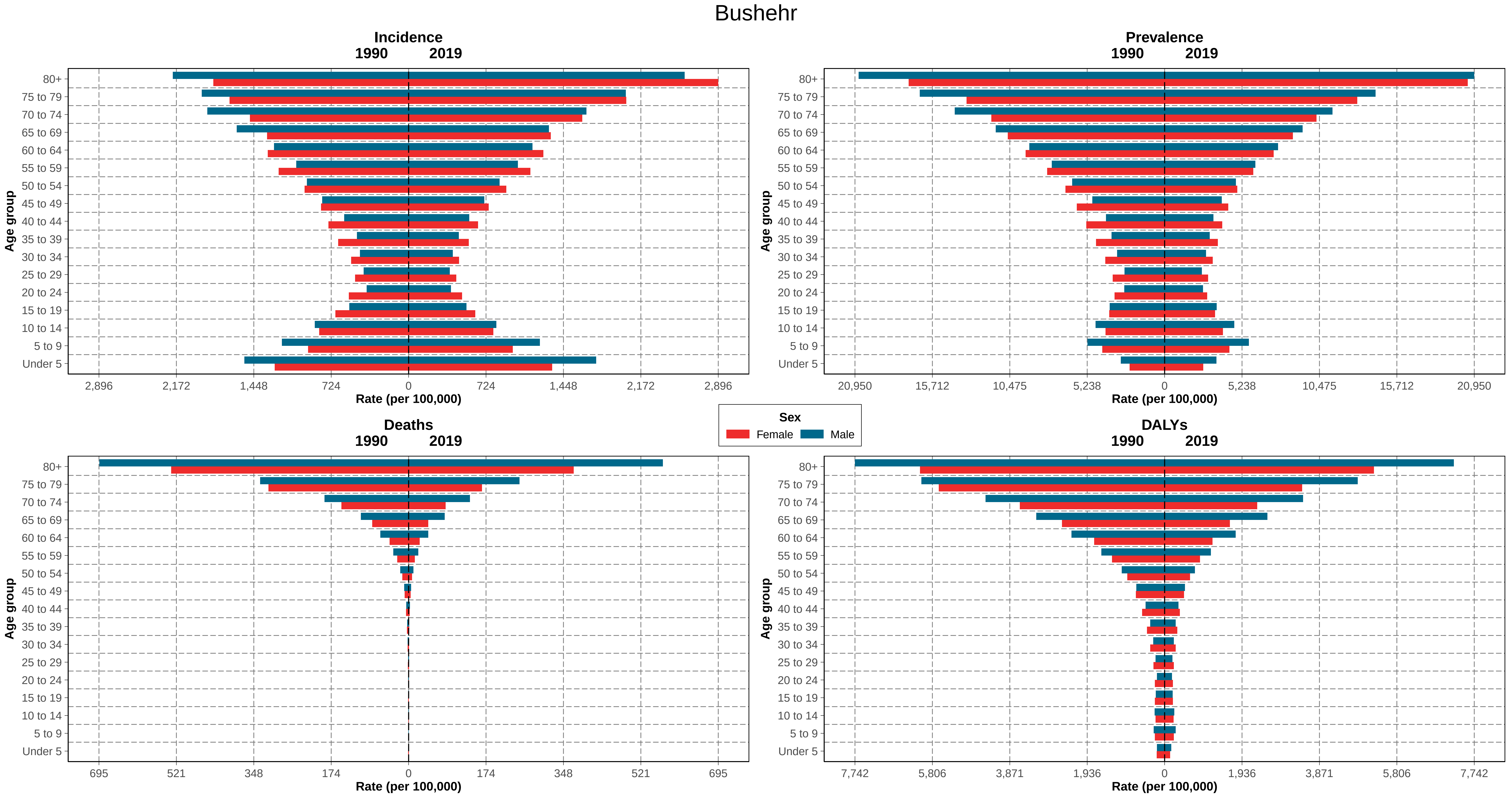

Bushehr
Incidence
1990
2019
Prevalence
1990
2019
Deaths
1990
2019
DALYs
1990
2019
Age group
80+
75 to 79
70 to 74
65 to 69
60 to 64
55 to 59
50 to 54
45 to 49
40 to 44
35 to 39
30 to 34
25 to 29
20 to 24
15 to 19
10 to 14
5 to 9
Under 5
2,896
2,172
1,448
724
0
724
1,448
2,172
2,896
20,950
15,712
10,475
5,238
0
5,238
10,475
15,712
20,950
695
521
348
174
0
174
348
521
695
7,742
5,806
3,871
1,936
0
1,936
3,871
5,806
7,742
Rate (per 100,000)
Sex
Female
Male

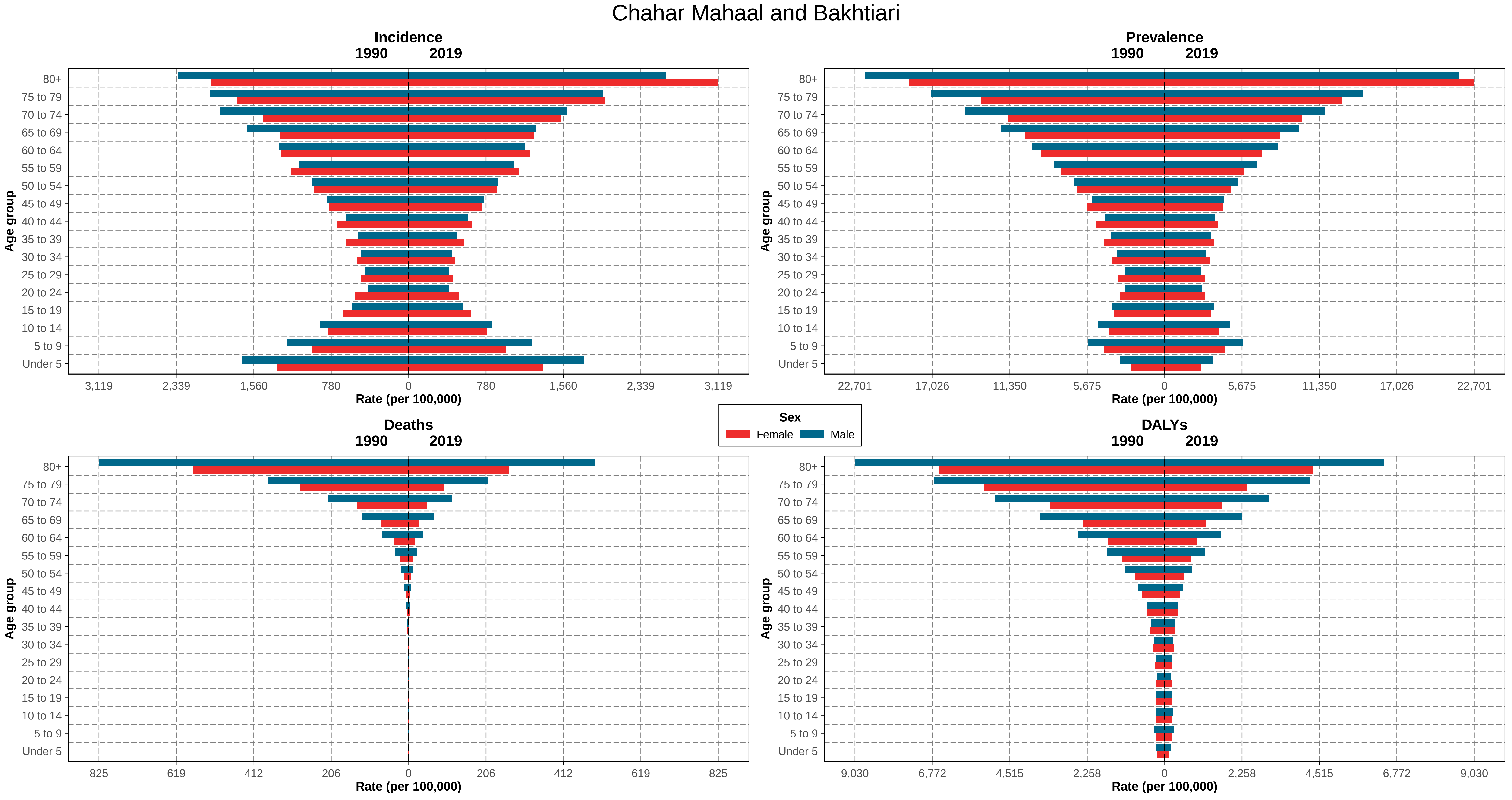

Chahar Mahaal and Bakhtiari
Incidence
1990 2019
Prevalence
1990 2019
Deaths
1990 2019
DALYs
1990 2019
Age group
80+
75 to 79
70 to 74
65 to 69
60 to 64
55 to 59
50 to 54
45 to 49
40 to 44
35 to 39
30 to 34
25 to 29
20 to 24
15 to 19
10 to 14
5 to 9
Under 5
3,119 2,339 1,560 780 0 780 1,560 2,339 3,119
22,701 17,026 11,350 5,675 0 5,675 11,350 17,026 22,701
825 619 412 206 0 206 412 619 825
9,030 6,772 4,515 2,258 0 2,258 4,515 6,772 9,030
Rate (per 100,000)
Sex
Female
Male

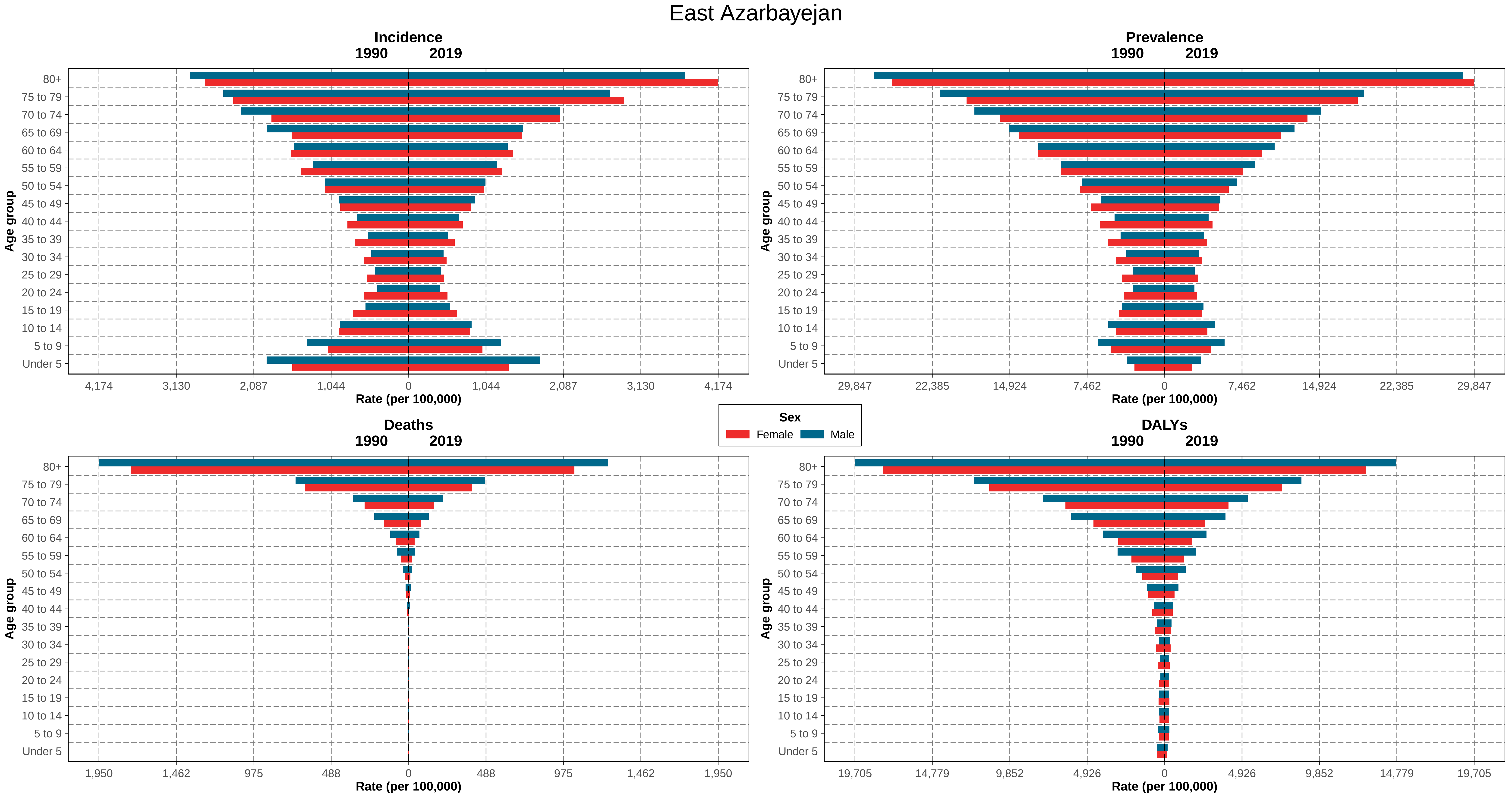
East Azarbayejan
Incidence
1990
2019
Prevalence
1990
2019
Deaths
1990
2019
DALYs
1990
2019
Age group
80+
75 to 79
70 to 74
65 to 69
60 to 64
55 to 59
50 to 54
45 to 49
40 to 44
35 to 39
30 to 34
25 to 29
20 to 24
15 to 19
10 to 14
5 to 9
Under 5
4,174
3,130
2,087
1,044
0
1,044
2,087
3,130
4,174
29,847
22,385
14,924
7,462
0
7,462
14,924
22,385
29,847
1,950
1,462
975
488
0
488
975
1,462
1,950
19,705
14,779
9,852
4,926
0
4,926
9,852
14,779
19,705
Rate (per 100,000)
Sex
Female
Male

Fars

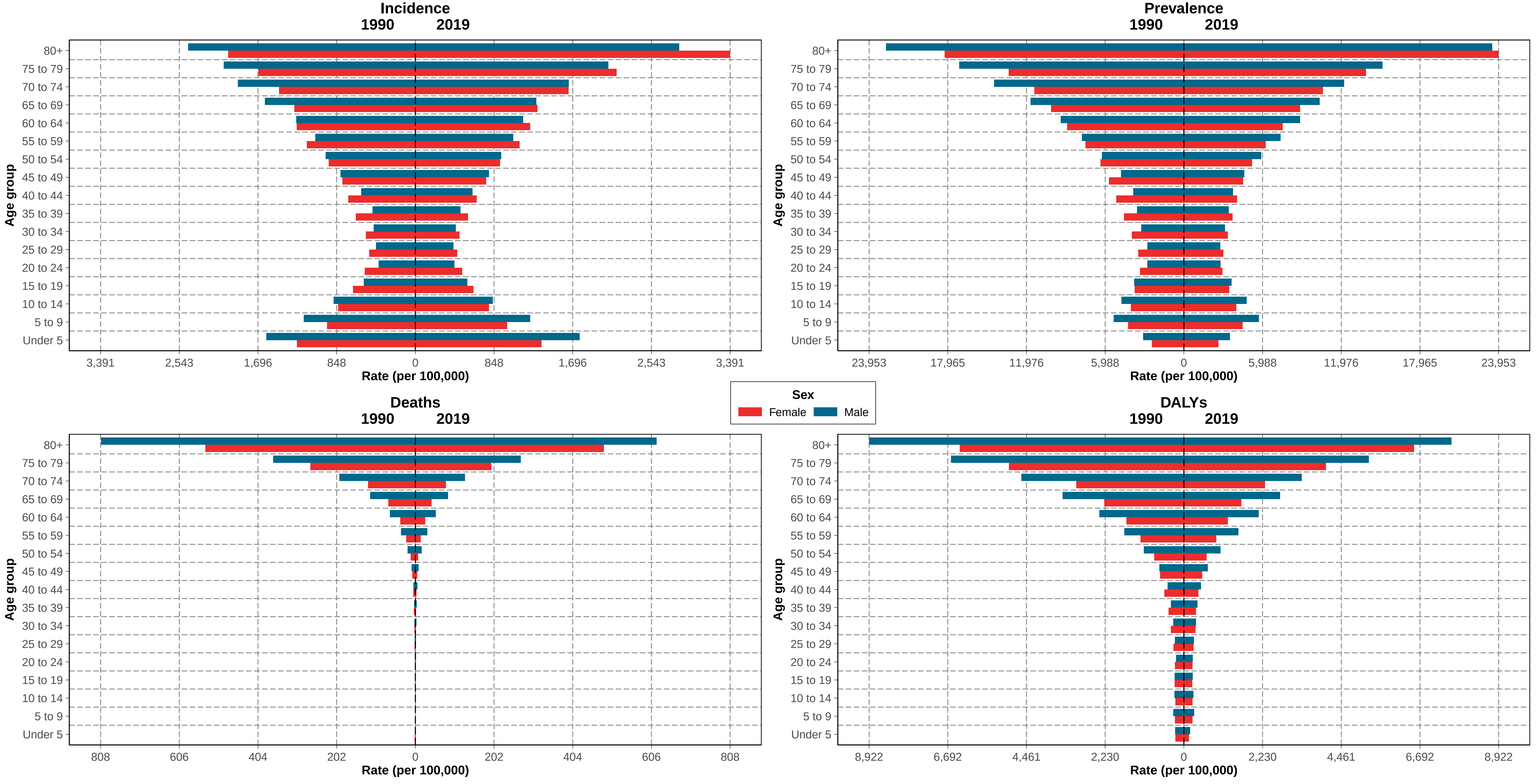
Incidence
1990
2019
Prevalence
Deaths
DALYs
Age group
80+
75 to 79
70 to 74
65 to 69
60 to 64
55 to 59
50 to 54
45 to 49
40 to 44
35 to 39
30 to 34
25 to 29
20 to 24
15 to 19
10 to 14
5 to 9
Under 5
3,391
2,543
1,696
848
0
23,953
17,965
11,976
5,988
808
606
404
202
8,922
6,692
4,461
2,230
Rate (per 100,000)
Sex
Female
Male

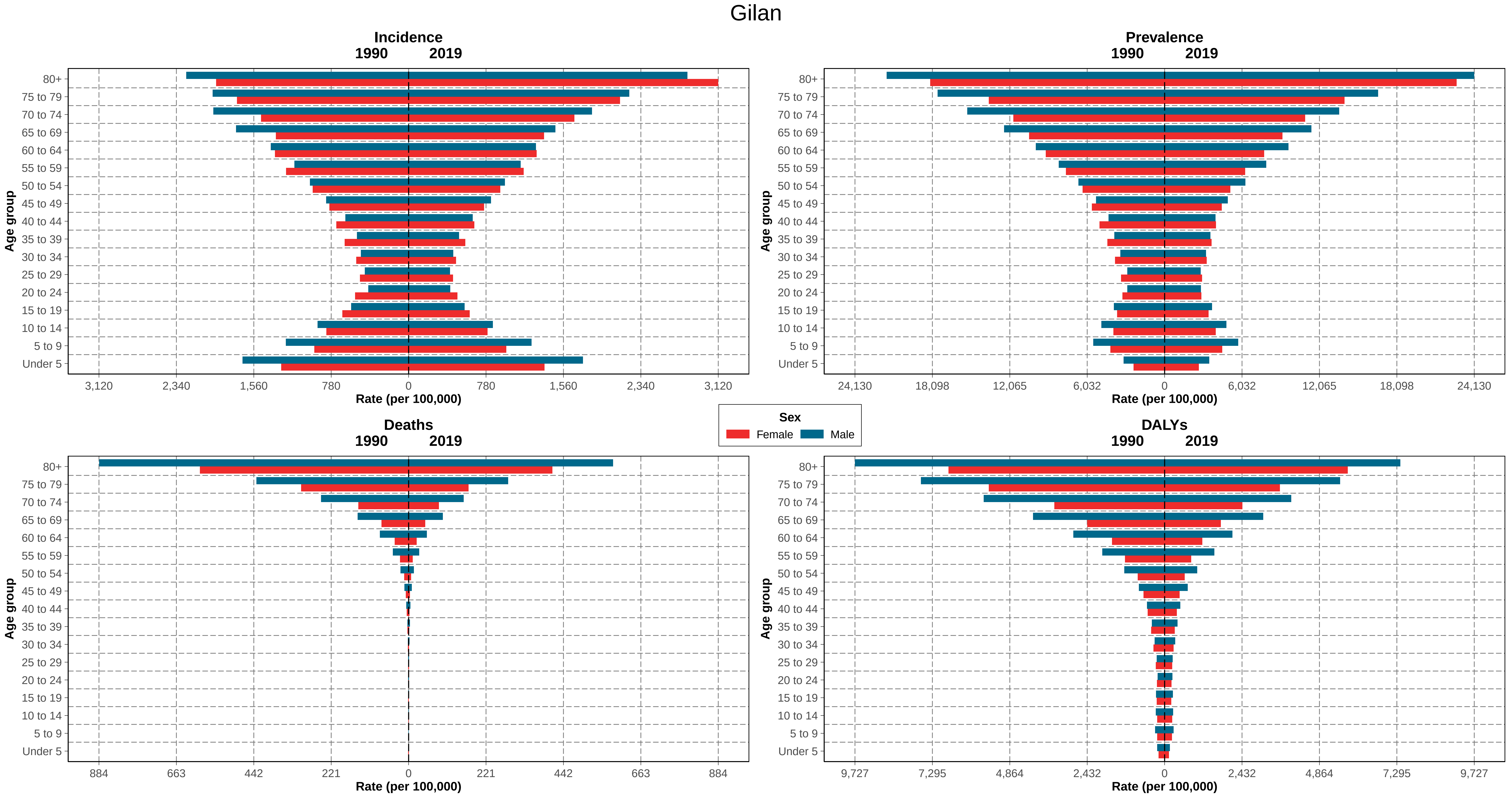
Gilan
Incidence
1990 2019
Prevalence
1990 2019
Deaths
1990 2019
DALYs
1990 2019
80+
75 to 79
70 to 74
65 to 69
60 to 64
55 to 59
50 to 54
45 to 49
40 to 44
35 to 39
30 to 34
25 to 29
20 to 24
15 to 19
10 to 14
5 to 9
Under 5
Age group
3,120 2,340 1,560 780 0 780 1,560 2,340 3,120
24,130 18,098 12,065 6,032 0 6,032 12,065 18,098 24,130
884 663 442 221 0 221 442 663 884
9,727 7,295 4,864 2,432 0 2,432 4,864 7,295 9,727
Rate (per 100,000)
Sex
Female
Male

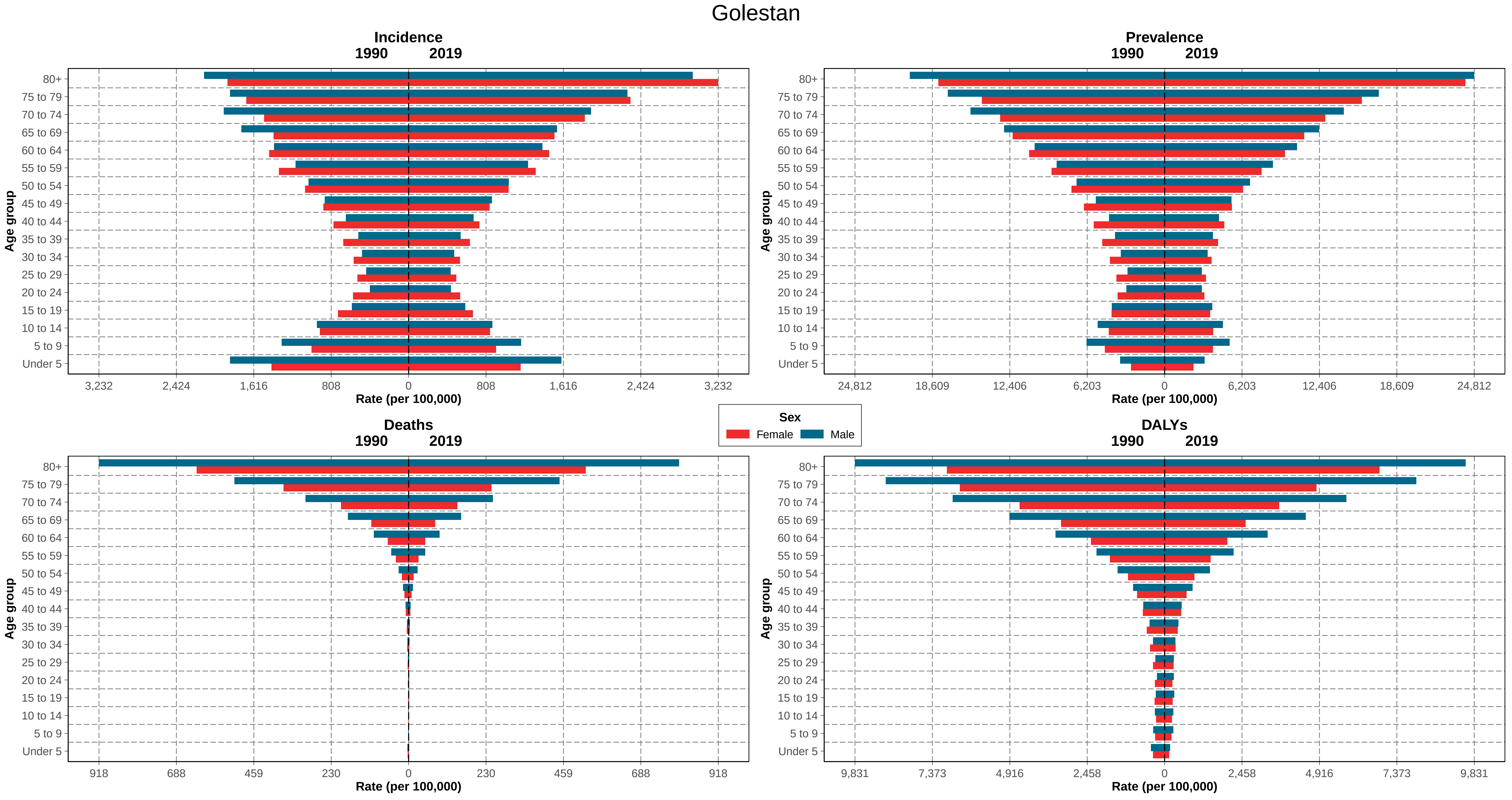

Golestan
Incidence
1990
2019
Prevalence
1990
2019
Deaths
1990
2019
DALYs
1990
2019
Age group
80+
75 to 79
70 to 74
65 to 69
60 to 64
55 to 59
50 to 54
45 to 49
40 to 44
35 to 39
30 to 34
25 to 29
20 to 24
15 to 19
10 to 14
5 to 9
Under 5
3,232
2,424
1,616
808
0
808
1,616
2,424
3,232
24,812
18,609
12,406
6,203
0
6,203
12,406
18,609
24,812
918
688
459
230
0
230
459
688
918
9,831
7,373
4,916
2,458
0
2,458
4,916
7,373
9,831
Rate (per 100,000)
Sex
Female
Male

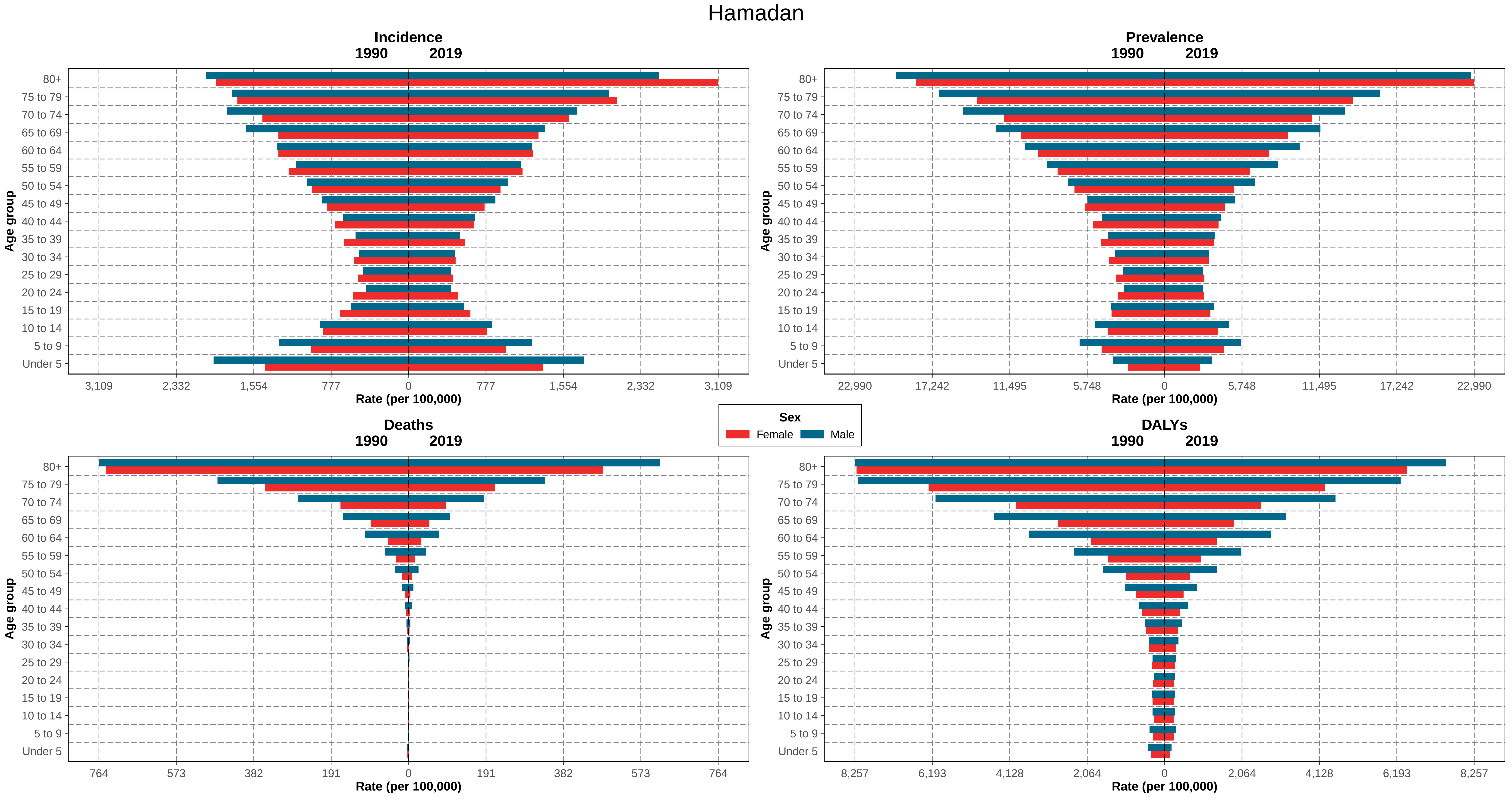

Hamadan
Incidence
1990
2019
Prevalence
1990
2019
Deaths
1990
2019
DALYs
1990
2019
Age group
80+
75 to 79
70 to 74
65 to 69
60 to 64
55 to 59
50 to 54
45 to 49
40 to 44
35 to 39
30 to 34
25 to 29
20 to 24
15 to 19
10 to 14
5 to 9
Under 5
3,109
2,332
1,554
777
0
777
1,554
2,332
3,109
22,990
17,242
11,495
5,748
0
5,748
11,495
17,242
22,990
764
573
382
191
0
191
382
573
764
8,257
6,193
4,128
2,064
0
2,064
4,128
6,193
8,257
Rate (per 100,000)
Sex
Female
Male

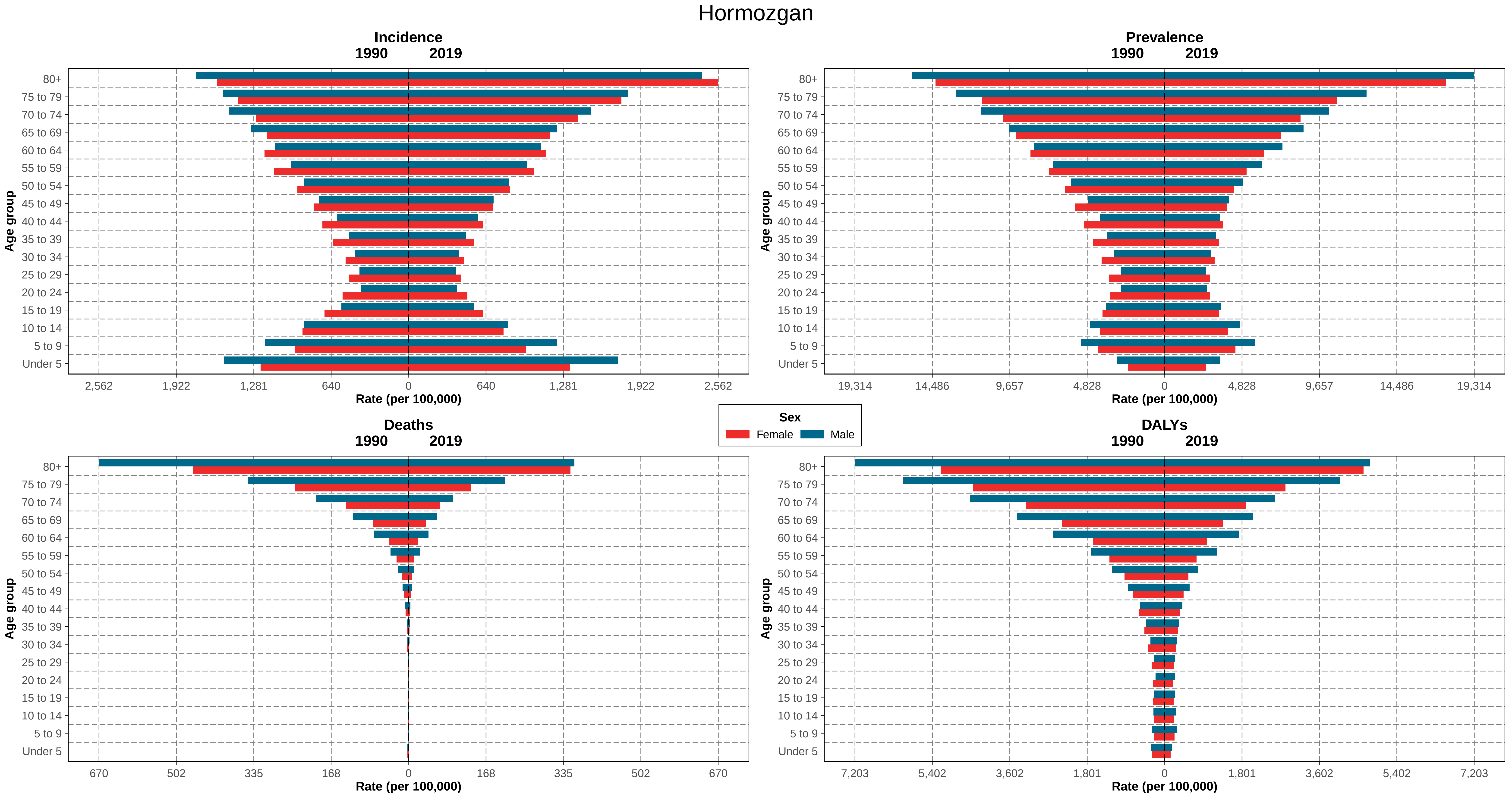
Hormozgan
Incidence
1990 2019
Prevalence
1990 2019
Deaths
1990 2019
DALYs
1990 2019
Age group
80+
75 to 79
70 to 74
65 to 69
60 to 64
55 to 59
50 to 54
45 to 49
40 to 44
35 to 39
30 to 34
25 to 29
20 to 24
15 to 19
10 to 14
5 to 9
Under 5
Rate (per 100,000)
2,562 1,922 1,281 640 0 640 1,281 1,922 2,562
19,314 14,486 9,657 4,828 0 4,828 9,657 14,486 19,314
670 502 335 168 0 168 335 502 670
7,203 5,402 3,602 1,801 0 1,801 3,602 5,402 7,203
Sex
Female
Male

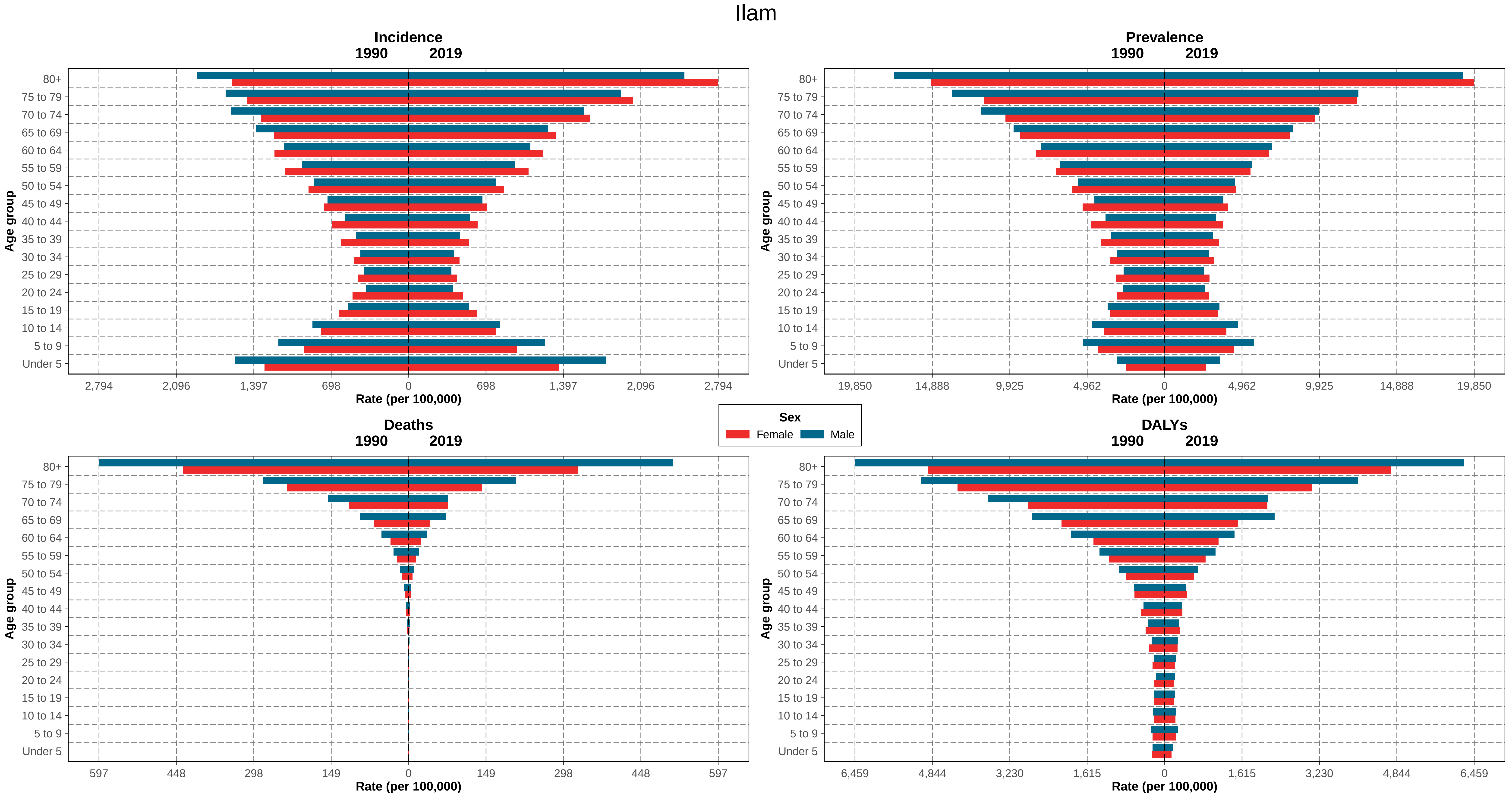
Ilam
Incidence
1990
2019
Prevalence
1990
2019
Deaths
1990
2019
DALYs
1990
2019
Age group
80+
75 to 79
70 to 74
65 to 69
60 to 64
55 to 59
50 to 54
45 to 49
40 to 44
35 to 39
30 to 34
25 to 29
20 to 24
15 to 19
10 to 14
5 to 9
Under 5
Rate (per 100,000)
2,794
2,096
1,397
698
0
698
1,397
2,096
2,794
19,850
14,888
9,925
4,962
0
4,962
9,925
14,888
19,850
597
448
298
149
0
149
298
448
597
6,459
4,844
3,230
1,615
0
1,615
3,230
4,844
6,459
Sex
Female
Male

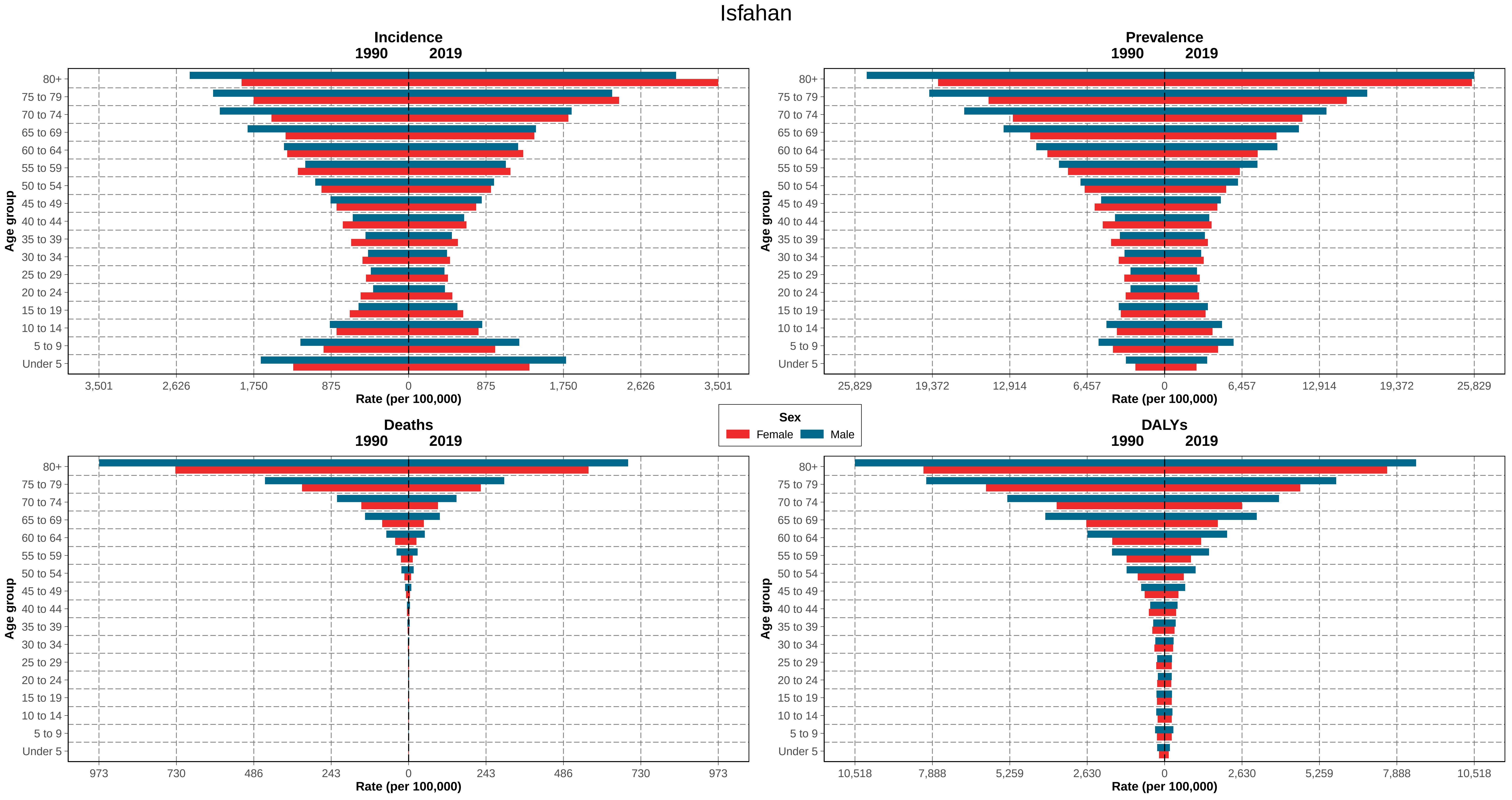

Isfahan
Incidence
1990
2019
Prevalence
1990
2019
Deaths
1990
2019
DALYs
1990
2019
Age group
80+
75 to 79
70 to 74
65 to 69
60 to 64
55 to 59
50 to 54
45 to 49
40 to 44
35 to 39
30 to 34
25 to 29
20 to 24
15 to 19
10 to 14
5 to 9
Under 5
3,501
2,626
1,750
875
0
875
1,750
2,626
3,501
25,829
19,372
12,914
6,457
0
6,457
12,914
19,372
25,829
973
730
486
243
0
243
486
730
973
10,518
7,888
5,259
2,630
0
2,630
5,259
7,888
10,518
Rate (per 100,000)
Sex
Female
Male

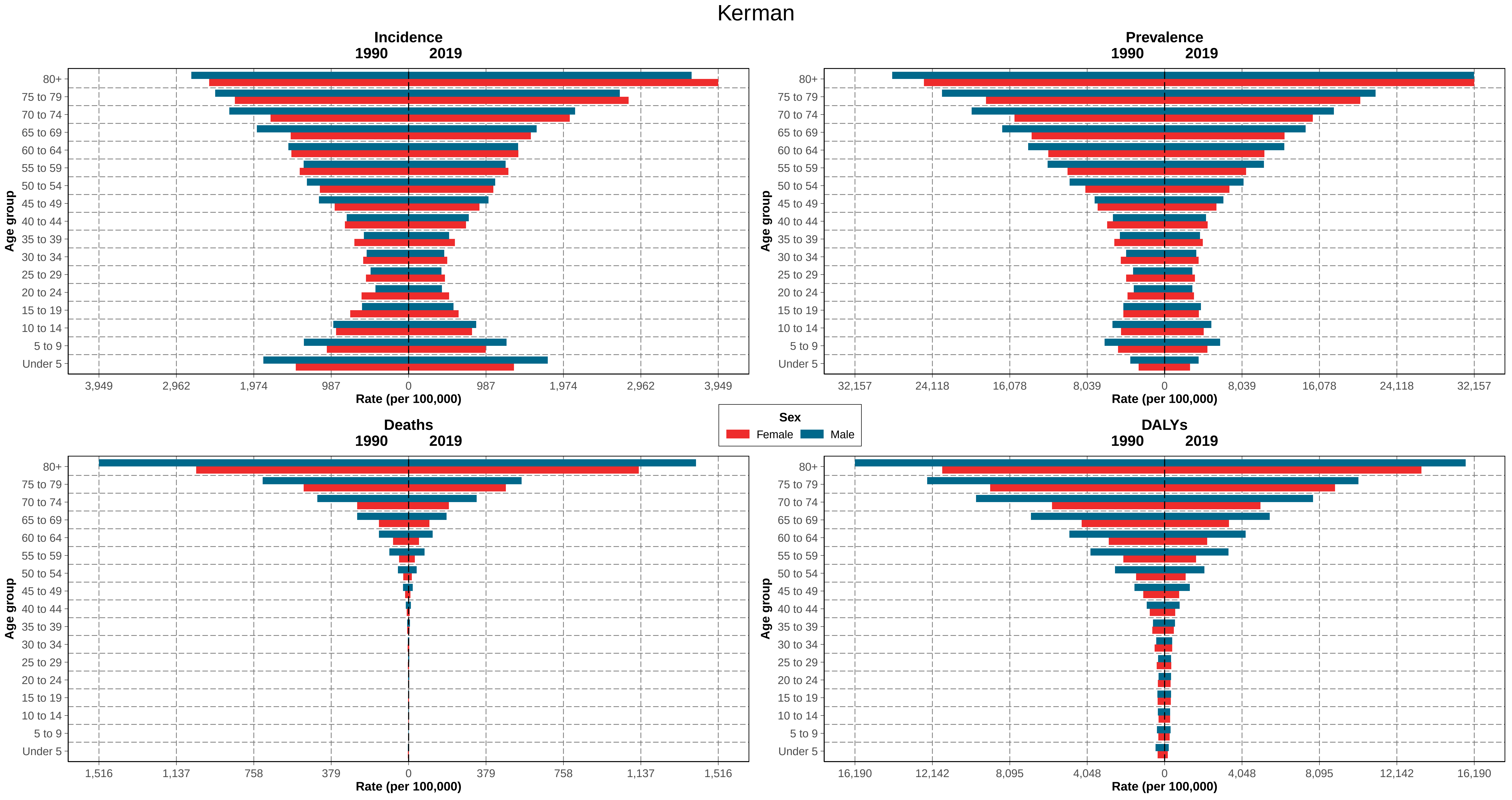

Kerman
Incidence
Prevalence
Deaths
DALYs
1990
2019
Age group
80+
75 to 79
70 to 74
65 to 69
60 to 64
55 to 59
50 to 54
45 to 49
40 to 44
35 to 39
30 to 34
25 to 29
20 to 24
15 to 19
10 to 14
5 to 9
Under 5
3,949
2,962
1,974
987
0
987
1,974
2,962
3,949
32,157
24,118
16,078
8,039
0
8,039
16,078
24,118
32,157
1,516
1,137
758
379
0
379
758
1,137
1,516
16,190
12,142
8,095
4,048
0
4,048
8,095
12,142
16,190
Rate (per 100,000)
Sex
Female
Male

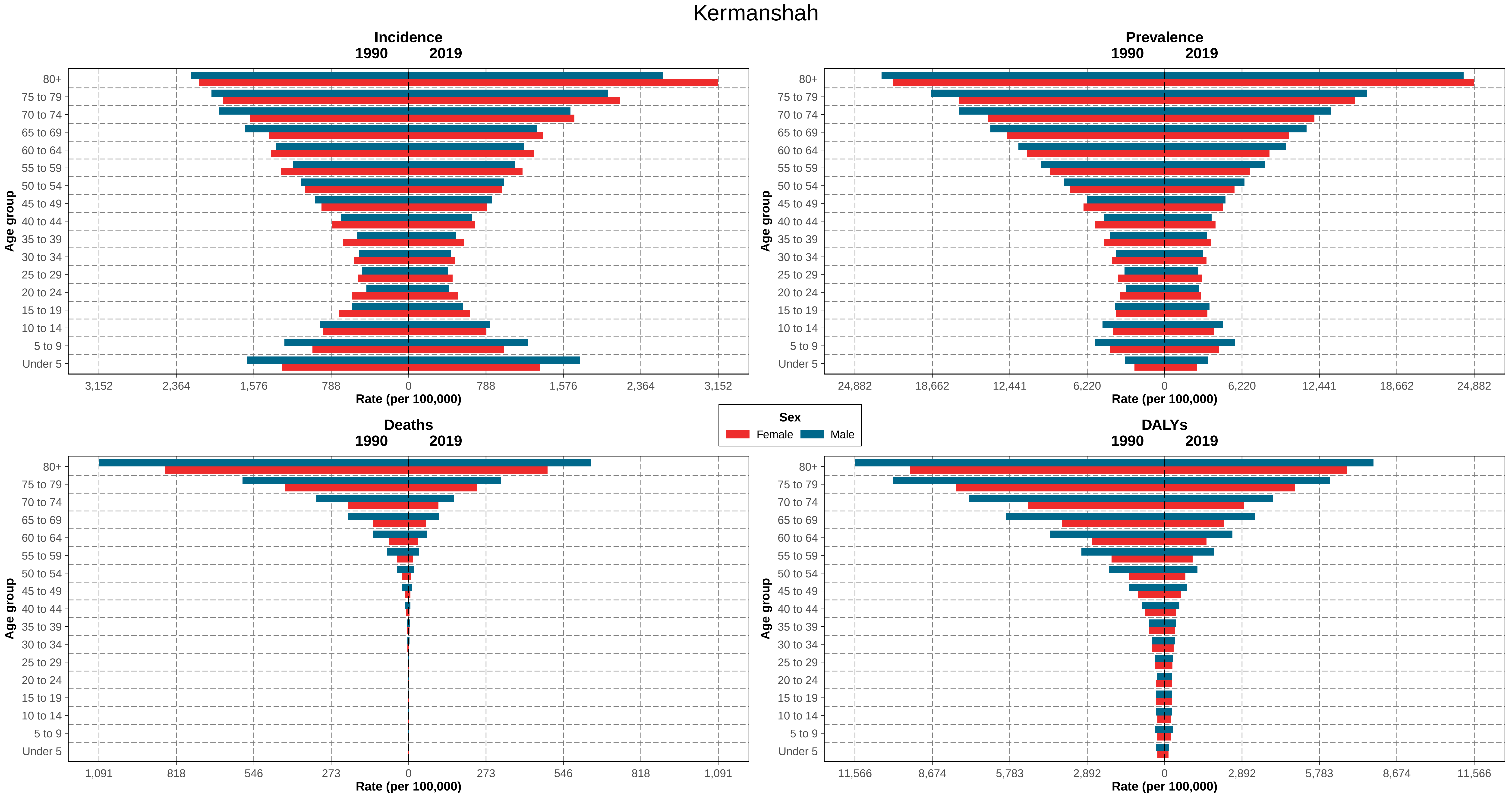
Kermanshah
Incidence
1990 2019
Prevalence
1990 2019
Deaths
1990 2019
DALYs
1990 2019
Age group
80+
75 to 79
70 to 74
65 to 69
60 to 64
55 to 59
50 to 54
45 to 49
40 to 44
35 to 39
30 to 34
25 to 29
20 to 24
15 to 19
10 to 14
5 to 9
Under 5
3,152 2,364 1,576 788 0 788 1,576 2,364 3,152
24,882 18,662 12,441 6,220 0 6,220 12,441 18,662 24,882
1,091 818 546 273 0 273 546 818 1,091
11,566 8,674 5,783 2,892 0 2,892 5,783 8,674 11,566
Rate (per 100,000)
Sex
Female
Male

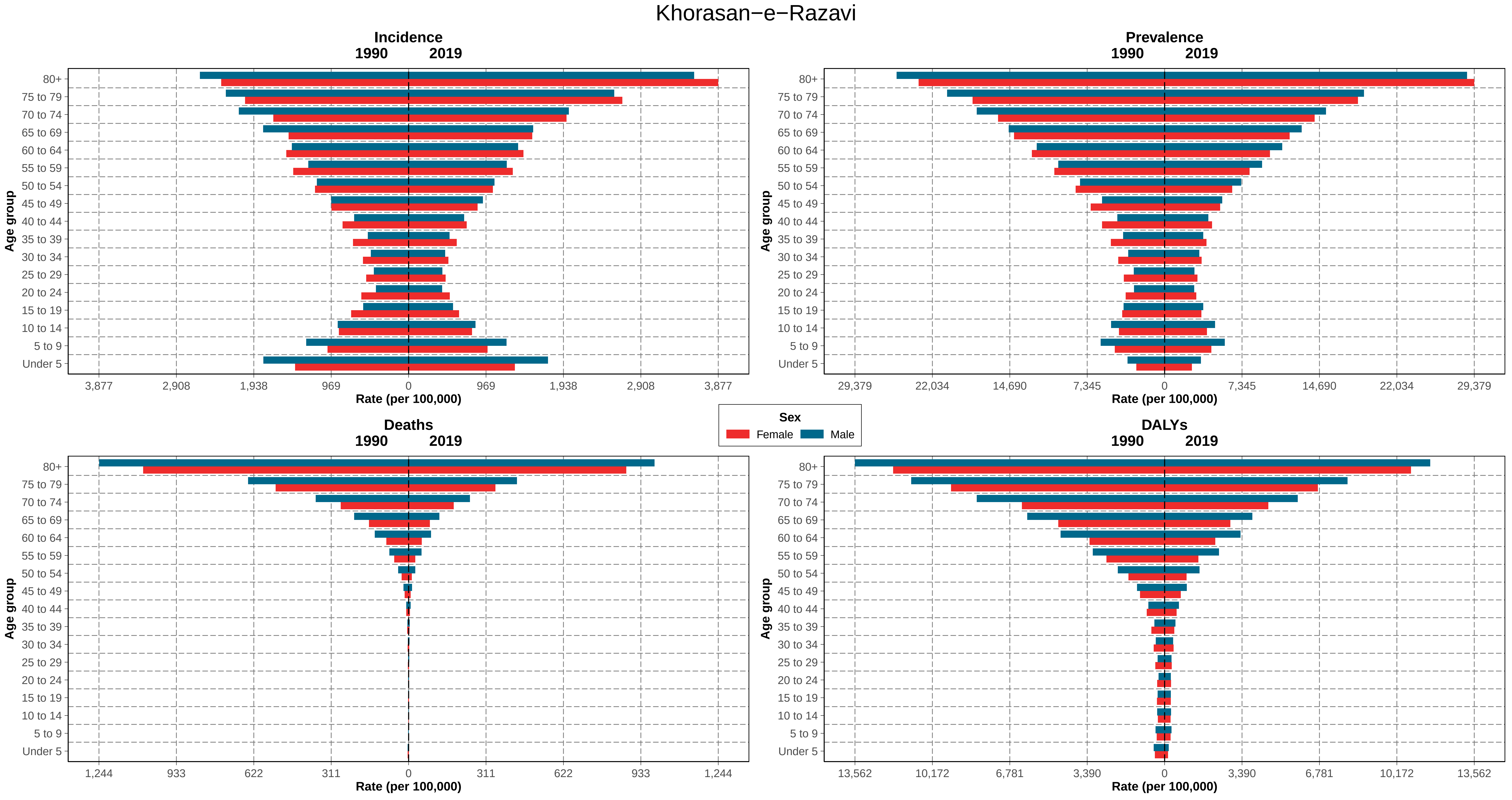
Khorasan−e−Razavi
Incidence
1990
2019
Prevalence
Deaths
DALYs
Age group
80+
75 to 79
70 to 74
65 to 69
60 to 64
55 to 59
50 to 54
45 to 49
40 to 44
35 to 39
30 to 34
25 to 29
20 to 24
15 to 19
10 to 14
5 to 9
Under 5
3,877
2,908
1,938
969
0
29,379
22,034
14,690
7,345
1,244
933
622
311
13,562
10,172
6,781
3,390
Rate (per 100,000)
Sex
Female
Male

Khuzestan

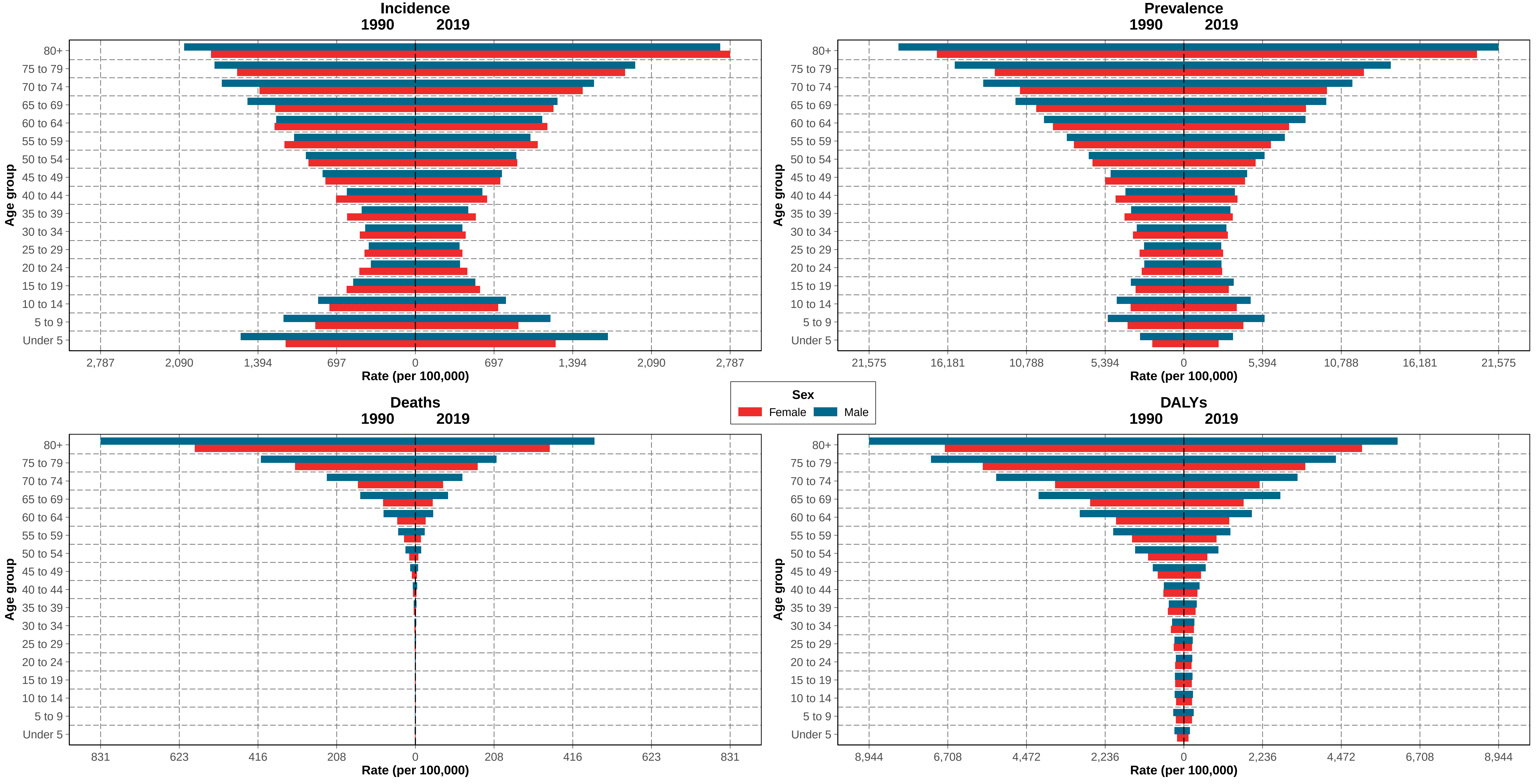

Incidence
1990
2019
Prevalence
1990
2019
Deaths
1990
2019
DALYs
1990
2019
Age group
80+
75 to 79
70 to 74
65 to 69
60 to 64
55 to 59
50 to 54
45 to 49
40 to 44
35 to 39
30 to 34
25 to 29
20 to 24
15 to 19
10 to 14
5 to 9
Under 5
2,787
2,090
1,394
697
0
697
1,394
2,090
2,787
21,575
16,181
10,788
5,394
0
5,394
10,788
16,181
21,575
831
623
416
208
0
208
416
623
831
8,944
6,708
4,472
2,236
0
2,236
4,472
6,708
8,944
Rate (per 100,000)
Sex
Female
Male

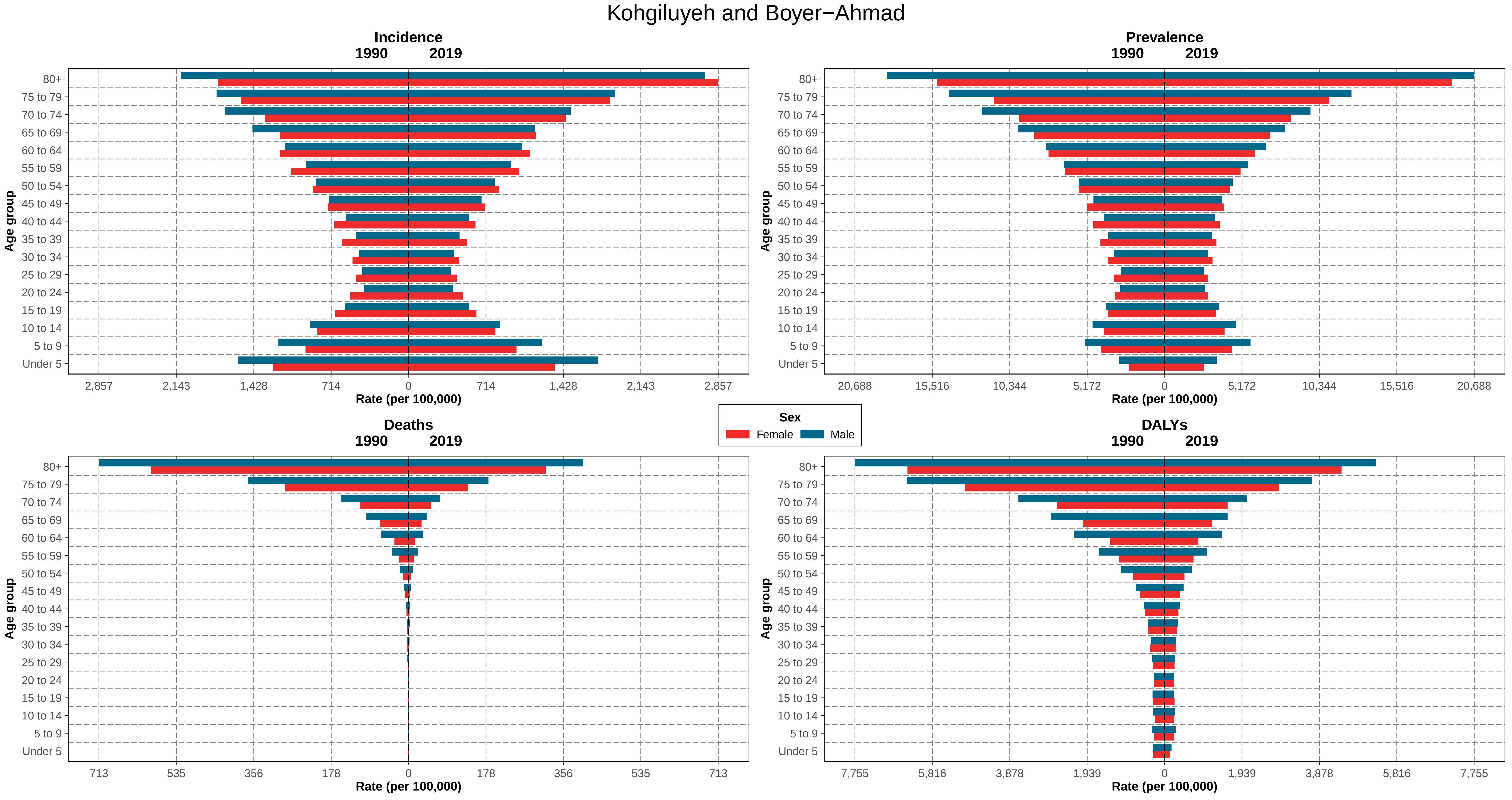
Kohgiluyeh and Boyer−Ahmad
Incidence
1990
2019
Prevalence
1990
2019
Deaths
1990
2019
DALYs
1990
2019
Age group
80+
75 to 79
70 to 74
65 to 69
60 to 64
55 to 59
50 to 54
45 to 49
40 to 44
35 to 39
30 to 34
25 to 29
20 to 24
15 to 19
10 to 14
5 to 9
Under 5
Rate (per 100,000)
2,857
2,143
1,428
714
0
20,688
15,516
10,344
5,172
713
535
356
178
7,755
5,816
3,878
1,939
Sex
Female
Male

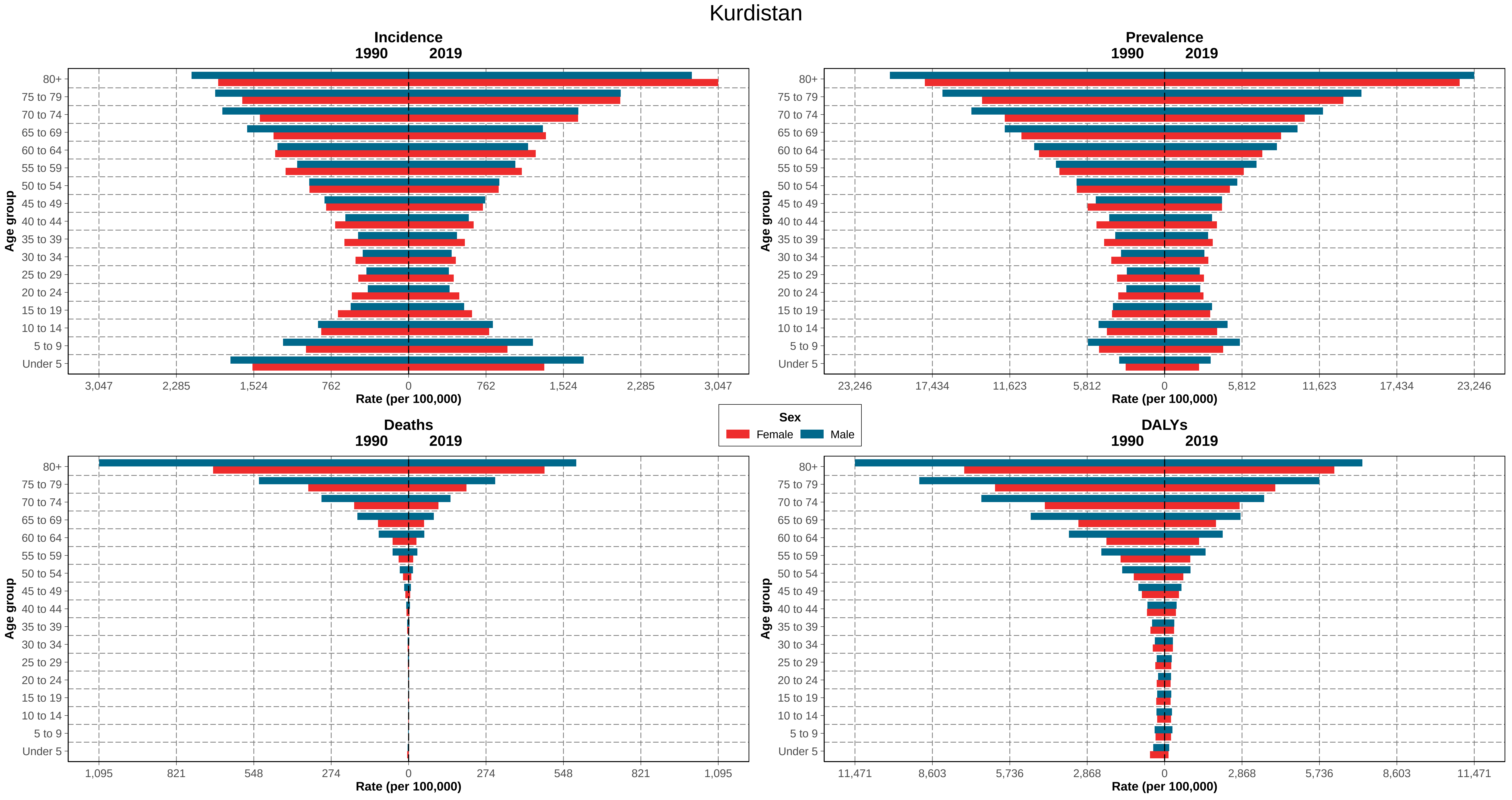
Kurdistan
Incidence
Prevalence
Deaths
DALYs
1990
2019
Age group
Rate (per 100,000)
80+
75 to 79
70 to 74
65 to 69
60 to 64
55 to 59
50 to 54
45 to 49
40 to 44
35 to 39
30 to 34
25 to 29
20 to 24
15 to 19
10 to 14
5 to 9
Under 5
3,047
2,285
1,524
762
0
23,246
17,434
11,623
5,812
1,095
821
548
274
11,471
8,603
5,736
2,868
Sex
Female
Male

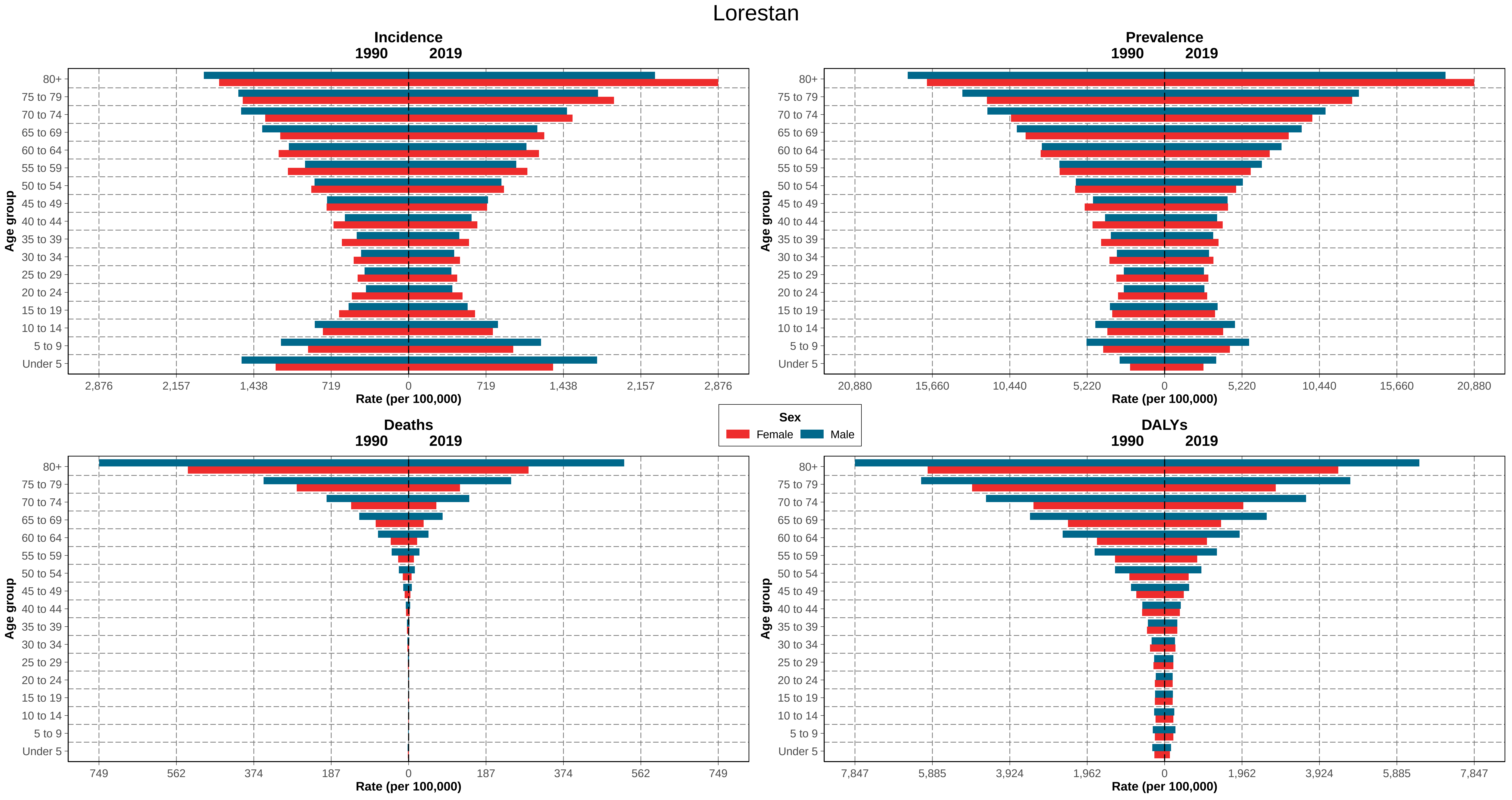

Lorestan
Incidence
1990
2019
Prevalence
1990
2019
Deaths
1990
2019
DALYs
1990
2019
Age group
80+
75 to 79
70 to 74
65 to 69
60 to 64
55 to 59
50 to 54
45 to 49
40 to 44
35 to 39
30 to 34
25 to 29
20 to 24
15 to 19
10 to 14
5 to 9
Under 5
2,876
2,157
1,438
719
0
20,880
15,660
10,440
5,220
749
562
374
187
7,847
5,885
3,924
1,962
Rate (per 100,000)
Sex
Female
Male

Markazi

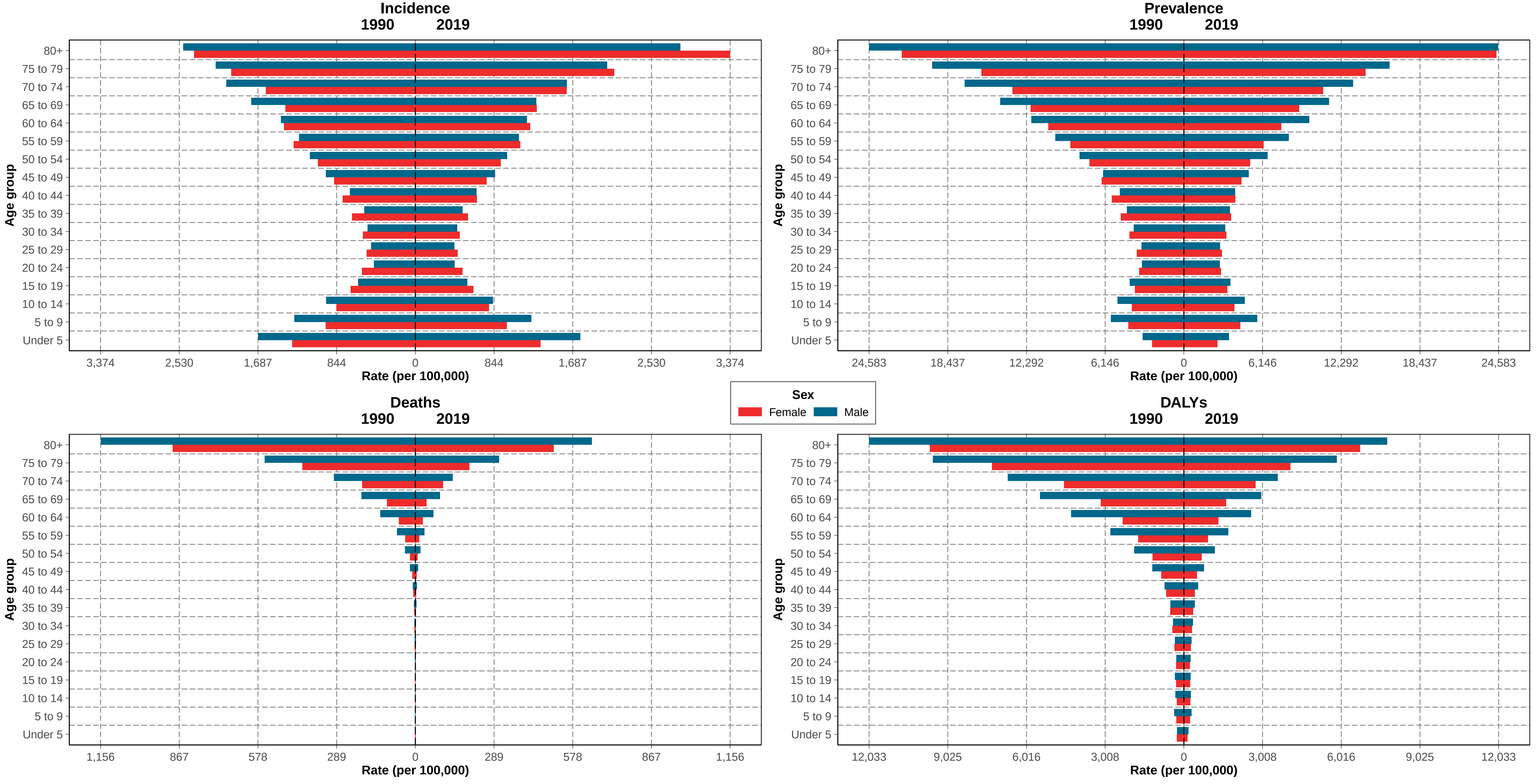
Incidence
1990 2019
Prevalence
1990 2019
Deaths
1990 2019
DALYs
1990 2019
80+
75 to 79
70 to 74
65 to 69
60 to 64
55 to 59
50 to 54
45 to 49
40 to 44
35 to 39
30 to 34
25 to 29
20 to 24
15 to 19
10 to 14
5 to 9
Under 5
Age group
3,374 2,530 1,687 844 0 844 1,687 2,530 3,374
24,583 18,437 12,292 6,146 0 6,146 12,292 18,437 24,583
1,156 867 578 289 0 289 578 867 1,156
12,033 9,025 6,016 3,008 0 3,008 6,016 9,025 12,033
Rate (per 100,000)
Sex
Female
Male

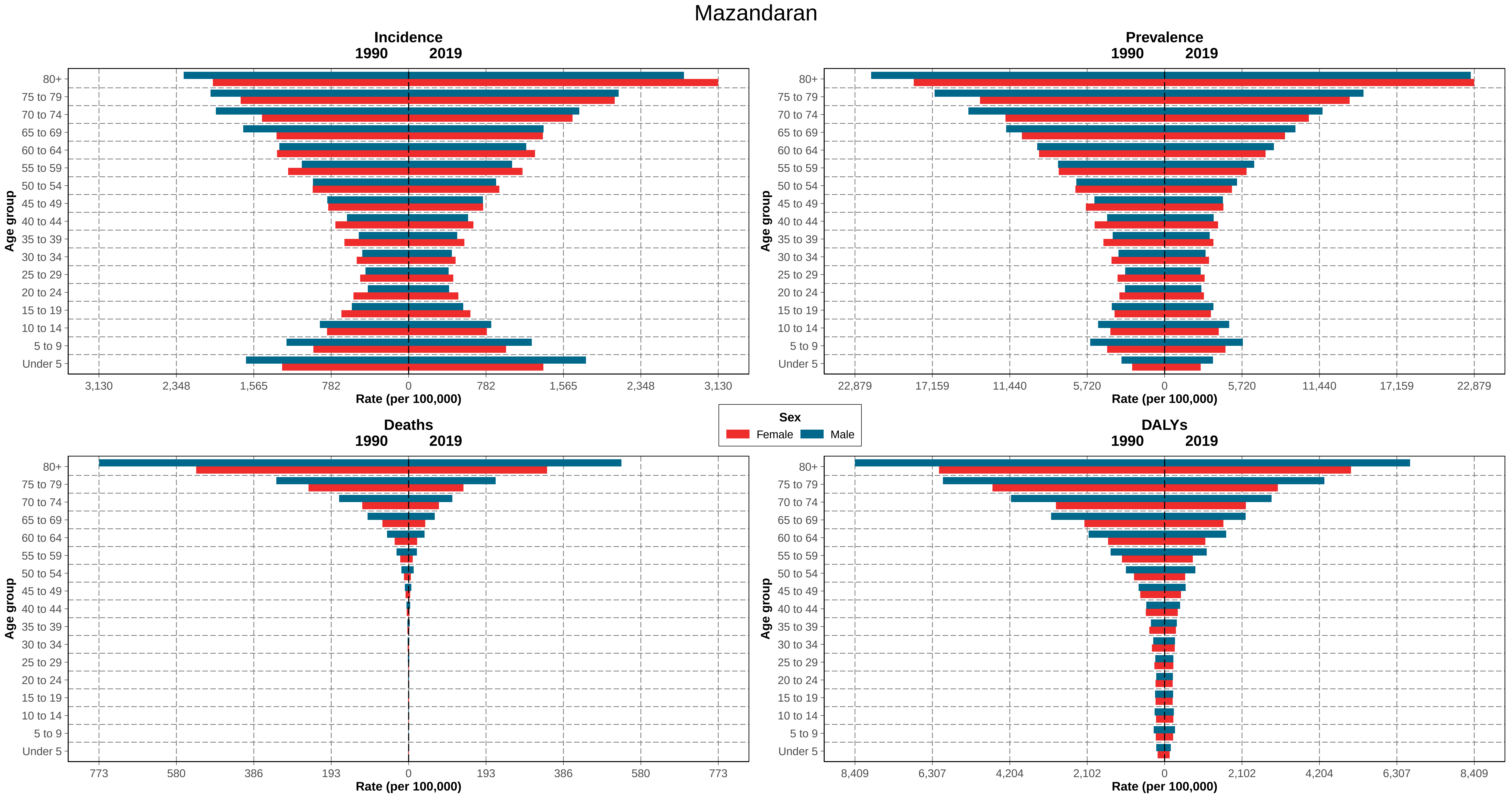
Mazandaran
Incidence
1990
2019
Prevalence
1990
2019
Deaths
1990
2019
DALYs
1990
2019
Age group
80+
75 to 79
70 to 74
65 to 69
60 to 64
55 to 59
50 to 54
45 to 49
40 to 44
35 to 39
30 to 34
25 to 29
20 to 24
15 to 19
10 to 14
5 to 9
Under 5
3,130 2,348 1,565 782 0 782 1,565 2,348 3,130
22,879 17,159 11,440 5,720 0 5,720 11,440 17,159 22,879
773 580 386 193 0 193 386 580 773
8,409 6,307 4,204 2,102 0 2,102 4,204 6,307 8,409
Rate (per 100,000)
Sex
Female
Male

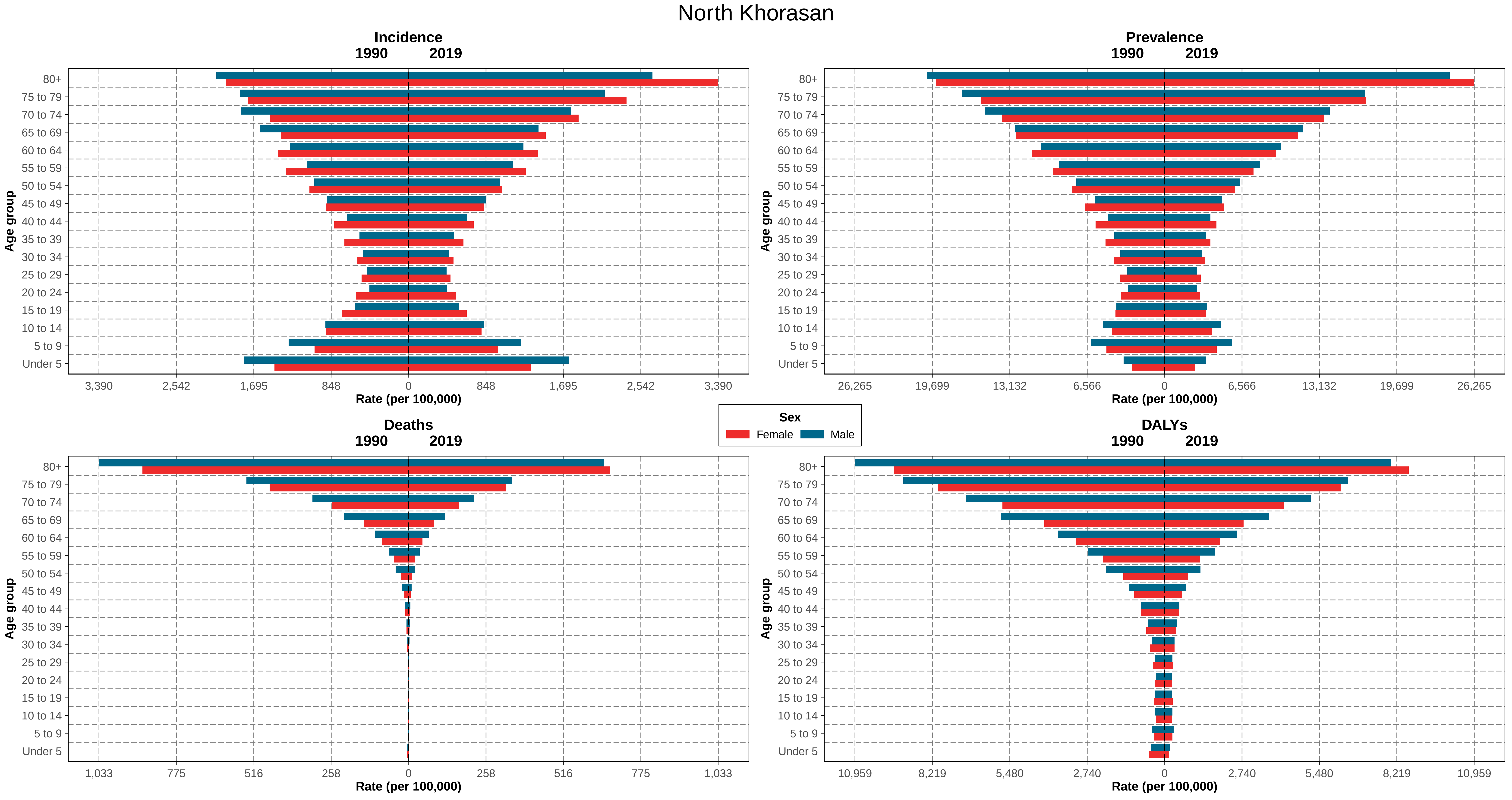
North Khorasan
Incidence
1990 2019
Prevalence
1990 2019
Deaths
1990 2019
DALYs
1990 2019
Age group
80+
75 to 79
70 to 74
65 to 69
60 to 64
55 to 59
50 to 54
45 to 49
40 to 44
35 to 39
30 to 34
25 to 29
20 to 24
15 to 19
10 to 14
5 to 9
Under 5
3,390 2,542 1,695 848 0 848 1,695 2,542 3,390
26,265 19,699 13,132 6,566 0 6,566 13,132 19,699 26,265
1,033 775 516 258 0 258 516 775 1,033
10,959 8,219 5,480 2,740 0 2,740 5,480 8,219 10,959
Rate (per 100,000)
Sex
Female
Male

Qazvin

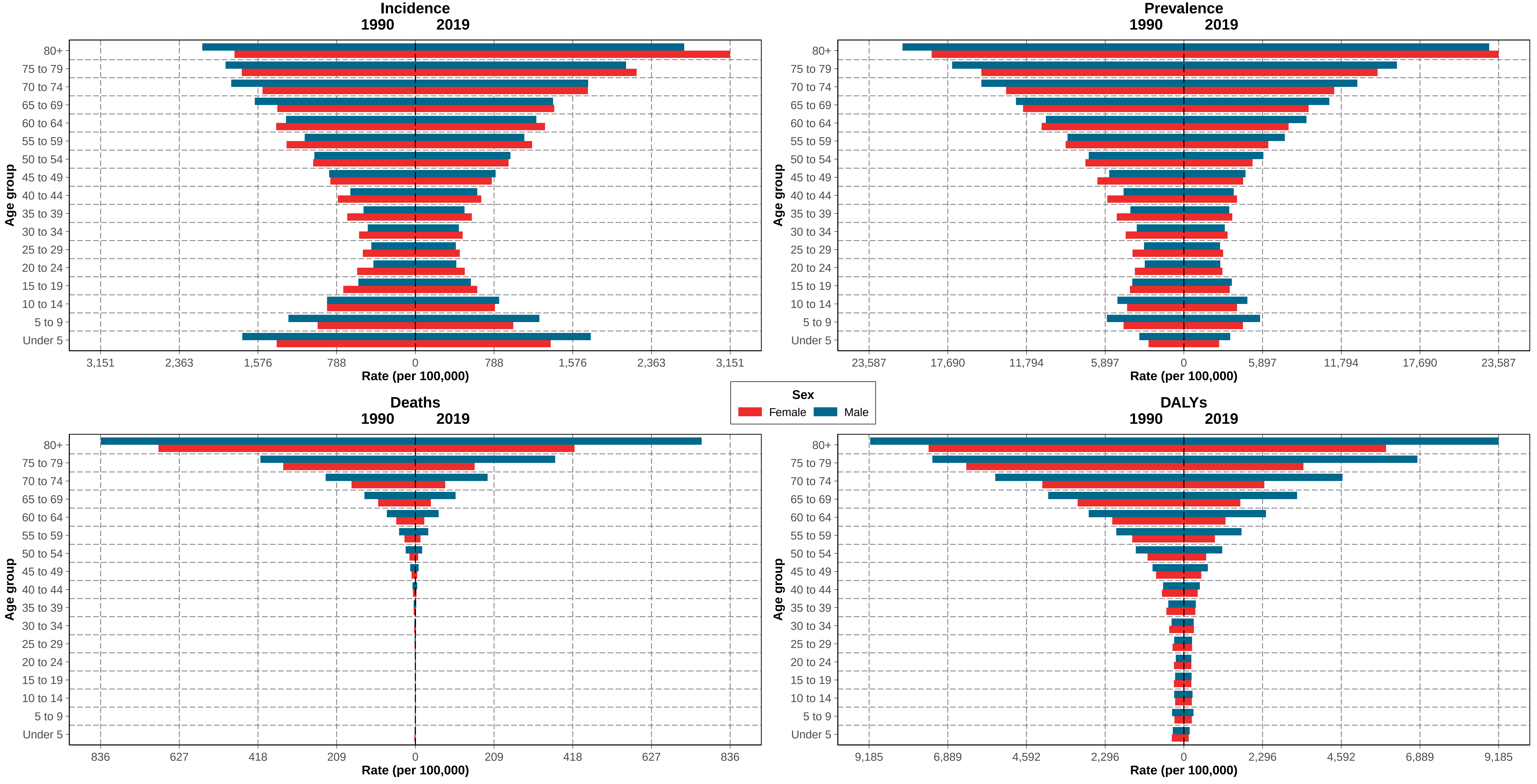

Incidence
1990 2019
Prevalence
1990 2019
Deaths
1990 2019
DALYs
1990 2019
Age group
80+
75 to 79
70 to 74
65 to 69
60 to 64
55 to 59
50 to 54
45 to 49
40 to 44
35 to 39
30 to 34
25 to 29
20 to 24
15 to 19
10 to 14
5 to 9
Under 5
3,151 2,363 1,576 788 0 788 1,576 2,363 3,151
23,587 17,690 11,794 5,897 0 5,897 11,794 17,690 23,587
836 627 418 209 0 209 418 627 836
9,185 6,889 4,592 2,296 0 2,296 4,592 6,889 9,185
Rate (per 100,000)
Sex
Female
Male

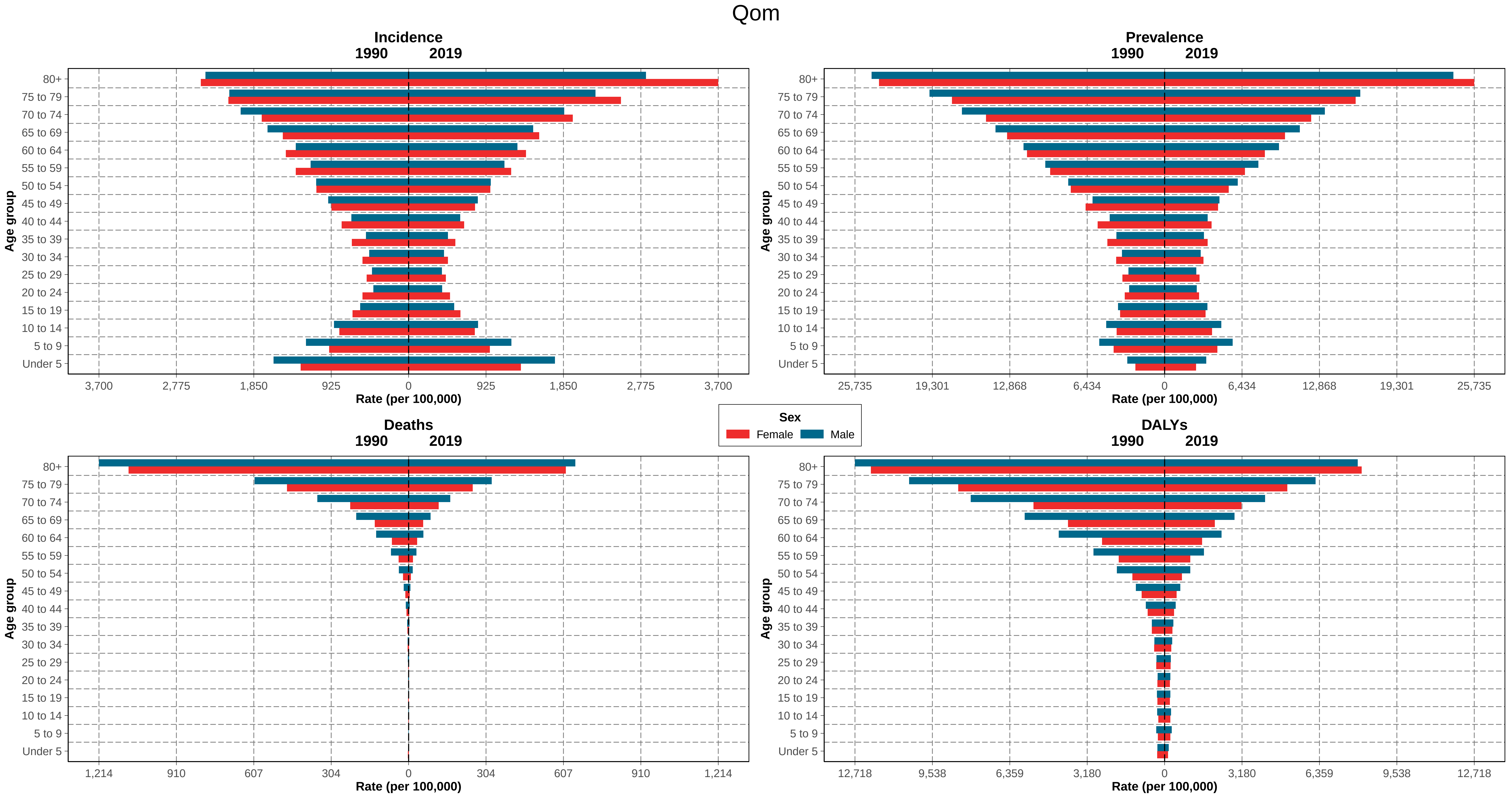

Qom
Incidence
1990 2019
Prevalence
1990 2019
Deaths
1990 2019
DALYs
1990 2019
Age group
80+
75 to 79
70 to 74
65 to 69
60 to 64
55 to 59
50 to 54
45 to 49
40 to 44
35 to 39
30 to 34
25 to 29
20 to 24
15 to 19
10 to 14
5 to 9
Under 5
3,700 2,775 1,850 925 0 925 1,850 2,775 3,700
25,735 19,301 12,868 6,434 0 6,434 12,868 19,301 25,735
1,214 910 607 304 0 304 607 910 1,214
12,718 9,538 6,359 3,180 0 3,180 6,359 9,538 12,718
Rate (per 100,000)
Sex
Female
Male

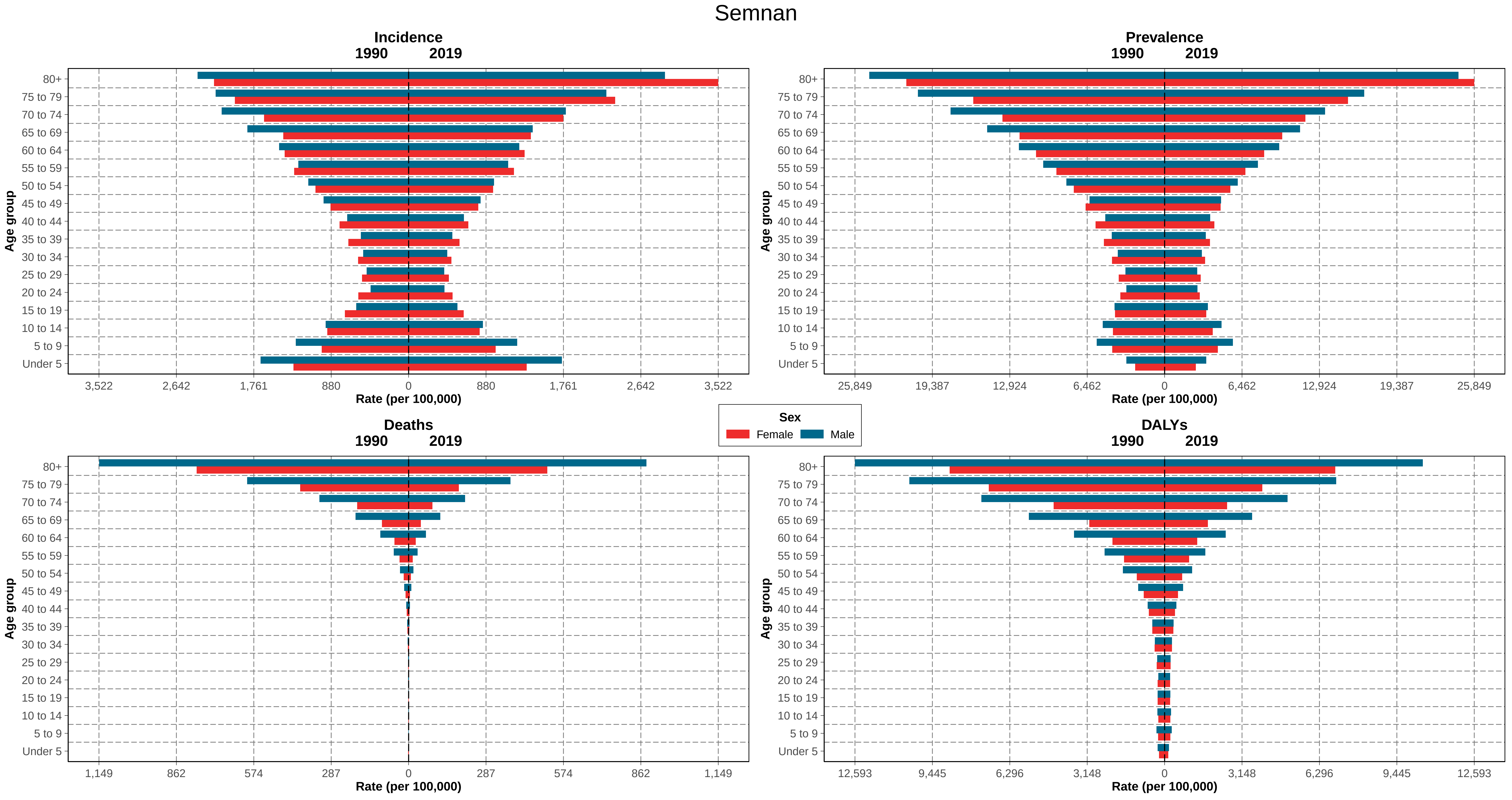
Semnan
Incidence
1990
2019
Prevalence
1990
2019
Deaths
1990
2019
DALYs
1990
2019
Age group
80+
75 to 79
70 to 74
65 to 69
60 to 64
55 to 59
50 to 54
45 to 49
40 to 44
35 to 39
30 to 34
25 to 29
20 to 24
15 to 19
10 to 14
5 to 9
Under 5
3,522 2,642 1,761 880 0 880 1,761 2,642 3,522
25,849 19,387 12,924 6,462 0 6,462 12,924 19,387 25,849
1,149 862 574 287 0 287 574 862 1,149
12,593 9,445 6,296 3,148 0 3,148 6,296 9,445 12,593
Rate (per 100,000)
Sex
Female
Male

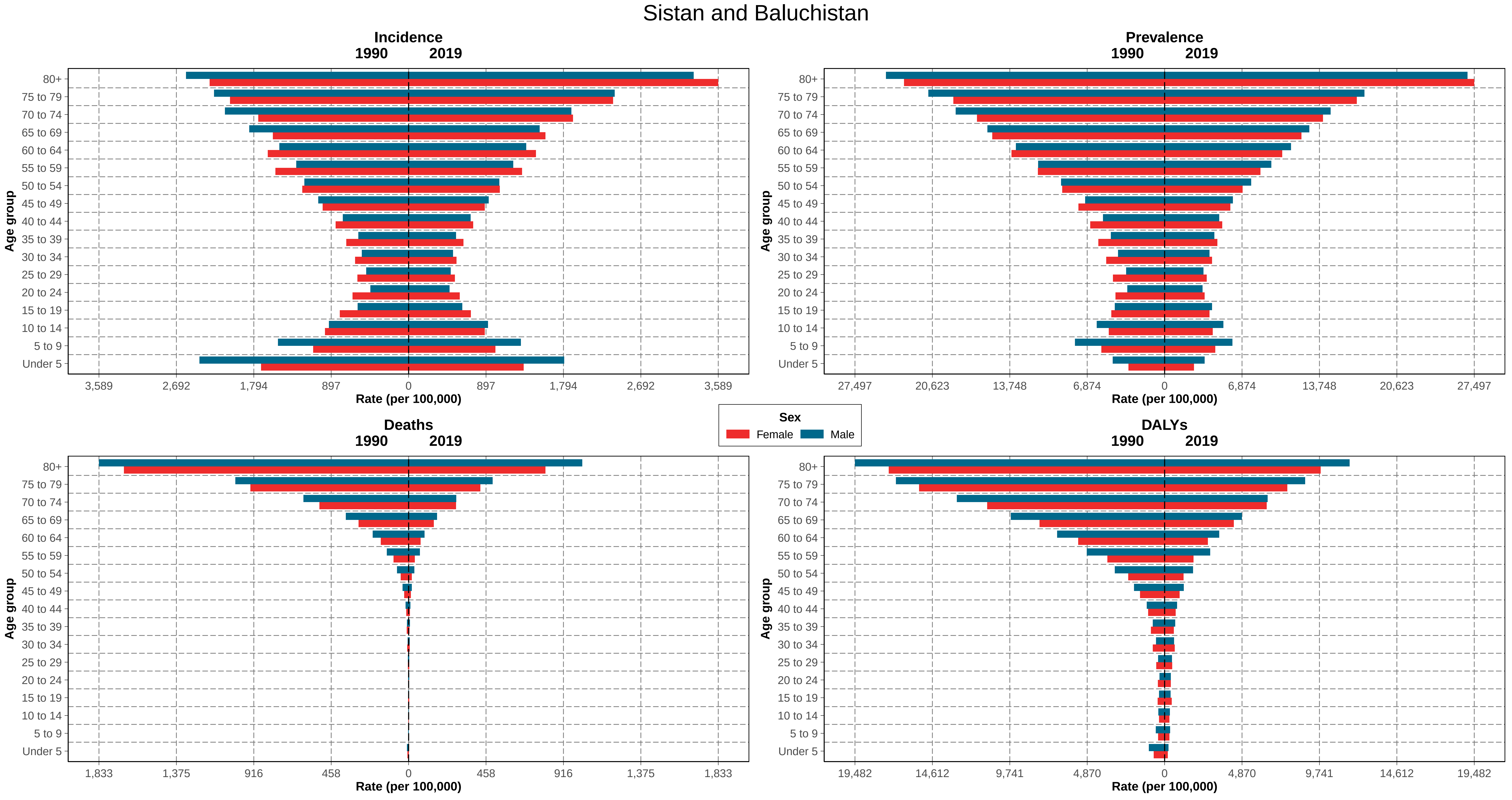

Sistan and Baluchistan
Incidence
1990
2019
Prevalence
Deaths
DALYs
Age group
80+
75 to 79
70 to 74
65 to 69
60 to 64
55 to 59
50 to 54
45 to 49
40 to 44
35 to 39
30 to 34
25 to 29
20 to 24
15 to 19
10 to 14
5 to 9
Under 5
3,589
2,692
1,794
897
0
27,497
20,623
13,748
6,874
1,833
1,375
916
458
19,482
14,612
9,741
4,870
Rate (per 100,000)
Sex
Female
Male

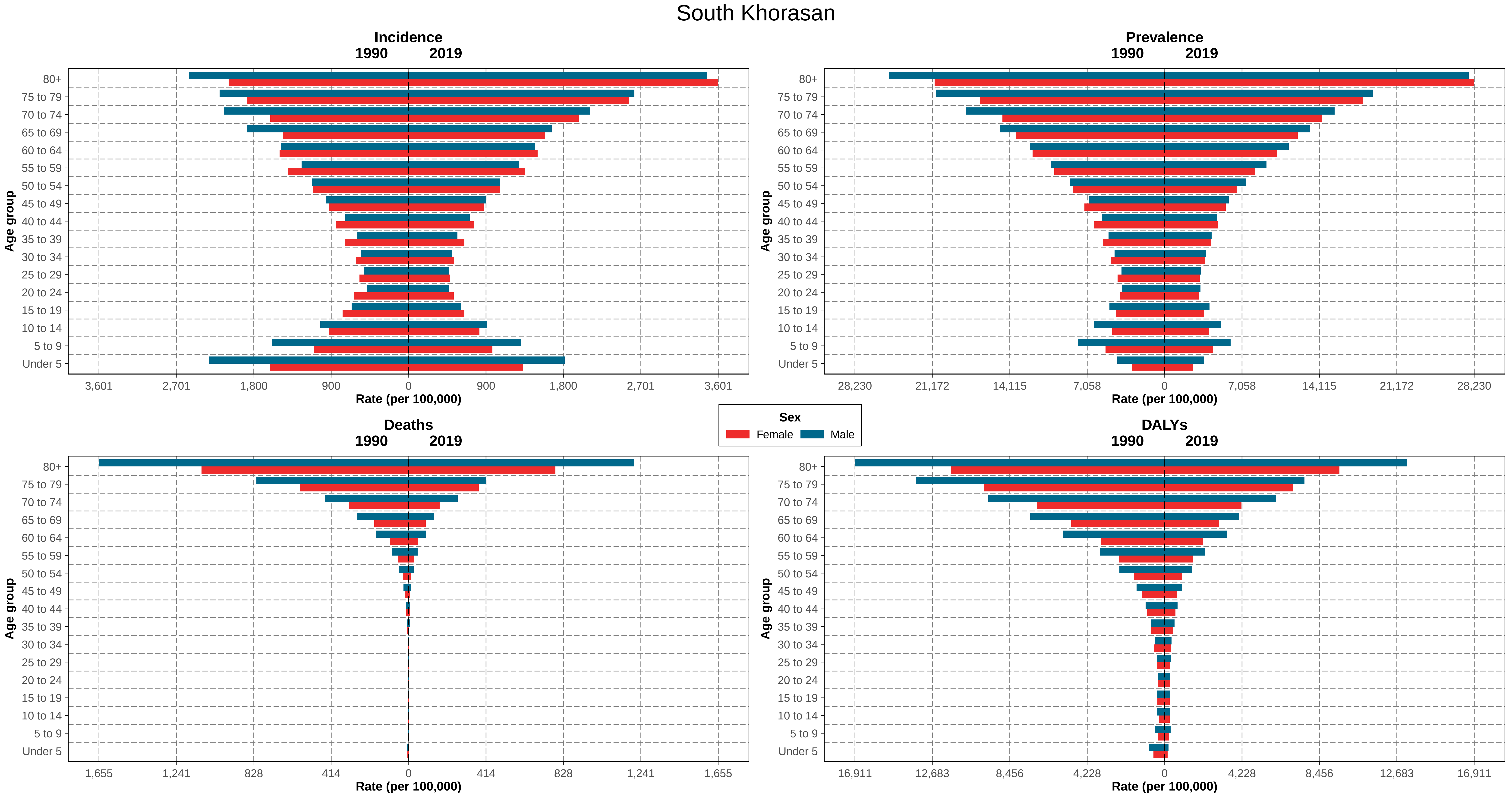
South Khorasan
Incidence
1990
2019
Prevalence
1990
2019
Deaths
1990
2019
DALYs
1990
2019
Age group
80+
75 to 79
70 to 74
65 to 69
60 to 64
55 to 59
50 to 54
45 to 49
40 to 44
35 to 39
30 to 34
25 to 29
20 to 24
15 to 19
10 to 14
5 to 9
Under 5
Rate (per 100,000)
3,601
2,701
1,800
900
0
900
1,800
2,701
3,601
28,230
21,172
14,115
7,058
0
7,058
14,115
21,172
28,230
1,655
1,241
828
414
0
414
828
1,241
1,655
16,911
12,683
8,456
4,228
0
4,228
8,456
12,683
16,911
Sex
Female
Male

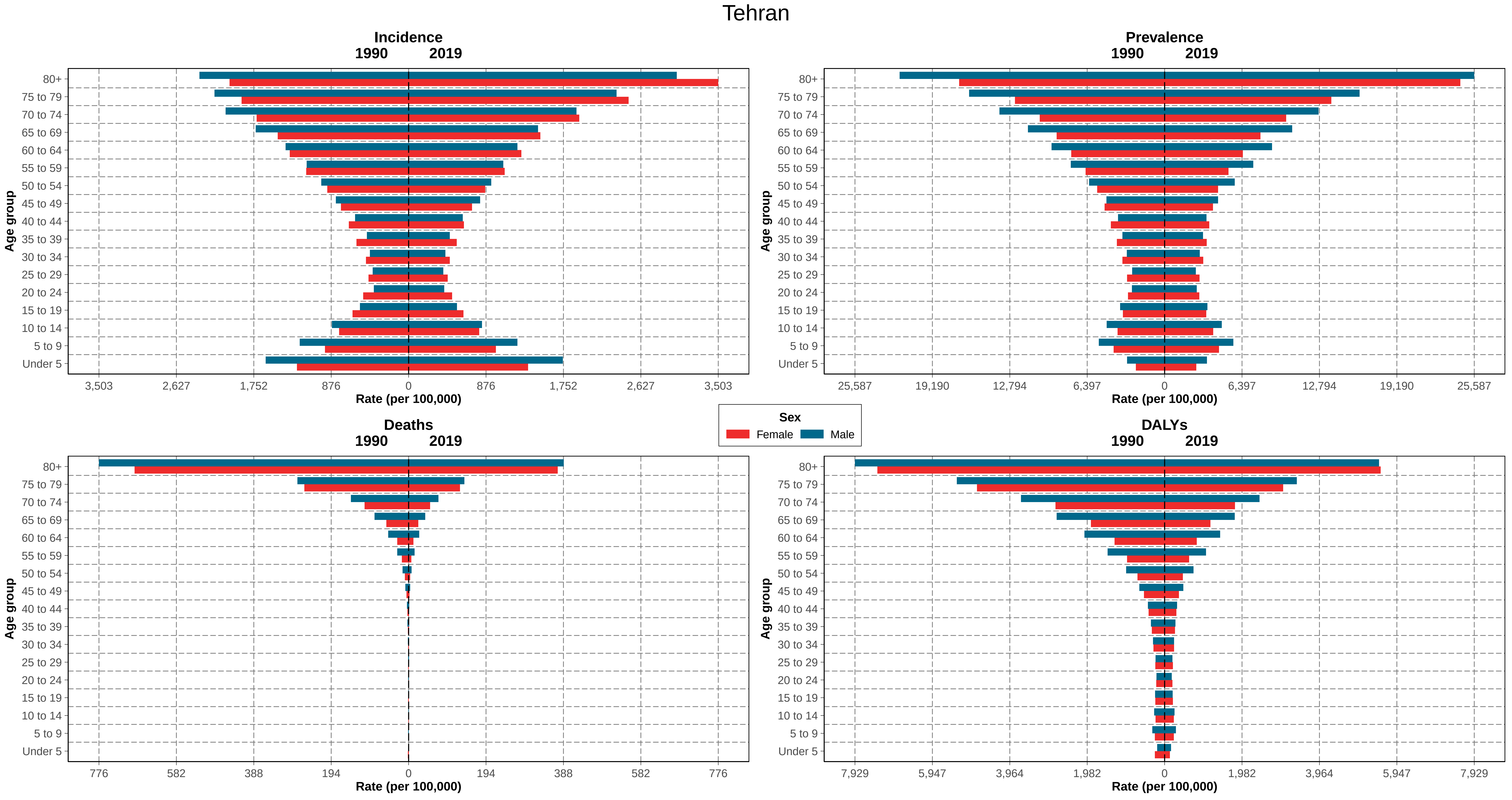
Tehran
Incidence
1990
2019
Prevalence
1990
2019
Deaths
1990
2019
DALYs
1990
2019
Age group
80+
75 to 79
70 to 74
65 to 69
60 to 64
55 to 59
50 to 54
45 to 49
40 to 44
35 to 39
30 to 34
25 to 29
20 to 24
15 to 19
10 to 14
5 to 9
Under 5
3,503
2,627
1,752
876
0
876
1,752
2,627
3,503
25,587
19,190
12,794
6,397
0
6,397
12,794
19,190
25,587
776
582
388
194
0
194
388
582
776
7,929
5,947
3,964
1,982
0
1,982
3,964
5,947
7,929
Rate (per 100,000)
Sex
Female
Male

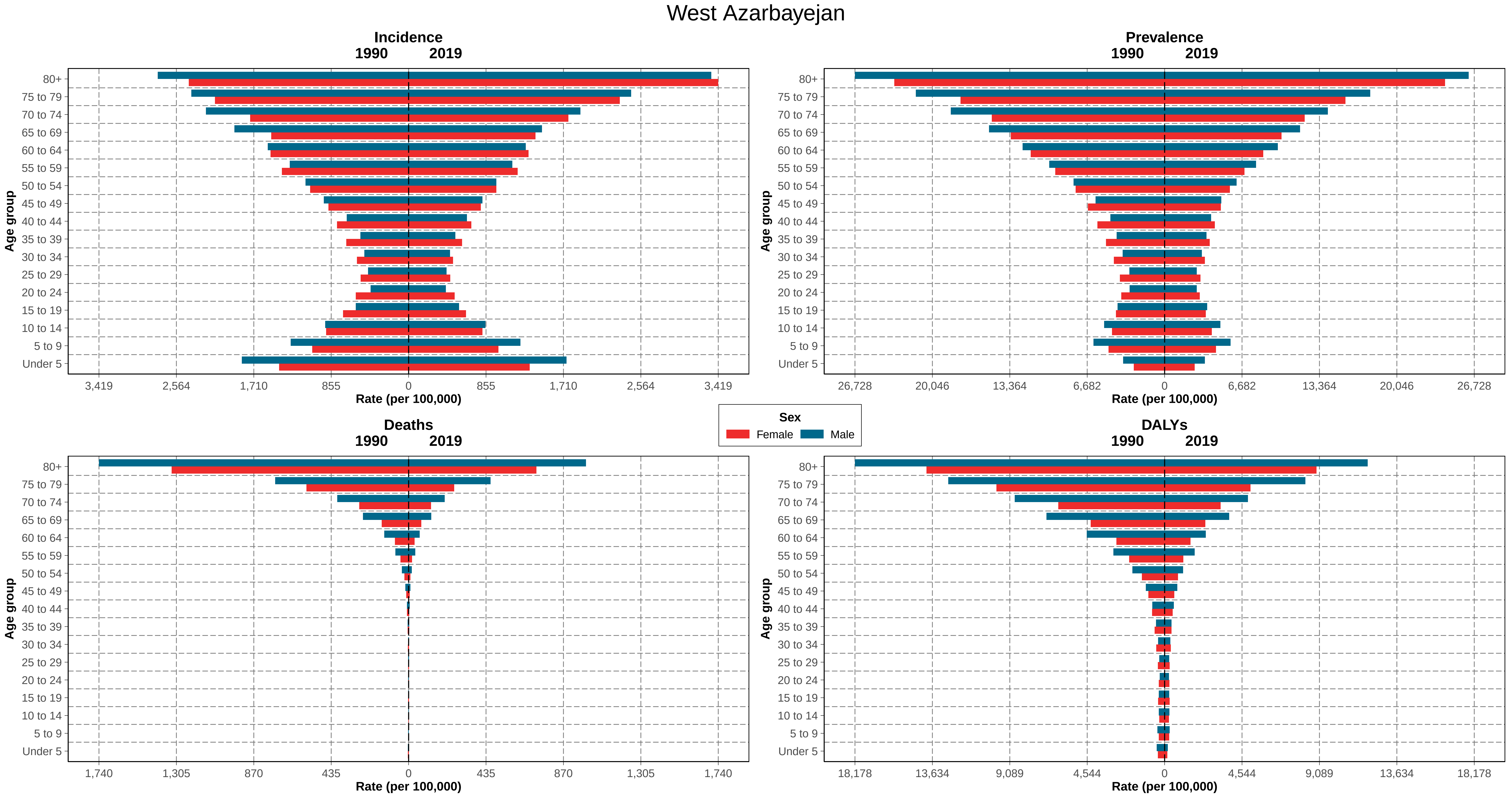
West Azarbayejan
Incidence
1990
2019
Prevalence
1990
2019
Deaths
1990
2019
DALYs
1990
2019
Age group
80+
75 to 79
70 to 74
65 to 69
60 to 64
55 to 59
50 to 54
45 to 49
40 to 44
35 to 39
30 to 34
25 to 29
20 to 24
15 to 19
10 to 14
5 to 9
Under 5
3,419
2,564
1,710
855
0
855
1,710
2,564
3,419
26,728
20,046
13,364
6,682
0
6,682
13,364
20,046
26,728
1,740
1,305
870
435
0
435
870
1,305
1,740
18,178
13,634
9,089
4,544
0
4,544
9,089
13,634
18,178
Rate (per 100,000)
Sex
Female
Male

Yazd

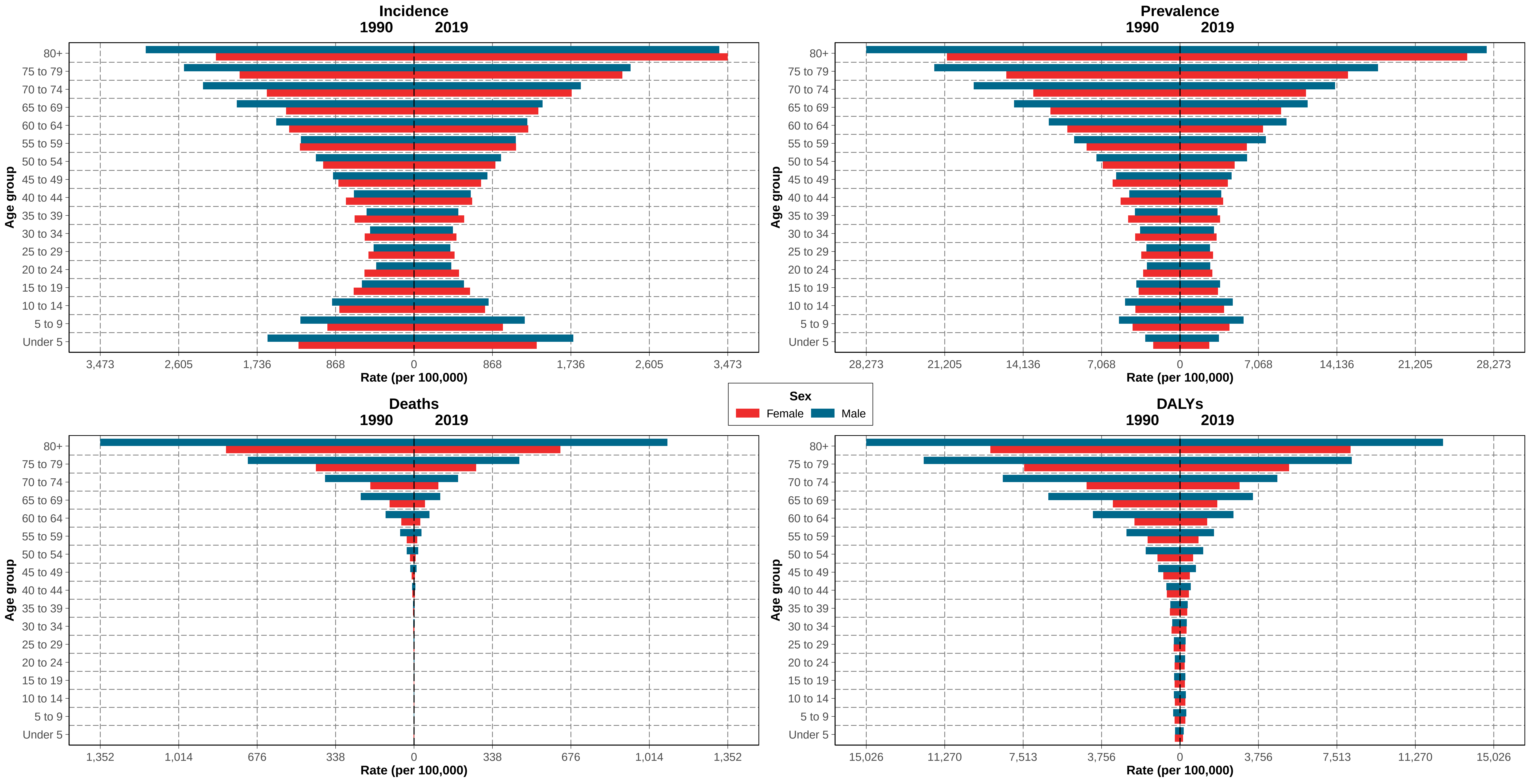

Incidence
1990
2019
Prevalence
Deaths
DALYs
Age group
80+
75 to 79
70 to 74
65 to 69
60 to 64
55 to 59
50 to 54
45 to 49
40 to 44
35 to 39
30 to 34
25 to 29
20 to 24
15 to 19
10 to 14
5 to 9
Under 5
3,473
2,605
1,736
868
0
28,273
21,205
14,136
7,068
1,352
1,014
676
338
15,026
11,270
7,513
3,756
Rate (per 100,000)
Sex
Female
Male

Zanjan

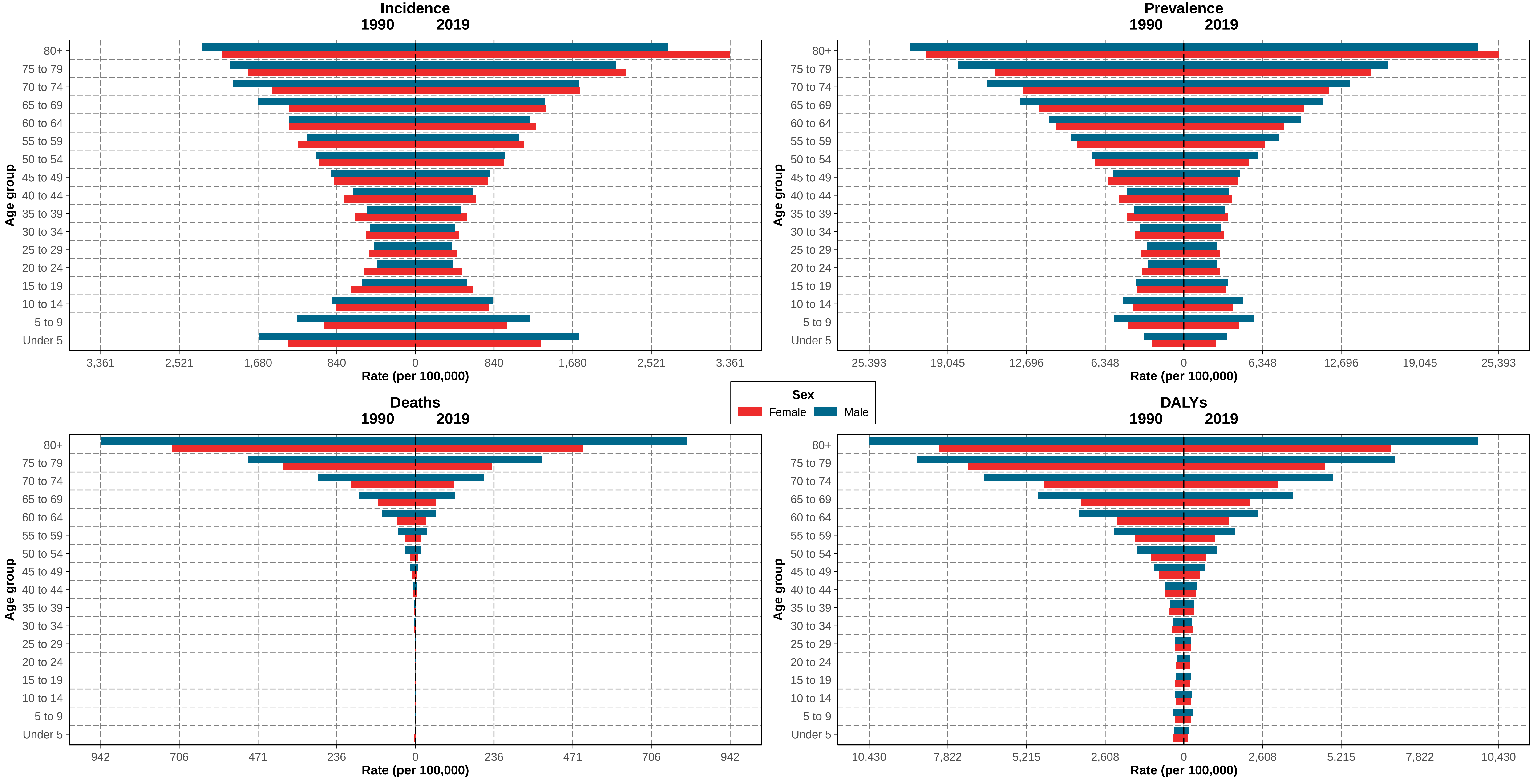

Incidence
1990
2019
Prevalence
1990
2019
Deaths
1990
2019
DALYs
1990
2019
Age group
80+
75 to 79
70 to 74
65 to 69
60 to 64
55 to 59
50 to 54
45 to 49
40 to 44
35 to 39
30 to 34
25 to 29
20 to 24
15 to 19
10 to 14
5 to 9
Under 5
3,361
2,521
1,680
840
0
840
1,680
2,521
3,361
25,393
19,045
12,696
6,348
0
6,348
12,696
19,045
25,393
942
706
471
236
0
236
471
706
942
10,430
7,822
5,215
2,608
0
2,608
5,215
7,822
10,430
Rate (per 100,000)
Sex
Female
Male
