# Supplementary material for: National, subnational and risk attributed burden of chronic respiratory diseases in Iran from 1990 to 2019
Source: Respir Res. 2023 Mar 11;24:74. doi: 10.1186/s12931-023-02353-1 (PMC10006557; doi:10.1186/s12931-023-02353-1)
Supplement: Supplementary file 5 — Additional file 5: Table S1. Burden measures of CRDs for all ages number and ASR with percentage change by sex at national level, 1990 vs 2019. Data in parentheses are 95% Uncertainty Intervals (95% UIs); DALYs= Disability-Adjusted Life Years; YLLs= Years of Life Lost; YLDs= Years Lived with Disability [file 12931_2023_2353_MOESM5_ESM.pdf]

Cause	Measure	Age (metric)	Year						% Change (1990 to 2019)		
			1990			2019					
			Both	Female	Male	Both	Female	Male	Both	Female	Male
Chronic obstructive pulmonary disease	Incidence	All ages (number)	32,052 (29,255 to 35,372)	14,421 (13,206 to 15,780)	17,630 (15,955 to 19,602)	103,602 (94,771 to 113,265)	45,891 (41,883 to 50,306)	57,711 (52,839 to 62,967)	223.2 (202.7 to 244.8)	218.2 (198.1 to 239.5)	227.3 (206.4 to 250.3)
		Age-standardized (rate per 100,000)	99.2 (89.5 to 109.8)	90.9 (82 to 100.8)	106.9 (96.4 to 118.3)	140.6 (127.8 to 154.5)	129.8 (117.8 to 142.9)	151.7 (137.9 to 166.6)	41.7 (35 to 47.5)	42.8 (35.4 to 49.9)	41.9 (35.2 to 47.9)
	Prevalence	All ages (number)	509,008 (465,525 to 555,495)	232,079 (214,369 to 251,505)	276,928 (251,490 to 304,832)	1,562,415 (1,446,402 to 1,688,536)	686,719 (633,599 to 744,997)	875,696 (806,519 to 949,356)	207 (194.3 to 222)	195.9 (183.9 to 210.2)	216.2 (200.9 to 233.4)
		Age-standardized (rate per 100,000)	1640 (1508.2 to 1794.9)	1520.6 (1396.7 to 1661.9)	1751.8 (1600.2 to 1933.5)	2055.5 (1891.4 to 2232.6)	1832.2 (1687.7 to 1989.4)	2282.8 (2096.2 to 2487.5)	25.3 (20.9 to 29.8)	20.5 (15.8 to 25.3)	30.3 (25.4 to 35.3)
	Deaths	All ages (number)	4,230 (3,709 to 5,228)	1,641 (1,075 to 2,305)	2,588 (2,216 to 3,296)	12,557 (10,993 to 13,621)	4,855 (3,932 to 5,781)	7,701 (6,760 to 8,327)	196.9 (139.3 to 246.1)	195.9 (111.8 to 437.2)	197.5 (126 to 253.6)
		Age-standardized (rate per 100,000)	23.4 (20.1 to 29.4)	19 (12.8 to 27.4)	28 (23.5 to 35.3)	20.3 (17.7 to 22.1)	16.4 (12.9 to 19.6)	24.2 (21.3 to 26.3)	-13 (-30.5 to 1.7)	-13.9 (-38.3 to 49.6)	-13.4 (-32.9 to 2.9)
	DALYs	All ages (number)	153,371 (138,121 to 176,277)	63,198 (49,406 to 77,006)	90,173 (79,421 to 109,525)	372,569 (338,458 to 404,911)	151,368 (131,933 to 170,196)	221,200 (199,050 to 240,391)	142.9 (109 to 168)	139.5 (101.2 to 250.6)	145.3 (97.6 to 179.1)
		Age-standardized (rate per 100,000)	555.9 (495 to 646.6)	466.8 (365.9 to 593)	641.6 (565.9 to 776.5)	517.2 (471 to 560.8)	427.5 (371.6 to 480.7)	607.8 (549.4 to 662)	-7 (-20.6 to 3.2)	-8.4 (-26.5 to 34.4)	-5.3 (-23.3 to 7.8)
	YLLs	All ages (number)	105,098 (93,069 to 126,741)	39,544 (24,045 to 53,086)	65,554 (56,326 to 84,296)	227,958 (205,149 to 247,566)	83,425 (68,878 to 100,095)	144,533 (128,093 to 156,202)	116.9 (74.4 to 150.3)	111 (59.3 to 313.1)	120.5 (64 to 163.8)
		Age-standardized (rate per 100,000)	404.4 (353.9 to 496.7)	317 (206.1 to 445)	489 (418.4 to 621.4)	329.1 (296.7 to 356.4)	248.8 (203.9 to 296.9)	409.7 (364.4 to 441.8)	-18.6 (-33.8 to -5.9)	-21.5 (-41.8 to 44.9)	-16.2 (-36.4 to -0.3)
	YLDs	All ages (number)	48,273 (39,156 to 56,242)	23,654 (19,298 to 27,733)	24,619 (19,609 to 29,209)	144,611 (117,257 to 168,236)	67,944 (55,568 to 79,514)	76,667 (60,745 to 91,234)	199.6 (186.6 to 214.4)	187.2 (173.9 to 202.6)	211.4 (195.6 to 230.1)
		Age-standardized (rate per 100,000)	151.5 (123.3 to 175.9)	149.9 (121.9 to 175)	152.6 (121.6 to 181.5)	188.1 (152.9 to 218.4)	178.7 (145 to 208.3)	198.1 (157.2 to 235.4)	24.2 (19.9 to 28.6)	19.2 (14.3 to 24.2)	29.8 (24.5 to 35.3)

Cause	Measure	Age (metric)	Year						% Change (1990 to 2019)		
			1990			2019					
			Both	Female	Male	Both	Female	Male	Both	Female	Male
Pneumoconiosis	Incidence	All ages (number)	290 (218 to 370)	262 (194 to 340)	28 (21 to 37)	2,027 (1,562 to 2,584)	1,949 (1,486 to 2,497)	78 (64 to 95)	600.1 (508.8 to 716.8)	645.4 (541.4 to 781.9)	177.8 (120.2 to 250.5)
		Age-standardized (rate per 100,000)	0.8 (0.6 to 0.9)	1.3 (1 to 1.7)	0.2 (0.1 to 0.2)	2.4 (1.9 to 3)	4.6 (3.5 to 5.7)	0.2 (0.2 to 0.3)	212.8 (178.5 to 251.2)	238.6 (201.3 to 282.3)	20.9 (0.2 to 45.3)
	Prevalence	All ages (number)	575 (457 to 711)	459 (352 to 583)	117 (97 to 141)	5,749 (4,497 to 7,201)	5,152 (3,866 to 6,583)	597 (489 to 722)	899.3 (773 to 1025.5)	1023.6 (866.4 to 1189.8)	411.2 (339.4 to 501)
		Age-standardized (rate per 100,000)	1.7 (1.5 to 2.1)	2.5 (2 to 3.2)	1 (0.8 to 1.2)	6.9 (5.6 to 8.5)	12.1 (9.4 to 15.2)	1.8 (1.5 to 2.2)	296 (254.6 to 335.7)	376.4 (327.5 to 429.6)	83 (60.6 to 111.5)
	Deaths	All ages (number)	12 (9 to 16)	2 (1 to 3)	10 (7 to 13)	68 (59 to 77)	17 (13 to 20)	51 (43 to 59)	452 (310.1 to 725)	631.7 (392.4 to 1106.6)	410.8 (256.3 to 774.4)
		Age-standardized (rate per 100,000)	0.1 (0 to 0.1)	0 (0 to 0)	0.1 (0.1 to 0.1)	0.1 (0.1 to 0.1)	0.1 (0 to 0.1)	0.2 (0.1 to 0.2)	89.6 (41.9 to 174.8)	128.3 (46.9 to 265.4)	77.6 (24.5 to 207.6)
	DALYs	All ages (number)	443 (342 to 546)	141 (104 to 189)	303 (208 to 400)	2,422 (2,010 to 2,890)	1,176 (875 to 1,575)	1,246 (1,056 to 1,429)	446.6 (320.8 to 635.9)	736.4 (551.6 to 970.1)	311.9 (189.8 to 556.4)
		Age-standardized (rate per 100,000)	1.4 (1.1 to 1.8)	0.9 (0.6 to 1.1)	2 (1.4 to 2.6)	3.1 (2.6 to 3.7)	2.9 (2.2 to 3.8)	3.4 (2.9 to 3.9)	117.3 (68.1 to 191.5)	237.9 (166.8 to 331.6)	69.8 (21.3 to 171)
	YLLs	All ages (number)	352 (255 to 450)	68 (43 to 98)	284 (190 to 379)	1,534 (1,329 to 1,743)	383 (310 to 480)	1,151 (972 to 1,327)	335.8 (217.3 to 547.3)	460.3 (258.9 to 886.6)	305.8 (178.2 to 573.7)
		Age-standardized (rate per 100,000)	1.2 (0.9 to 1.5)	0.5 (0.3 to 0.6)	1.8 (1.2 to 2.4)	2.1 (1.8 to 2.3)	1 (0.8 to 1.3)	3.1 (2.6 to 3.5)	77.6 (31.9 to 162.6)	128.8 (52.4 to 278.2)	68.9 (16.6 to 183.9)
	YLDs	All ages (number)	91 (58 to 134)	72 (45 to 109)	19 (12 to 28)	888 (570 to 1,316)	793 (502 to 1,191)	95 (61 to 140)	874.9 (752.9 to 1000.5)	997.7 (846.4 to 1160.2)	404.7 (335.1 to 489.2)
		Age-standardized (rate per 100,000)	0.3 (0.2 to 0.4)	0.4 (0.3 to 0.6)	0.2 (0.1 to 0.2)	1.1 (0.7 to 1.6)	1.9 (1.2 to 2.7)	0.3 (0.2 to 0.4)	283 (242.6 to 321.7)	361.2 (310.3 to 412.1)	80.6 (58.7 to 107.9)

Cause	Measure	Age (metric)	Year						% Change (1990 to 2019)		
			1990			2019					
			Both	Female	Male	Both	Female	Male	Both	Female	Male
Asthma	Incidence	All ages (number)	433,579 (323,087 to 565,952)	200,852 (153,485 to 259,953)	232,726 (171,302 to 306,562)	415,328 (327,426 to 524,205)	199,261 (160,050 to 247,080)	216,066 (167,076 to 276,398)	-4.2 (-9.7 to 3.2)	-0.8 (-7.4 to 7.5)	-7.2 (-12.3 to -0.4)
		Age-standardized (rate per 100,000)	646 (516.2 to 800.1)	621.8 (507.1 to 763.1)	668.8 (527.5 to 832.9)	544.1 (418.9 to 689.9)	528.9 (417.9 to 665.2)	558 (425.8 to 722.2)	-15.8 (-20.4 to -10.9)	-14.9 (-19 to -10.5)	-16.6 (-21.8 to -11.5)
	Prevalence	All ages (number)	2,298,639 (1,871,634 to 2,861,152)	1,082,051 (890,068 to 1,328,073)	1,216,588 (980,821 to 1,535,748)	2,635,573 (2,182,430 to 3,205,594)	1,293,635 (1,078,608 to 1,565,478)	1,341,938 (1,104,357 to 1,640,201)	14.7 (7.9 to 24.5)	19.6 (11.8 to 30.1)	10.3 (3.4 to 20.1)
		Age-standardized (rate per 100,000)	4235.2 (3602.4 to 5012)	4179.4 (3551.6 to 4922.6)	4296.4 (3634.8 to 5062.5)	3280.1 (2717.3 to 3978.4)	3241.6 (2687 to 3922.7)	3313.4 (2693.4 to 4042.2)	-22.6 (-26.7 to -18)	-22.4 (-26.5 to -18.1)	-22.9 (-27.4 to -17.6)
	Deaths	All ages (number)	3,806 (3,075 to 4,777)	1,646 (1,112 to 2,374)	2,160 (1,755 to 2,738)	3,606 (3,095 to 4,030)	1,645 (1,209 to 1,935)	1,961 (1,728 to 2,212)	-5.3 (-29.6 to 21.9)	-0.1 (-36.7 to 37.1)	-9.2 (-32 to 22)
		Age-standardized (rate per 100,000)	18.5 (14 to 24.9)	16.5 (9.8 to 26.1)	20.6 (16.5 to 27.8)	5.6 (4.8 to 6.2)	5.2 (3.8 to 6.1)	6 (5.3 to 6.8)	-69.7 (-78.9 to -59.7)	-68.2 (-81.8 to -54.6)	-70.9 (-79.7 to -60.8)
	DALYs	All ages (number)	200,080 (159,763 to 248,232)	90,398 (69,201 to 116,418)	109,682 (89,124 to 136,252)	178,733 (141,139 to 230,621)	84,388 (64,903 to 108,840)	94,344 (74,917 to 121,556)	-10.7 (-21.8 to 0.7)	-6.6 (-22.3 to 8.2)	-14 (-25.1 to -0.2)
		Age-standardized (rate per 100,000)	529.3 (431 to 652)	485.3 (363.6 to 654.1)	571 (477.6 to 690.7)	232.3 (185 to 299.3)	220.8 (169.9 to 284.9)	243.7 (194.8 to 311.8)	-56.1 (-64.9 to -47.5)	-54.5 (-67.1 to -42.2)	-57.3 (-65.1 to -47.4)
	YLLs	All ages (number)	109,371 (92,951 to 129,893)	47,897 (35,730 to 61,278)	61,474 (50,716 to 74,274)	75,647 (67,236 to 86,659)	34,157 (27,060 to 41,672)	41,489 (36,911 to 47,149)	-30.8 (-44 to -11.5)	-28.7 (-49.5 to -6.2)	-32.5 (-45.3 to -8.5)
		Age-standardized (rate per 100,000)	365.4 (294.6 to 457.3)	324.3 (218.8 to 464.8)	404 (328.6 to 509.6)	104.1 (92.1 to 118.4)	95 (74 to 114.7)	113.4 (101.8 to 128.2)	-71.5 (-78.9 to -62.6)	-70.7 (-81.4 to -59.1)	-71.9 (-78.5 to -62)
	YLDs	All ages (number)	90,710 (57,164 to 136,079)	42,502 (27,084 to 63,483)	48,208 (30,134 to 72,215)	103,086 (66,253 to 155,079)	50,231 (32,406 to 74,956)	52,855 (33,835 to 79,889)	13.6 (6.8 to 23.9)	18.2 (10.4 to 28.9)	9.6 (2.8 to 19.5)
		Age-standardized (rate per 100,000)	163.9 (106.8 to 240.2)	161 (104.1 to 236.5)	167 (107.6 to 245.3)	128.2 (82.4 to 190.9)	125.8 (81.3 to 187.5)	130.3 (82.6 to 195.5)	-21.8 (-26.1 to -17.1)	-21.9 (-26.3 to -17.2)	-22 (-26.7 to -16.6)

Cause	Measure	Age (metric)	Year						% Change (1990 to 2019)		
			1990			2019					
			Both	Female	Male	Both	Female	Male	Both	Female	Male
Interstitial lung disease and pulmonary sarcoidosis	Incidence	All ages (number)	79,556 (66,097 to 95,917)	39,609 (32,821 to 47,934)	39,947 (33,223 to 47,906)	210,274 (170,206 to 259,196)	107,918 (86,930 to 134,363)	102,356 (83,020 to 125,451)	164.3 (144.9 to 181.5)	172.5 (152.2 to 189.2)	156.2 (137.1 to 174.6)
		Age-standardized (rate per 100,000)	202.8 (163.6 to 249)	210.2 (168.7 to 260.8)	196.5 (159.9 to 239.2)	245 (198.5 to 298.1)	252 (203.6 to 309)	238 (193.6 to 287.5)	20.8 (18.4 to 22.9)	19.9 (17.5 to 22.5)	21.1 (18.6 to 23.6)
	Prevalence	All ages (number)	10,892 (8,945 to 13,142)	5,419 (4,418 to 6,569)	5,473 (4,520 to 6,579)	28,589 (23,241 to 35,098)	14,655 (11,915 to 18,088)	13,934 (11,337 to 17,072)	162.5 (142.3 to 179.4)	170.4 (150.4 to 187.4)	154.6 (134 to 172.5)
		Age-standardized (rate per 100,000)	27.8 (22.7 to 33.8)	28.7 (23.3 to 35.3)	26.9 (22.2 to 32.6)	33.2 (27.3 to 39.9)	34.1 (27.8 to 41.2)	32.3 (26.6 to 38.6)	19.5 (17.2 to 22)	18.6 (15.7 to 21.9)	19.9 (17.4 to 22.8)
	Deaths	All ages (number)	106 (78 to 137)	49 (29 to 72)	56 (39 to 77)	371 (233 to 441)	158 (84 to 199)	214 (114 to 263)	251.7 (139.8 to 389.6)	219.7 (124.2 to 481.2)	279.7 (118.1 to 480.5)
		Age-standardized (rate per 100,000)	0.5 (0.4 to 0.7)	0.5 (0.3 to 0.8)	0.6 (0.4 to 0.8)	0.6 (0.4 to 0.7)	0.5 (0.3 to 0.6)	0.6 (0.4 to 0.8)	5.2 (-26.8 to 52.6)	-7.6 (-37.7 to 75.6)	17.9 (-30 to 89.6)
	DALYs	All ages (number)	3,977 (3,121 to 4,908)	1,829 (1,277 to 2,418)	2,148 (1,621 to 2,839)	11,389 (7,941 to 13,899)	5,195 (3,486 to 6,506)	6,194 (3,665 to 7,717)	186.4 (113.7 to 262)	184.1 (124.6 to 288.8)	188.3 (75.7 to 288.3)
		Age-standardized (rate per 100,000)	13.1 (10.1 to 16.3)	12.7 (8.6 to 17.4)	13.6 (10.2 to 17.7)	14.7 (10.3 to 17.7)	13.4 (8.8 to 16.5)	16.1 (9.7 to 19.6)	12.1 (-15.4 to 46.1)	5.7 (-20.9 to 57.7)	18.4 (-24.5 to 66.1)
	YLLs	All ages (number)	2,767 (2,059 to 3,515)	1,232 (758 to 1,783)	1,535 (1,058 to 2,147)	8,296 (5,068 to 9,947)	3,623 (2,013 to 4,536)	4,672 (2,289 to 5,799)	199.8 (97.8 to 312.9)	194 (110.8 to 373.4)	204.4 (51.8 to 349)
		Age-standardized (rate per 100,000)	10.1 (7.4 to 13)	9.5 (5.6 to 14)	10.6 (7.4 to 14.5)	11.2 (6.9 to 13.4)	9.8 (5.3 to 12.2)	12.6 (6.4 to 15.5)	10.9 (-24.4 to 56)	2.4 (-29.2 to 84.6)	19 (-34.9 to 84.1)
	YLDs	All ages (number)	1,210 (777 to 1,801)	596 (383 to 888)	614 (392 to 922)	3,093 (2,004 to 4,687)	1,571 (1,014 to 2,361)	1,522 (995 to 2,311)	155.6 (135.7 to 175)	163.5 (140.2 to 187.2)	148 (126 to 168.5)
		Age-standardized (rate per 100,000)	3.1 (2 to 4.6)	3.1 (2 to 4.8)	3 (1.9 to 4.5)	3.6 (2.3 to 5.3)	3.6 (2.3 to 5.4)	3.5 (2.3 to 5.2)	16.1 (11.4 to 21.4)	15.6 (8.6 to 23.2)	16.3 (10.8 to 21.8)

Cause	Measure	Age (metric)	Year						% Change (1990 to 2019)		
			1990			2019					
			Both	Female	Male	Both	Female	Male	Both	Female	Male
Other chronic respiratory diseases	Incidence	All ages (number)	-	-	-	-	-	-	-	-	-
		Age-standardized (rate per 100,000)	-	-	-	-	-	-	-	-	-
	Prevalence	All ages (number)	-	-	-	-	-	-	-	-	-
		Age-standardized (rate per 100,000)	-	-	-	-	-	-	-	-	-
	Deaths	All ages (number)	53 (35 to 102)	23 (13 to 52)	30 (20 to 55)	233 (78 to 329)	89 (31 to 124)	144 (43 to 212)	338.6 (-8.3 to 775.1)	288 (-22.5 to 799.2)	376.8 (-1.6 to 887.9)
		Age-standardized (rate per 100,000)	0.2 (0.1 to 0.2)	0.1 (0.1 to 0.2)	0.2 (0.1 to 0.3)	0.3 (0.1 to 0.5)	0.3 (0.1 to 0.4)	0.4 (0.1 to 0.6)	100.5 (-40.8 to 249.1)	77.2 (-47.2 to 285.6)	116.4 (-40.5 to 323.9)
	DALYs	All ages (number)	7,557 (5,760 to 11,412)	3,822 (2,866 to 6,091)	3,735 (2,850 to 5,532)	22,800 (16,969 to 27,606)	10,822 (8,414 to 12,955)	11,978 (8,365 to 14,880)	201.7 (66.1 to 302.4)	183.1 (58.1 to 276.1)	220.7 (73.3 to 341.9)
		Age-standardized (rate per 100,000)	13.4 (10.8 to 18.2)	13.5 (10.7 to 19.2)	13.4 (10.5 to 18.1)	26.8 (19.6 to 32.5)	25.9 (19.7 to 31.1)	27.6 (18.9 to 34.6)	98.9 (21.4 to 156.2)	91.9 (19.2 to 149)	106.4 (22.5 to 175.9)
	YLLs	All ages (number)	2,693 (1,585 to 6,546)	1,177 (561 to 3,486)	1,516 (910 to 3,357)	7,698 (3,224 to 10,507)	3,080 (1,305 to 4,216)	4,618 (1,728 to 6,509)	185.8 (-40.3 to 535.9)	161.6 (-47.7 to 627.7)	204.6 (-34.9 to 566.8)
		Age-standardized (rate per 100,000)	5.2 (3.5 to 9.9)	4.6 (2.5 to 10.4)	5.9 (3.9 to 10.6)	9.6 (3.9 to 13.2)	7.9 (3.2 to 10.9)	11.3 (4.1 to 15.9)	82.6 (-51.1 to 255.5)	71.5 (-56.3 to 290.6)	91.7 (-48.1 to 277.9)
	YLDs	All ages (number)	4,864 (3,859 to 5,859)	2,645 (2,107 to 3,164)	2,219 (1,687 to 2,782)	15,101 (12,068 to 17,928)	7,742 (6,154 to 9,262)	7,359 (5,757 to 8,932)	210.5 (192 to 232.9)	192.7 (172.7 to 214.1)	231.6 (204.6 to 267.5)
		Age-standardized (rate per 100,000)	8.2 (6.6 to 9.8)	8.9 (7.2 to 10.6)	7.5 (5.8 to 9.2)	17.2 (13.7 to 20.5)	18 (14.4 to 21.4)	16.4 (12.8 to 19.9)	109.4 (101.3 to 119)	102.3 (92.8 to 112)	117.8 (104.9 to 133.9)

Data in parentheses are 95% Uncertainty Intervals (95% UIs)

DALYs= Disability-Adjusted Life Years; YLLs= Years of Life Lost; YLDs= Years Lived with Disability
