# Supplementary material for: National, subnational and risk attributed burden of chronic respiratory diseases in Iran from 1990 to 2019
Source: Respir Res. 2023 Mar 11;24:74. doi: 10.1186/s12931-023-02353-1 (PMC10006557; doi:10.1186/s12931-023-02353-1)
Supplement: Supplementary file 6 — Additional file 6: Table S2. Burden measures of CRDs for all ages number and ASR with percentage change by sex in all provinces, 1990 vs 2019. Data in parentheses are 95% Uncertainty Intervals (95% UIs); DALYs= Disability-Adjusted Life Years; YLLs= Years of Life Lost; YLDs= Years Lived with Disability [file 12931_2023_2353_MOESM6_ESM.pdf]

Province	Measure	Age-standardized rate (per 100,000)						% Change (1990 to 2019)		
		1990			2019					
		Both	Female	Male	Both	Female	Male	Both	Female	Male
Alborz	Incidence	924.3 (797.4 to 1074.2)	907.1 (783 to 1050.3)	941.9 (803 to 1102.9)	909.6 (773.9 to 1067)	895.3 (767.8 to 1048.4)	925.8 (776.2 to 1091.6)	-1.6 (-5.3 to 2.3)	-1.3 (-6.5 to 3.2)	-1.7 (-6.5 to 3.1)
	Prevalence	5633 (5008.5 to 6329.2)	5519.8 (4905 to 6220.6)	5746.7 (5076.3 to 6490)	4868.5 (4275.6 to 5582.2)	4634.5 (4072.7 to 5347.3)	5105.2 (4463.3 to 5846.7)	-13.6 (-17.9 to -9.5)	-16 (-21.1 to -10.8)	-11.2 (-16.5 to -5.7)
	Deaths	33.6 (25.5 to 44)	29 (17.8 to 41.1)	38.1 (28.2 to 52.7)	23.1 (19.5 to 27.4)	19.6 (15.5 to 25.2)	26.4 (21.1 to 32.7)	-31.4 (-49.1 to -7)	-32.2 (-56.1 to 19)	-30.8 (-53.7 to -1.9)
	DALYs	954 (779.1 to 1141.1)	857.1 (612.6 to 1075.4)	1043 (818.7 to 1312.5)	691.3 (600 to 793)	597.3 (506.2 to 713)	785.4 (660.1 to 918.3)	-27.5 (-40.9 to -11)	-30.3 (-45.1 to -0.8)	-24.7 (-43.4 to -3.9)
	YLLs	606.8 (449.3 to 788.5)	512 (295.6 to 699.4)	693.9 (487.6 to 960.7)	373 (315.7 to 442.7)	290 (230.9 to 384.7)	454.6 (362 to 559.6)	-38.5 (-54.8 to -13.1)	-43.4 (-62.8 to 8.7)	-34.5 (-57.3 to -2.5)
	YLDs	347.2 (268.1 to 430.7)	345.1 (267.5 to 431.4)	349.1 (264.4 to 439.2)	318.4 (247.2 to 397.2)	307.3 (238.3 to 381.5)	330.8 (255.3 to 418.1)	-8.3 (-13.3 to -2.8)	-11 (-17.4 to -3.6)	-5.3 (-11.4 to 1.2)
Ardebil	Incidence	955.1 (823.5 to 1123.5)	943.4 (817.8 to 1102.6)	963.9 (818.6 to 1144)	906.7 (775.8 to 1058.6)	893.5 (771.6 to 1036.8)	917.9 (775.9 to 1084.9)	-5.1 (-9.6 to -0.2)	-5.3 (-10.4 to 0.5)	-4.8 (-10.5 to 0.7)
	Prevalence	5630.1 (5010.4 to 6416.8)	5637.8 (4961 to 6389.4)	5605.6 (4932.9 to 6467.1)	4934.1 (4353.5 to 5618.2)	4752.8 (4171.7 to 5424.7)	5110.4 (4494.6 to 5852.2)	-12.4 (-16.8 to -7.8)	-15.7 (-21.1 to -10.2)	-8.8 (-14.2 to -3.1)
	Deaths	36.3 (28.9 to 48)	31.7 (22.1 to 45.9)	40.6 (30.8 to 59.3)	23.8 (20.6 to 29)	17.5 (14.4 to 22.3)	30.7 (25.5 to 40.6)	-34.3 (-51.2 to -17.1)	-44.8 (-62.7 to -19.2)	-24.2 (-50.1 to 2.6)
	DALYs	1017.2 (850.7 to 1239.2)	938.1 (718 to 1205)	1083.3 (861.1 to 1436.3)	720.1 (630.4 to 829.5)	597.8 (511 to 717.3)	850.8 (731.3 to 1016.9)	-29.2 (-42.2 to -17.1)	-36.3 (-49.3 to -17.8)	-21.5 (-41 to -2.9)
	YLLs	712.5 (570.6 to 920.8)	626.6 (438.6 to 876.5)	786.2 (590.4 to 1134.9)	413.5 (355.1 to 498.6)	294.2 (243.6 to 389.3)	541.2 (449.2 to 690.2)	-42 (-55.9 to -26.3)	-53 (-66.4 to -29.3)	-31.2 (-53.8 to -6.3)
	YLDs	304.7 (227.5 to 396.5)	311.5 (234.8 to 407.4)	297.1 (218.3 to 389.8)	306.6 (236.6 to 386.9)	303.5 (235.4 to 382.3)	309.6 (232.8 to 395.3)	0.6 (-6.1 to 7.5)	-2.5 (-10.2 to 5.8)	4.2 (-3.3 to 13)

Province	Measure	Age-standardized rate (per 100,000)						% Change (1990 to 2019)		
		1990			2019					
		Both	Female	Male	Both	Female	Male	Both	Female	Male
Bushehr	Incidence	898.6 (771.8 to 1056.6)	890.2 (774.1 to 1041.6)	907.1 (770 to 1067.8)	891.3 (757.3 to 1045.4)	887 (766.2 to 1034.2)	896.9 (753.2 to 1069.7)	-0.8 (-5.1 to 3.4)	-0.4 (-5.1 to 4.9)	-1.1 (-6.9 to 4.5)
	Prevalence	5269.8 (4641.4 to 5966.7)	5278.5 (4628.8 to 5985)	5266.6 (4628.7 to 6034.8)	4736.2 (4138.3 to 5447.7)	4647.5 (4082.1 to 5318.9)	4833.8 (4190.1 to 5582.6)	-10.1 (-14.7 to -5.5)	-12 (-17.5 to -6.2)	-8.2 (-14.2 to -2.1)
	Deaths	29.8 (22.9 to 41.1)	26.7 (17.9 to 41.3)	33.4 (25 to 50.3)	21.4 (18.6 to 25.9)	16.8 (13.7 to 20.1)	26.3 (22.1 to 35.9)	-28.2 (-45.9 to -6.2)	-37.1 (-61.3 to -2.1)	-21.2 (-41.4 to 6.9)
	DALYs	844.8 (690.9 to 1061.2)	797.1 (615.3 to 1057.6)	892.4 (718.4 to 1214.1)	655 (571 to 750.7)	580.2 (492.9 to 672.6)	733.4 (632.1 to 886.4)	-22.5 (-35.1 to -7.7)	-27.2 (-44.1 to -5.5)	-17.8 (-34.4 to 1.7)
	YLLs	547 (416.3 to 755.2)	494.4 (329.5 to 736)	599.6 (443.4 to 907.4)	355.2 (312.6 to 428.4)	280.4 (231.7 to 337.3)	432.3 (365.4 to 572.4)	-35.1 (-50.4 to -13.1)	-43.3 (-61.9 to -11.8)	-27.9 (-47.6 to 0.2)
	YLDs	297.8 (223.7 to 382.6)	302.8 (230.3 to 387.2)	292.9 (215.7 to 377.5)	299.9 (232.3 to 377.8)	299.8 (232.4 to 377.1)	301 (225.4 to 384)	0.7 (-5.4 to 7.6)	-1 (-8.1 to 6.7)	2.8 (-5.1 to 11.4)
Chahar Mahaal and Bakhtiari	Incidence	916.3 (790 to 1070.2)	885.9 (768.4 to 1035.3)	944.6 (801.2 to 1109.8)	898.2 (764.4 to 1052.7)	881.6 (761.9 to 1032.4)	913.3 (769.1 to 1083.5)	-2 (-5.8 to 2.2)	-0.5 (-5.1 to 4.3)	-3.3 (-8.7 to 2.1)
	Prevalence	5404.2 (4759.9 to 6127.6)	5200.2 (4606.1 to 5898)	5595.2 (4904.8 to 6364.4)	4876.1 (4307.5 to 5568.1)	4668.2 (4120.1 to 5324.3)	5081.7 (4467 to 5836.9)	-9.8 (-14.2 to -4.9)	-10.2 (-15.4 to -4.9)	-9.2 (-14.7 to -2.9)
	Deaths	32 (24.8 to 54.8)	26.1 (17.3 to 59.8)	38.3 (29 to 57)	16.6 (13.3 to 26.9)	11 (7.7 to 22.1)	22.7 (18.2 to 37.1)	-48.1 (-61.5 to -32.8)	-57.7 (-73.5 to -34.9)	-40.8 (-57.6 to -19.2)
	DALYs	888.1 (726.7 to 1266.8)	775.6 (593.4 to 1364.2)	993.6 (798.8 to 1331.5)	592.3 (496.6 to 757)	495.4 (398.8 to 703)	695.2 (579.8 to 900.2)	-33.3 (-44.5 to -21.8)	-36.1 (-50.7 to -19.1)	-30 (-44.7 to -14.3)
	YLLs	582.4 (451 to 984.3)	471.6 (317.8 to 1025.6)	686.9 (511.6 to 1046.3)	281 (225 to 444.2)	187.9 (133.1 to 380.9)	380.2 (303.1 to 604.7)	-51.8 (-63.3 to -37.5)	-60.2 (-73.5 to -37.8)	-44.7 (-60.7 to -23.6)
	YLDs	305.7 (229.6 to 392.1)	304 (231.4 to 386.5)	306.7 (228.6 to 397)	311.3 (241.2 to 393.7)	307.4 (239.3 to 384.6)	315 (238.5 to 406.7)	1.9 (-4 to 8.7)	1.1 (-5.9 to 9.1)	2.7 (-4.7 to 11.5)

Province	Measure	Age-standardized rate (per 100,000)						% Change (1990 to 2019)		
		1990			2019					
		Both	Female	Male	Both	Female	Male	Both	Female	Male
East Azarbayejan	Incidence	1057.4 (919.1 to 1224.8)	1048.5 (920 to 1211.9)	1063.5 (917.3 to 1241.9)	999 (869.3 to 1160.7)	987.7 (863.1 to 1147.8)	1009.2 (872.6 to 1184.9)	-5.5 (-9.9 to -0.8)	-5.8 (-10.9 to -0.9)	-5.1 (-10.5 to 0.1)
	Prevalence	6565.4 (5864.8 to 7416)	6591.7 (5862.3 to 7439.3)	6525.2 (5769.5 to 7410.1)	5740.8 (5102.6 to 6437.7)	5555.5 (4938.9 to 6250)	5922.8 (5237.7 to 6708.4)	-12.6 (-17 to -8)	-15.7 (-20.8 to -10.4)	-9.2 (-14.7 to -3)
	Deaths	72.2 (51.2 to 90.2)	66.5 (37.5 to 95.1)	77.5 (57.9 to 99.9)	49.3 (34.2 to 57.8)	43.2 (23.2 to 54.9)	55.6 (40.1 to 66.7)	-31.7 (-47.5 to -12.5)	-35 (-58.3 to -5.2)	-28.3 (-48.7 to -3.8)
	DALYs	1579.8 (1265.9 to 1884.3)	1477.6 (1031 to 1920.6)	1668 (1312.5 to 2070.9)	1114.5 (894.4 to 1264.6)	997.3 (744.2 to 1176.6)	1233.7 (994.7 to 1440.5)	-29.5 (-41 to -15.1)	-32.5 (-49 to -10.6)	-26 (-43.2 to -5)
	YLLs	1205.1 (890.7 to 1482.2)	1094.1 (676.6 to 1499.6)	1302.7 (966.4 to 1684.3)	736.4 (535 to 856)	622.9 (368.8 to 783.6)	851.9 (632.6 to 1026.7)	-38.9 (-52.6 to -21.3)	-43.1 (-60.9 to -15)	-34.6 (-54.2 to -7.2)
	YLDs	374.7 (282.7 to 475.7)	383.5 (292.8 to 483.5)	365.2 (271.6 to 472.9)	378.1 (294.3 to 468.2)	374.5 (293.6 to 462.8)	381.8 (295.3 to 475.8)	0.9 (-5.1 to 8)	-2.4 (-9.1 to 5.9)	4.5 (-3.2 to 14)
Fars	Incidence	900.8 (772.7 to 1049.3)	881.5 (762.2 to 1028.5)	920.8 (776.5 to 1075.8)	914.3 (780.6 to 1075.5)	902.3 (777.1 to 1053.2)	926.1 (789.3 to 1099.5)	1.5 (-2.5 to 5.4)	2.4 (-2.3 to 7.8)	0.6 (-4.7 to 5.4)
	Prevalence	5272.6 (4659.6 to 5984.5)	5189.6 (4579.8 to 5917.4)	5367.3 (4707.5 to 6116.6)	5039.8 (4454.9 to 5730.8)	4827.6 (4259.6 to 5502.1)	5250.7 (4612.9 to 5973.5)	-4.4 (-8.7 to 0.1)	-7 (-12.4 to -1.3)	-2.2 (-7.8 to 4.5)
	Deaths	30.7 (23.5 to 48.6)	25.3 (16.4 to 45.9)	37 (27.7 to 59.2)	24.5 (20.3 to 30.5)	19.9 (15.6 to 26)	28.9 (23.2 to 36.6)	-20.3 (-43.5 to 8.6)	-21.1 (-50.9 to 30.3)	-21.9 (-47.2 to 13.4)
	DALYs	860.4 (699.3 to 1177.9)	763 (569.1 to 1130.6)	961.1 (754.2 to 1373.7)	745.1 (642.7 to 868.5)	643.2 (539.9 to 780.6)	847.1 (707.9 to 1010.7)	-13.4 (-32 to 5.1)	-15.7 (-37.8 to 13.3)	-11.9 (-34.8 to 14)
	YLLs	560.8 (430 to 876.9)	463.1 (296.7 to 821.9)	661.4 (482.7 to 1116.2)	415.3 (343.7 to 516.5)	320.3 (251.9 to 445.4)	509.8 (409.1 to 649.6)	-25.9 (-47.4 to 1.3)	-30.8 (-55 to 17.2)	-22.9 (-49.4 to 16.2)
	YLDs	299.6 (227.8 to 386.3)	299.9 (227.3 to 387.1)	299.8 (224 to 389.3)	329.9 (255.9 to 411.9)	322.8 (251.9 to 402.2)	337.3 (259 to 428.3)	10.1 (2.6 to 18)	7.6 (-0.7 to 17)	12.5 (3.8 to 22.5)

Province	Measure	Age-standardized rate (per 100,000)						% Change (1990 to 2019)		
		1990			2019					
		Both	Female	Male	Both	Female	Male	Both	Female	Male
Gilan	Incidence	922 (791.2 to 1070.7)	888.4 (769.9 to 1033)	956.7 (811.2 to 1116)	928.1 (796.6 to 1089.6)	901.3 (777.2 to 1048.7)	954.6 (808.6 to 1138.7)	0.7 (-3.7 to 5)	1.4 (-3.6 to 6.2)	-0.2 (-5.8 to 5.7)
	Prevalence	5415.7 (4751.6 to 6209)	5197.1 (4536.8 to 5917.9)	5659.4 (4970.2 to 6449.1)	5144.7 (4534.1 to 5886.2)	4803.1 (4235.7 to 5443.9)	5489.3 (4824.4 to 6278.9)	-5 (-9.5 to 0.1)	-7.6 (-12.9 to -1.8)	-3 (-9.2 to 3.8)
	Deaths	34.1 (27.5 to 44.6)	27.2 (19.2 to 38.7)	43.3 (33.8 to 59.4)	23.4 (19.9 to 27.2)	17.7 (14.3 to 22.4)	29.1 (23.8 to 35.3)	-31.3 (-48.9 to -12.3)	-34.8 (-55.8 to -0.9)	-32.8 (-53.1 to -9.1)
	DALYs	925.6 (776.1 to 1120.1)	780.6 (607.3 to 997.4)	1092.2 (884.6 to 1384.4)	734.3 (640.9 to 839.6)	600.2 (508.1 to 705.6)	871.7 (739.8 to 1013.2)	-20.7 (-34.8 to -6.5)	-23.1 (-38.8 to -1.2)	-20.2 (-39.4 to 0.1)
	YLLs	628.3 (506.1 to 808.7)	489.1 (341.1 to 691.5)	788.3 (611.4 to 1078.8)	405.4 (344.6 to 471.9)	288.1 (233.4 to 368.5)	525.4 (424.9 to 637.3)	-35.5 (-51.8 to -16.5)	-41.1 (-59.3 to -9.3)	-33.4 (-55 to -6.3)
	YLDs	297.3 (222.7 to 382.9)	291.5 (219 to 375.3)	303.9 (223.6 to 393.6)	328.9 (254.1 to 415.4)	312.1 (240.4 to 394.7)	346.3 (263.5 to 441.8)	10.6 (3.1 to 18.9)	7.1 (-0.5 to 15.9)	14 (4.1 to 25.4)
Golestan	Incidence	979.6 (847.8 to 1140.8)	962.2 (838.7 to 1118.2)	995.6 (848.3 to 1170)	956.6 (836.4 to 1110.7)	943 (825.5 to 1089.5)	969.7 (841.9 to 1128.7)	-2.3 (-6.8 to 2)	-2 (-7.2 to 3.7)	-2.6 (-8.1 to 2.8)
	Prevalence	5880 (5184.2 to 6736.5)	5862.3 (5175.8 to 6688.4)	5898 (5180.8 to 6786.2)	5484.5 (4874.1 to 6184)	5295.9 (4715.5 to 6016.9)	5676.7 (5062.7 to 6407)	-6.7 (-11.1 to -2.2)	-9.7 (-14.5 to -3.8)	-3.8 (-9.2 to 2.1)
	Deaths	41.4 (33 to 51.4)	34 (23.7 to 47.7)	49.9 (37.5 to 65.4)	34.4 (27.7 to 39.2)	26.1 (16.2 to 32.5)	43.1 (35.3 to 51.2)	-16.9 (-36.7 to 8.1)	-23.4 (-53.6 to 14.4)	-13.5 (-37.8 to 18.6)
	DALYs	1132.2 (944 to 1338.1)	998.7 (754.2 to 1235.9)	1266.1 (1006.9 to 1584.7)	973.9 (827.4 to 1104.8)	805.9 (620.4 to 936.5)	1152.7 (961.6 to 1326.8)	-14 (-28.5 to 4.7)	-19.3 (-38.1 to 5.8)	-9 (-30.6 to 15.5)
	YLLs	816.7 (652.2 to 999.4)	679.4 (463.9 to 901.5)	954.7 (715.1 to 1261.5)	634.1 (506.6 to 722.9)	473.9 (295.1 to 587.9)	804.4 (647.4 to 955.7)	-22.4 (-40.6 to 1.6)	-30.3 (-54 to 6.1)	-15.7 (-40.4 to 16.5)
	YLDs	315.4 (233.8 to 411.7)	319.3 (239.6 to 414.2)	311.4 (228.2 to 409)	339.8 (261.3 to 424.9)	332 (258.3 to 416.8)	348.3 (266 to 437.8)	7.7 (0.7 to 16.2)	4 (-3.3 to 12.8)	11.9 (3.1 to 22.9)

Province	Measure	Age-standardized rate (per 100,000)						% Change (1990 to 2019)		
		1990			2019					
		Both	Female	Male	Both	Female	Male	Both	Female	Male
Hamadan	Incidence	950.8 (822.7 to 1111.9)	915.8 (792.8 to 1064.8)	982.5 (836.1 to 1164)	916.7 (787.3 to 1079)	891 (772.9 to 1050.4)	942.4 (799.3 to 1118.6)	-3.6 (-8 to 0.9)	-2.7 (-8 to 3.5)	-4.1 (-10.5 to 1.5)
	Prevalence	5698.5 (5025.9 to 6488.9)	5463.4 (4849.1 to 6188.5)	5906.9 (5212.6 to 6753.4)	5166 (4568.5 to 5888.9)	4800.1 (4244.7 to 5488.7)	5537.6 (4882.6 to 6308.4)	-9.3 (-13.5 to -5.2)	-12.1 (-17.4 to -6.9)	-6.3 (-11.6 to -0.5)
	Deaths	40.1 (32.3 to 54.7)	34.1 (23.2 to 55.1)	45.5 (35 to 61.9)	27 (22.5 to 32.7)	20.8 (16.7 to 30.3)	33.4 (26.8 to 40.3)	-32.7 (-47.3 to -14.9)	-38.9 (-57.7 to -1.1)	-26.6 (-47.7 to 2.3)
	DALYs	1102.8 (922.7 to 1359.3)	943.2 (731.1 to 1320.1)	1244.4 (1005 to 1568)	829.7 (718.9 to 951.6)	666 (565.9 to 839.9)	999 (841.8 to 1164.6)	-24.8 (-37.2 to -11.8)	-29.4 (-44.4 to -5.8)	-19.7 (-38.1 to 3.5)
	YLLs	784.5 (635.7 to 1045.2)	630.4 (431.4 to 1004.7)	921.7 (704.7 to 1247.8)	494.3 (415.1 to 601.3)	348.2 (280.6 to 517.8)	644.8 (518.8 to 783)	-37 (-51.1 to -20.3)	-44.8 (-61.1 to -10.6)	-30 (-51.2 to -1.7)
	YLDs	318.3 (240.4 to 410.4)	312.7 (238.4 to 402.3)	322.7 (238.2 to 418.7)	335.3 (260.5 to 416.8)	317.8 (249.3 to 398.4)	354.2 (271.6 to 446.9)	5.3 (-1.6 to 12.8)	1.6 (-6 to 10.2)	9.8 (0.9 to 20)
Hormozgan	Incidence	852.5 (731.4 to 1001.6)	852.5 (733.7 to 1003.4)	853.9 (720.8 to 1005.5)	858.7 (728.5 to 1019.2)	839.8 (715 to 988.1)	877.5 (738.1 to 1045.1)	0.7 (-3.7 to 5)	-1.5 (-6.8 to 4.2)	2.8 (-2.3 to 7.8)
	Prevalence	4921 (4349.8 to 5590.1)	4957.7 (4380 to 5636.2)	4893.2 (4300.8 to 5603.3)	4490.8 (3933.9 to 5198.6)	4257.2 (3720.9 to 4938.9)	4724.2 (4139.1 to 5475.6)	-8.7 (-13.3 to -4.3)	-14.1 (-19.7 to -8.4)	-3.5 (-8.9 to 2)
	Deaths	29.5 (22.2 to 43.9)	24.2 (16.3 to 38)	35.2 (25.6 to 57)	16.9 (14.2 to 21.6)	14.6 (11.8 to 20.2)	19.4 (15.6 to 26.4)	-42.9 (-56.6 to -23.4)	-39.6 (-58.1 to -6.3)	-45 (-61.6 to -21.1)
	DALYs	862.4 (680.3 to 1126.9)	762.8 (579 to 1002)	954.8 (728 to 1341.3)	585.9 (506.6 to 695)	512.1 (433.4 to 614.4)	663.1 (556.9 to 808.7)	-32.1 (-44.3 to -14.3)	-32.9 (-46.3 to -10.9)	-30.5 (-47.3 to -8.5)
	YLLs	585.1 (412.7 to 839)	481.6 (312.8 to 733.5)	681.2 (459.2 to 1102)	307.1 (261.3 to 387.9)	246 (199.9 to 343.3)	371.3 (298.8 to 493.7)	-47.5 (-60 to -24.1)	-48.9 (-63.7 to -14.9)	-45.5 (-62.4 to -16.6)
	YLDs	277.3 (208.9 to 358.6)	281.2 (214 to 362.6)	273.5 (203.1 to 353.6)	278.7 (215.7 to 350.8)	266.1 (203.9 to 336.6)	291.8 (221.7 to 372)	0.5 (-4.9 to 6.5)	-5.4 (-11.9 to 2)	6.7 (-0.6 to 14.5)

Province	Measure	Age-standardized rate (per 100,000)						% Change (1990 to 2019)		
		1990			2019					
		Both	Female	Male	Both	Female	Male	Both	Female	Male
Ilam	Incidence	849.3 (725.5 to 996)	838.5 (720.3 to 979.1)	858.5 (726.3 to 1013.6)	875.6 (747.4 to 1041.2)	870 (752.3 to 1023.5)	880.8 (740.9 to 1066.5)	3.1 (-0.9 to 7.5)	3.8 (-0.8 to 9.3)	2.6 (-3 to 8.1)
	Prevalence	4817.8 (4214.7 to 5511.6)	4774.1 (4175.3 to 5475.5)	4849.5 (4243 to 5565.7)	4552.6 (3971.7 to 5297.9)	4479.5 (3929.7 to 5156.7)	4622.7 (3987.4 to 5380.3)	-5.5 (-10.1 to -0.5)	-6.2 (-11.4 to -0.3)	-4.7 (-10.8 to 1.5)
	Deaths	24.9 (18.7 to 44)	21.3 (13.5 to 42.3)	27.9 (20.5 to 53.4)	18.7 (15.9 to 28.9)	14.9 (11.8 to 22.3)	21.8 (17.8 to 36.3)	-24.9 (-46.9 to -1.4)	-29.8 (-59.3 to 14.5)	-22 (-46.5 to 5.4)
	DALYs	742.1 (595.9 to 1096.2)	683.8 (512 to 1065.9)	785.1 (616.7 to 1258)	603.2 (516.8 to 760)	547 (456.8 to 699)	652.5 (550.7 to 877.2)	-18.7 (-34.3 to -3.6)	-20 (-40.7 to 4.8)	-16.9 (-35.6 to 1.8)
	YLLs	469.5 (352.2 to 818.2)	406.9 (258 to 811.7)	516.9 (377.1 to 977.9)	314.7 (268.1 to 475)	255.2 (203.4 to 394.4)	366.9 (301.5 to 593.1)	-33 (-49.9 to -10.5)	-37.3 (-61.5 to 4.1)	-29 (-49.7 to -2.3)
	YLDs	272.6 (203 to 351.1)	276.9 (207.3 to 355)	268.2 (198.1 to 346.1)	288.4 (223.2 to 365.6)	291.8 (226.6 to 370)	285.6 (216.7 to 365.1)	5.8 (-0.4 to 12.8)	5.4 (-1.8 to 13.4)	6.5 (-1.1 to 15.3)
Isfahan	Incidence	929.8 (802.5 to 1088.2)	895.4 (777.6 to 1040.9)	966.5 (821.3 to 1136.5)	931.9 (804.6 to 1093.7)	913.9 (794.5 to 1066.7)	949.4 (807.7 to 1126.6)	0.2 (-3.9 to 4.1)	2.1 (-2.7 to 6.9)	-1.8 (-7.3 to 3.5)
	Prevalence	5550.7 (4921 to 6277)	5328.2 (4696.8 to 6070)	5800 (5104.4 to 6575.6)	5155.2 (4545.7 to 5864.2)	4884.6 (4319.5 to 5590.7)	5419.3 (4754.2 to 6223.8)	-7.1 (-11.4 to -2.4)	-8.3 (-13.5 to -2.9)	-6.6 (-12.2 to -0.3)
	Deaths	37.1 (28.4 to 49.4)	31.6 (21.7 to 49)	43.4 (32.3 to 58.5)	27.1 (22.8 to 31.9)	22.4 (17.5 to 28.2)	31.5 (24.9 to 38.4)	-27 (-46.5 to 0.8)	-29.2 (-56.6 to 13.1)	-27.5 (-49.9 to 2.6)
	DALYs	964.7 (776.7 to 1182.3)	848.2 (639.4 to 1162.1)	1087.4 (865.8 to 1357.9)	783.1 (676.3 to 900.2)	673.2 (571.5 to 799.1)	890.5 (752.7 to 1040.4)	-18.8 (-33.8 to 0.6)	-20.6 (-39.9 to 5.6)	-18.1 (-37.3 to 4.5)
	YLLs	642.4 (493 to 846.8)	534.4 (352.4 to 822.1)	755 (557.6 to 1013.9)	442.4 (369.2 to 526.2)	344.4 (271.1 to 451.9)	537.6 (426.6 to 658)	-31.1 (-49.9 to -3.3)	-35.5 (-58.2 to 5.8)	-28.8 (-51.7 to 4.8)
	YLDs	322.3 (244.8 to 410.9)	313.8 (238.3 to 402.3)	332.4 (247.5 to 427.8)	340.8 (265.9 to 423.5)	328.7 (256.5 to 409.8)	352.9 (270.3 to 443)	5.7 (-0.8 to 13.2)	4.8 (-2.9 to 13.3)	6.2 (-1.9 to 15.1)

Province	Measure	Age-standardized rate (per 100,000)						% Change (1990 to 2019)		
		1990			2019					
		Both	Female	Male	Both	Female	Male	Both	Female	Male
Kerman	Incidence	1050 (914.3 to 1216.2)	1008.8 (878.8 to 1167.1)	1088.6 (936.4 to 1274.7)	1003 (871.3 to 1160.4)	977.3 (854.3 to 1119.8)	1027.9 (886.6 to 1204.9)	-4.5 (-9.1 to 0.1)	-3.1 (-8.6 to 2)	-5.6 (-11 to -0.1)
	Prevalence	6748.5 (6064 to 7536.9)	6399.6 (5727.9 to 7166.7)	7075.1 (6302.9 to 7905.2)	6042.7 (5426.9 to 6784.9)	5728.7 (5139 to 6444.7)	6354 (5714.8 to 7171.5)	-10.5 (-14.8 to -6)	-10.5 (-15.8 to -5.5)	-10.2 (-15.3 to -4.3)
	Deaths	63 (46.5 to 76.8)	49.5 (29.9 to 68.9)	78 (58.2 to 100.5)	58.5 (29.4 to 68.7)	47.6 (19.8 to 60.4)	69.4 (34.8 to 82.6)	-7.1 (-39.2 to 17.7)	-3.9 (-47.5 to 44.4)	-11 (-42.8 to 20.1)
	DALYs	1585.7 (1284.7 to 1867.2)	1299 (928.8 to 1630.3)	1865 (1479.8 to 2290.7)	1371 (885.8 to 1571.6)	1139.3 (721.3 to 1360.9)	1601.8 (998.8 to 1868.7)	-13.5 (-32.7 to 3.4)	-12.3 (-33.4 to 14)	-14.1 (-36.6 to 10.7)
	YLLs	1182.6 (910 to 1430.8)	911.4 (556.6 to 1225.9)	1448.1 (1070.9 to 1862.7)	948.2 (476.4 to 1112.9)	728.9 (325 to 930)	1166.4 (578 to 1412.1)	-19.8 (-48.6 to 3.1)	-20 (-52.1 to 19.3)	-19.5 (-50 to 11.9)
	YLDs	403.2 (309.1 to 507.2)	387.6 (298.4 to 486.5)	416.9 (315.3 to 528)	422.7 (333.4 to 510.8)	410.4 (322 to 498.7)	435.4 (340.3 to 538.4)	4.8 (-1.3 to 12.9)	5.9 (-1.8 to 15.5)	4.4 (-3.1 to 13.6)
Kermanshah	Incidence	956.8 (829 to 1111.4)	936.6 (815.7 to 1084.9)	973.7 (829.6 to 1141.5)	911.5 (782 to 1070.1)	901.1 (782.1 to 1043.7)	921.3 (781.4 to 1094.8)	-4.7 (-9.1 to -0.7)	-3.8 (-8.5 to 1.3)	-5.4 (-10.5 to -0.1)
	Prevalence	6023.8 (5377.2 to 6727.8)	5856 (5242.8 to 6573.6)	6154.1 (5429 to 6924.7)	5202 (4611.2 to 5872.6)	5003.7 (4452.3 to 5666.5)	5403.4 (4769.5 to 6131.7)	-13.6 (-17.9 to -9.5)	-14.6 (-19.5 to -9.1)	-12.2 (-17.5 to -6.5)
	Deaths	50.4 (41.4 to 61.8)	40.9 (29.4 to 56.9)	58.6 (46.1 to 75.9)	27 (23 to 31.4)	21.9 (17.3 to 27.1)	32 (26.2 to 38.5)	-46.3 (-57.8 to -31.8)	-46.3 (-63.1 to -21)	-45.5 (-60.4 to -25.3)
	DALYs	1338.1 (1144.2 to 1583.6)	1130.5 (879 to 1403.4)	1508.8 (1222.2 to 1867.1)	842.8 (729 to 957.8)	730.5 (618.8 to 852.5)	958.4 (801.9 to 1109.2)	-37 (-47.5 to -24.7)	-35.4 (-49 to -16.1)	-36.5 (-50.2 to -18.8)
	YLLs	974.3 (796.9 to 1202.8)	769.2 (539.4 to 1035.4)	1144.2 (885 to 1491.6)	491 (416.1 to 573.2)	382.9 (303.2 to 478.4)	601.3 (488.6 to 730.7)	-49.6 (-60.7 to -34.1)	-50.2 (-64.9 to -23.4)	-47.5 (-62.3 to -26.7)
	YLDs	363.8 (279.2 to 456.8)	361.3 (277.4 to 457.8)	364.5 (277.3 to 461.4)	351.8 (274.1 to 440.1)	347.6 (272.8 to 431.7)	357.1 (273.6 to 446.7)	-3.3 (-8.6 to 2.7)	-3.8 (-10.1 to 3.4)	-2 (-8.4 to 5.4)

Province	Measure	Age-standardized rate (per 100,000)						% Change (1990 to 2019)		
		1990			2019					
		Both	Female	Male	Both	Female	Male	Both	Female	Male
Khorasan-e-Razavi	Incidence	1008.1 (879 to 1171.3)	996.9 (866.5 to 1163.6)	1017 (869.6 to 1196.7)	987 (860.4 to 1148.3)	971 (847 to 1133.3)	1001.6 (865.7 to 1165.5)	-2.1 (-6 to 2.3)	-2.6 (-7.6 to 2.6)	-1.5 (-6.7 to 4.2)
	Prevalence	6307.5 (5668.3 to 7101.7)	6343.3 (5654.8 to 7145.2)	6266.2 (5578.7 to 7056)	5726.8 (5134.7 to 6410.2)	5531.8 (4949.5 to 6223)	5920.5 (5286.6 to 6706)	-9.2 (-13.6 to -4.9)	-12.8 (-17.9 to -7.5)	-5.5 (-10.8 to 0.2)
	Deaths	59.4 (47.9 to 71.2)	52.8 (33.9 to 69.8)	65.8 (51.5 to 84.2)	43.1 (29.3 to 50.3)	38.3 (19.6 to 48.3)	48 (36.1 to 57.5)	-27.5 (-46.9 to -8.9)	-27.6 (-52.4 to 14.3)	-27 (-48.5 to -2.2)
	DALYs	1476.1 (1234 to 1708.8)	1355.6 (1000.1 to 1658.6)	1584.5 (1279.4 to 1946.1)	1098.9 (860.2 to 1257.7)	985.8 (707.7 to 1160.9)	1215.5 (960.9 to 1421.5)	-25.6 (-39.5 to -10.8)	-27.3 (-43 to -1.8)	-23.3 (-42.1 to -3)
	YLLs	1108.7 (904.2 to 1318.9)	977.4 (626.1 to 1274.5)	1228.2 (948.2 to 1597.1)	715.6 (492.4 to 838.9)	605.1 (327.8 to 757.5)	829.5 (601.1 to 1001.4)	-35.5 (-54.1 to -17.1)	-38.1 (-58.5 to -3.5)	-32.5 (-54.1 to -7.4)
	YLDs	367.3 (280.4 to 462.1)	378.2 (290.6 to 474.5)	356.3 (268.4 to 455.4)	383.3 (300.4 to 469.4)	380.7 (301.3 to 466.4)	386 (296.8 to 480.4)	4.3 (-1.9 to 12)	0.7 (-6.2 to 9.1)	8.3 (0.8 to 18)
Khuzestan	Incidence	859.2 (736.4 to 1012.8)	828 (715.5 to 972.2)	889.9 (752.6 to 1049.2)	862.1 (736.3 to 1014.9)	833.4 (716 to 977.7)	889.9 (750.9 to 1063)	0.3 (-3.5 to 4.6)	0.6 (-3.6 to 5.5)	0 (-5.1 to 5.8)
	Prevalence	5056.9 (4455.1 to 5717.3)	4844.5 (4270.7 to 5539.5)	5267.7 (4619.3 to 6001)	4674.5 (4095.4 to 5357.4)	4390.5 (3830.6 to 5072.2)	4953.4 (4349.4 to 5687.5)	-7.6 (-11.5 to -3.2)	-9.4 (-14.5 to -3.7)	-6 (-11.1 to -0.5)
	Deaths	35.1 (28.2 to 44.6)	29.3 (20.8 to 42.5)	41.7 (31.9 to 56.6)	20.4 (17.6 to 24.9)	16.5 (13.2 to 21.6)	24.3 (20 to 31.2)	-41.9 (-56.9 to -24.5)	-43.7 (-63.7 to -14.1)	-41.5 (-59.7 to -18.2)
	DALYs	960.4 (804.5 to 1155.2)	837.7 (652.1 to 1056.3)	1082.5 (873.3 to 1388.6)	674 (589.6 to 772.6)	586 (497.7 to 695.4)	763 (653.7 to 897.3)	-29.8 (-42.2 to -17.2)	-30.1 (-46 to -10)	-29.5 (-45.3 to -11.9)
	YLLs	661 (528.1 to 832.7)	542.6 (382 to 759.5)	779 (584 to 1072.1)	364.7 (316.5 to 439)	286.6 (229.3 to 372.9)	443.9 (362.7 to 555.7)	-44.8 (-58.4 to -28.6)	-47.2 (-64.6 to -18.4)	-43 (-59.9 to -20.4)
	YLDs	299.4 (228.2 to 381.1)	295.1 (225.9 to 373.6)	303.5 (228.3 to 389.7)	309.2 (240.4 to 387.7)	299.4 (233 to 375.9)	319.1 (245.2 to 407.2)	3.3 (-2 to 9.6)	1.5 (-5.3 to 9.1)	5.2 (-1.6 to 12.9)

Province	Measure	Age-standardized rate (per 100,000)						% Change (1990 to 2019)		
		1990			2019					
		Both	Female	Male	Both	Female	Male	Both	Female	Male
Kohgiluyeh and Boyer-Ahmad	Incidence	868.1 (741.1 to 1019.2)	850.8 (731.6 to 1002.4)	887.2 (749.6 to 1035.7)	869.5 (741.2 to 1027.4)	856.4 (733.8 to 1011.3)	882 (740.4 to 1045.5)	0.2 (-3.6 to 4.5)	0.7 (-4.3 to 5.6)	-0.6 (-5.5 to 4.8)
	Prevalence	4951.5 (4359.7 to 5681.1)	4820.5 (4231.7 to 5557)	5092.5 (4455 to 5823.3)	4517.1 (3954.1 to 5201.6)	4336.3 (3794.8 to 4972)	4683.1 (4042.2 to 5415.1)	-8.8 (-13.3 to -4.1)	-10 (-15.4 to -4.2)	-8 (-13.6 to -1.6)
	Deaths	30.1 (22.2 to 47)	26 (16.3 to 47.2)	34.4 (24.9 to 52.6)	15.9 (12.4 to 25.7)	13.2 (9.2 to 25.3)	18.2 (13.7 to 27.9)	-47 (-63 to -29.4)	-49.2 (-69.6 to -16.7)	-47 (-65.3 to -23.4)
	DALYs	830 (671.4 to 1151.5)	733.2 (538.3 to 1130.5)	920.2 (724.4 to 1254)	558.5 (464.9 to 716.1)	497.7 (398.9 to 713.4)	613.3 (505.7 to 758.1)	-32.7 (-45.7 to -19.3)	-32.1 (-49 to -10)	-33.4 (-50.1 to -15.5)
	YLLs	559 (418 to 872.3)	467.8 (295.6 to 867.8)	643.4 (462.1 to 989.7)	277.3 (216.7 to 435.4)	223.8 (155 to 436)	325.5 (244.8 to 479.8)	-50.4 (-64 to -33.6)	-52.2 (-69.8 to -21.1)	-49.4 (-66.8 to -26.3)
	YLDs	271 (202.2 to 352.6)	265.4 (198.5 to 348.1)	276.8 (204.1 to 362.6)	281.2 (216.1 to 357.8)	273.9 (209.5 to 351.8)	287.8 (219 to 367.5)	3.8 (-3 to 10.9)	3.2 (-4.4 to 12.1)	4 (-3.6 to 12.8)
Kurdistan	Incidence	930.6 (802.5 to 1087.3)	920.1 (796.7 to 1076.1)	937.6 (799.4 to 1104.4)	896.9 (767.6 to 1053.3)	883.9 (766.4 to 1035.1)	909.8 (773.4 to 1071.9)	-3.6 (-8 to 0.5)	-3.9 (-8.6 to 0.7)	-3 (-8.1 to 2.6)
	Prevalence	5506.9 (4887.8 to 6221.6)	5443 (4831.7 to 6170.4)	5541 (4892.1 to 6294.1)	4869.2 (4284.6 to 5565)	4677 (4114.5 to 5342)	5062.3 (4421.1 to 5794.7)	-11.6 (-16.3 to -6.7)	-14.1 (-19.2 to -8.6)	-8.6 (-14.1 to -2.8)
	Deaths	44.5 (36.3 to 59.3)	34.1 (24.1 to 55.9)	53.7 (42 to 70.9)	24.5 (20.8 to 29.5)	20.4 (16.3 to 28.3)	28.6 (23 to 34.4)	-44.9 (-56.4 to -31.6)	-40.1 (-59.6 to -13)	-46.8 (-61.1 to -28.3)
	DALYs	1168.5 (986.6 to 1473.1)	991.9 (779.5 to 1399.2)	1312.5 (1068.1 to 1660.1)	743.8 (649.7 to 856.5)	651 (550.5 to 812.9)	837.5 (709.6 to 979.8)	-36.3 (-46.6 to -25.4)	-34.4 (-48.1 to -16.4)	-36.2 (-50.8 to -19.7)
	YLLs	855.9 (692.9 to 1150.1)	678.4 (486.3 to 1071.6)	1002.4 (771.8 to 1357.2)	429.2 (368.8 to 517)	343.1 (277 to 485)	515.6 (413.2 to 625.1)	-49.9 (-60.1 to -37)	-49.4 (-63 to -26.4)	-48.6 (-63.3 to -28.7)
	YLDs	312.6 (238.4 to 405.6)	313.4 (239.5 to 404.2)	310.1 (232.3 to 402.8)	314.6 (243.9 to 395.7)	307.9 (239.4 to 382.4)	321.9 (246.6 to 407)	0.7 (-5.1 to 7.2)	-1.8 (-8.8 to 6.5)	3.8 (-3.5 to 12.2)

Province	Measure	Age-standardized rate (per 100,000)						% Change (1990 to 2019)		
		1990			2019					
		Both	Female	Male	Both	Female	Male	Both	Female	Male
Lorestan	Incidence	853.5 (731.5 to 1006.8)	843.1 (728.7 to 988)	862.7 (725.7 to 1024.3)	877.4 (748.4 to 1039.4)	871.3 (751 to 1015.4)	881.7 (744 to 1050.6)	2.8 (-1 to 6.7)	3.4 (-1.2 to 8.1)	2.2 (-3 to 7.2)
	Prevalence	4936.6 (4348.9 to 5584)	4865.6 (4297.4 to 5551.8)	4994.9 (4398.4 to 5710.9)	4739.8 (4144.5 to 5442.5)	4597.1 (4039.7 to 5289.6)	4880.2 (4212 to 5651.2)	-4 (-8.4 to 0.8)	-5.5 (-10.9 to 0.3)	-2.3 (-7.7 to 3.9)
	Deaths	31.3 (24.5 to 51.2)	25.8 (17.4 to 49.2)	36.5 (27.3 to 64.7)	19.5 (15.8 to 30.7)	13.2 (9.6 to 22.6)	26.1 (21 to 41.5)	-37.7 (-52.9 to -19.9)	-49 (-67.1 to -18.8)	-28.6 (-50.6 to -0.6)
	DALYs	880.6 (718 to 1255.2)	774.1 (584.4 to 1215.7)	972.9 (775.4 to 1468.6)	639.6 (538.4 to 808.5)	526.7 (427.9 to 708.5)	758.2 (633.6 to 993.3)	-27.4 (-40.8 to -14.6)	-32 (-46.6 to -11.1)	-22.1 (-40.5 to -1)
	YLLs	598.2 (468.6 to 985)	491.2 (327.6 to 901.6)	691.7 (514 to 1205)	338.1 (273.5 to 519.8)	228.9 (166.1 to 405.5)	452.8 (361 to 700.1)	-43.5 (-57.3 to -26.5)	-53.4 (-68.7 to -24.6)	-34.5 (-54.9 to -6.5)
	YLDs	282.4 (213.5 to 363.4)	282.9 (214.4 to 362.2)	281.2 (209.6 to 365.5)	301.5 (233.7 to 379.3)	297.8 (230.3 to 374.7)	305.4 (230.8 to 388.8)	6.8 (1 to 13.4)	5.3 (-1.9 to 13)	8.6 (1.1 to 16.9)
Markazi	Incidence	977.6 (844.8 to 1138.2)	939.6 (818.5 to 1083.6)	1012.4 (861.2 to 1177.5)	918.1 (790.5 to 1078.4)	896.5 (779.1 to 1043.9)	938.9 (799.8 to 1111.7)	-6.1 (-10.5 to -1.1)	-4.6 (-9.5 to 0.6)	-7.3 (-12.4 to -1.7)
	Prevalence	6050.3 (5365.4 to 6850.1)	5742.2 (5068 to 6485.8)	6338.3 (5587.5 to 7202)	5158 (4557.6 to 5842.7)	4820.2 (4245.2 to 5510.8)	5498.2 (4830.4 to 6271.8)	-14.7 (-19.1 to -10.2)	-16.1 (-21.4 to -9.9)	-13.3 (-18.6 to -7.7)
	Deaths	48.5 (39.3 to 60)	39.6 (27.9 to 54.3)	57.4 (44.3 to 74.1)	25.7 (21.5 to 30.4)	20.7 (16.3 to 26.8)	30.8 (24.5 to 37.5)	-46.9 (-57.6 to -33.6)	-47.5 (-62.3 to -21.9)	-46.4 (-60.1 to -27.3)
	DALYs	1244 (1058.8 to 1466.1)	1044.8 (818.2 to 1299.6)	1434.6 (1157.4 to 1771.5)	783.5 (683.6 to 898.9)	656.9 (558.3 to 784.7)	913.3 (770.3 to 1063.4)	-37 (-46.6 to -25.9)	-37.1 (-49.5 to -18.3)	-36.3 (-49.3 to -19.5)
	YLLs	900.5 (731.6 to 1108.9)	711.6 (502.3 to 954.4)	1082 (822.3 to 1398.1)	447.7 (374.9 to 533.2)	337.7 (267.5 to 447.1)	560.2 (443.6 to 680.8)	-50.3 (-60.6 to -37.1)	-52.5 (-65.4 to -27)	-48.2 (-62.2 to -28.2)
	YLDs	343.6 (260.8 to 442.1)	333.2 (255.3 to 429.1)	352.6 (261.9 to 455.4)	335.8 (262.4 to 415.8)	319.3 (250.7 to 396.7)	353.1 (271.4 to 445.3)	-2.3 (-8.4 to 4.8)	-4.2 (-10.9 to 4)	0.1 (-7.2 to 8.9)

Province	Measure	Age-standardized rate (per 100,000)						% Change (1990 to 2019)		
		1990			2019					
		Both	Female	Male	Both	Female	Male	Both	Female	Male
Mazandaran	Incidence	915.7 (784.2 to 1072.1)	891.4 (772.6 to 1046.3)	939.3 (791.5 to 1099.9)	911.8 (775.8 to 1073.4)	897.3 (773.6 to 1056.9)	925.6 (780.6 to 1098.5)	-0.4 (-4.4 to 3.9)	0.7 (-4.1 to 5.5)	-1.5 (-6.5 to 4)
	Prevalence	5379.9 (4731.1 to 6140.6)	5252.1 (4607.6 to 6002.9)	5515.1 (4844.2 to 6286.1)	4904.5 (4294.2 to 5601)	4741.9 (4156 to 5467.3)	5067 (4413.5 to 5768.9)	-8.8 (-13.2 to -4.3)	-9.7 (-15.1 to -4)	-8.1 (-13 to -2)
	Deaths	28.1 (21.9 to 38.3)	23.5 (15.8 to 36.3)	33.8 (25.5 to 49.6)	19 (16.1 to 23.5)	14.8 (11.6 to 20)	23.4 (19.3 to 29.3)	-32.2 (-50.5 to -10.2)	-37.2 (-59.3 to 1.1)	-30.6 (-52.3 to -5.1)
	DALYs	806.2 (671 to 1003.5)	723.4 (556.4 to 970.3)	896.7 (728.3 to 1186)	631.4 (548.1 to 737.6)	550.9 (463.5 to 671.5)	713.7 (605.6 to 843.4)	-21.7 (-36.5 to -7.4)	-23.8 (-39.8 to -1.1)	-20.4 (-38.2 to -2.3)
	YLLs	503.8 (396.6 to 686.5)	421.4 (277 to 646)	594.1 (447.3 to 871.8)	323.6 (275.3 to 397.2)	247.1 (193.7 to 340.7)	401.8 (324.7 to 498.9)	-35.8 (-52.6 to -14.2)	-41.4 (-60.8 to -2.5)	-32.4 (-54 to -5.2)
	YLDs	302.4 (229.2 to 389.2)	302 (231.8 to 384.3)	302.7 (225.7 to 390.8)	307.8 (236.8 to 384.7)	303.8 (235.8 to 380.6)	311.9 (236.1 to 397.1)	1.8 (-4.2 to 8.5)	0.6 (-6.3 to 8.7)	3.1 (-4.3 to 11)
North Khorasan	Incidence	978.7 (843.6 to 1133.4)	973.2 (845.1 to 1127.3)	982.7 (839.6 to 1139.3)	943.8 (812.5 to 1099.4)	944.8 (819.8 to 1095.2)	943.7 (806 to 1100.7)	-3.6 (-7.9 to 1.7)	-2.9 (-8 to 2.8)	-4 (-9.1 to 1.8)
	Prevalence	6011.3 (5346.5 to 6793.6)	6022.1 (5344.7 to 6839.1)	5994.1 (5327.9 to 6782.9)	5392.7 (4802 to 6069.4)	5299 (4735.9 to 5956)	5494.2 (4857.6 to 6214.7)	-10.3 (-14.6 to -5.9)	-12 (-17.2 to -6.7)	-8.3 (-14 to -2.3)
	Deaths	52.1 (42.6 to 62.3)	46.7 (31.1 to 62.8)	57.2 (44 to 75)	32.5 (27 to 37.1)	30.7 (20.7 to 38.3)	34.6 (28.6 to 40.7)	-37.6 (-50.8 to -21.3)	-34.3 (-54.4 to 7.3)	-39.4 (-56.4 to -18.5)
	DALYs	1373.4 (1168.3 to 1590.2)	1270.7 (921.4 to 1564.2)	1464.1 (1190 to 1815)	931.8 (809 to 1047.8)	876.9 (706.9 to 1017.1)	994 (847.6 to 1150.8)	-32.2 (-42.7 to -19.5)	-31 (-44.7 to -3.4)	-32.1 (-47.6 to -15.1)
	YLLs	1037.1 (849.6 to 1228.4)	927.6 (588.2 to 1194.7)	1135.1 (874.2 to 1484.9)	581.7 (485.7 to 664.5)	523.7 (366.5 to 648.5)	645.3 (534.1 to 759.7)	-43.9 (-55.8 to -29.5)	-43.5 (-59.3 to -7.2)	-43.1 (-59.3 to -22.2)
	YLDs	336.3 (253.5 to 430.6)	343.2 (261.2 to 440.6)	329 (245.4 to 429.4)	350.2 (271.5 to 434.8)	353.2 (275.4 to 433.9)	348.7 (265.6 to 441.7)	4.1 (-2.5 to 11.8)	2.9 (-4.3 to 11.8)	6 (-2.4 to 15.5)

Province	Measure	Age-standardized rate (per 100,000)						% Change (1990 to 2019)		
		1990			2019					
		Both	Female	Male	Both	Female	Male	Both	Female	Male
Qazvin	Incidence	942.2 (812.3 to 1105.7)	934.6 (815.2 to 1084.9)	950.3 (810.7 to 1117.3)	919 (789.1 to 1078.7)	903.7 (780.4 to 1060.3)	931.7 (792 to 1108.5)	-2.5 (-6.7 to 1.5)	-3.3 (-9.1 to 2.1)	-2 (-6.8 to 2.6)
	Prevalence	5773 (5140.9 to 6515.4)	5788 (5156.9 to 6540.4)	5761.9 (5088.3 to 6523.3)	5070 (4489.2 to 5752.3)	4837.1 (4259.2 to 5527.9)	5298.5 (4662.5 to 6045.4)	-12.2 (-16.4 to -7.6)	-16.4 (-21.6 to -10.6)	-8 (-13.7 to -2.6)
	Deaths	36.4 (28.7 to 49.6)	31.9 (21.7 to 47.9)	41.7 (31.4 to 59.1)	26.3 (22.3 to 30.8)	17.3 (13.3 to 22)	36.5 (30.5 to 42.9)	-27.7 (-47.2 to -4.2)	-45.7 (-65.2 to -14.5)	-12.5 (-40.7 to 19.3)
	DALYs	1019.2 (847.4 to 1242.8)	938.7 (726.7 to 1206)	1101.2 (866 to 1408.7)	774 (674.6 to 888.7)	605.2 (511.5 to 718)	954.9 (827.9 to 1100.3)	-24.1 (-37.9 to -8.7)	-35.5 (-49.1 to -15.1)	-13.3 (-34 to 10)
	YLLs	684.2 (544.7 to 898.9)	597.4 (399.2 to 846.6)	772.5 (575.4 to 1065.7)	444.2 (378.7 to 522.2)	285.3 (222.6 to 374.3)	615.6 (509.9 to 728.4)	-35.1 (-51.5 to -14.2)	-52.2 (-68.2 to -23.6)	-20.3 (-46 to 13.3)
	YLDs	335 (254 to 425.7)	341.3 (260.7 to 433.4)	328.8 (246.2 to 426.6)	329.8 (255.9 to 410.3)	319.9 (247.2 to 398.6)	339.3 (259.6 to 428.1)	-1.5 (-7.3 to 4.6)	-6.3 (-13.3 to 1)	3.2 (-3.7 to 11.1)
Qom	Incidence	959.8 (830.7 to 1110.7)	951.7 (825.3 to 1102.6)	968.1 (830.2 to 1126.8)	939.4 (808.5 to 1098.4)	939.3 (809.4 to 1087.9)	942.7 (799.8 to 1113.5)	-2.1 (-6.7 to 2.6)	-1.3 (-6.5 to 4)	-2.6 (-8.1 to 3.2)
	Prevalence	5964.5 (5329.6 to 6680.9)	5879.5 (5231.4 to 6643)	6051.1 (5412.5 to 6793.1)	5131.7 (4549.5 to 5816.7)	4969.1 (4390.7 to 5681.8)	5302.8 (4682.1 to 6042.7)	-14 (-18.1 to -9.6)	-15.5 (-20.9 to -10.2)	-12.4 (-17.1 to -6.8)
	Deaths	54.5 (36.9 to 68.7)	47.5 (27.8 to 67.5)	61.3 (43.2 to 82.1)	28.5 (22.1 to 32.8)	26.1 (17.3 to 32.3)	31 (24 to 37.1)	-47.7 (-59 to -32.5)	-45 (-62.7 to -13.6)	-49.5 (-63.6 to -29.6)
	DALYs	1347.9 (990 to 1614.2)	1180.4 (819.7 to 1500.8)	1505.2 (1089.8 to 1925.1)	808.3 (690.2 to 921.5)	731.3 (596 to 858.2)	886.3 (738.7 to 1027.1)	-40 (-50 to -26.4)	-38 (-51.1 to -12.2)	-41.1 (-55.1 to -23.3)
	YLLs	987.4 (654 to 1235.6)	821.9 (475.9 to 1131.8)	1142.9 (753 to 1543.5)	466.5 (365.6 to 537.8)	395.9 (269.8 to 490.7)	536.5 (406.5 to 646.9)	-52.8 (-62.9 to -37.1)	-51.8 (-66.1 to -16)	-53.1 (-66.8 to -32.3)
	YLDs	360.5 (276.1 to 451.7)	358.4 (276.7 to 452.4)	362.3 (273.1 to 459.6)	341.8 (266.2 to 425.8)	335.4 (262.7 to 416.3)	349.8 (269.1 to 437.1)	-5.2 (-10.5 to 0.4)	-6.4 (-12.6 to 0.4)	-3.4 (-9.3 to 2.8)

Province	Measure	Age-standardized rate (per 100,000)						% Change (1990 to 2019)		
		1990			2019					
		Both	Female	Male	Both	Female	Male	Both	Female	Male
Semnan	Incidence	984 (848.9 to 1133.9)	953.7 (823.6 to 1098.5)	1013.5 (864.6 to 1179.3)	938.1 (807 to 1098.4)	926.2 (804 to 1073.8)	947 (807.3 to 1122.5)	-4.7 (-8.8 to 0)	-2.9 (-7.9 to 2.4)	-6.6 (-11.7 to -0.3)
	Prevalence	6123.1 (5441.5 to 6930.2)	5876.3 (5211.3 to 6622.9)	6378.1 (5653.9 to 7229.5)	5225.1 (4622.3 to 5917.2)	5026.3 (4434.6 to 5701.6)	5415 (4767.3 to 6173.2)	-14.7 (-19 to -10.3)	-14.5 (-19.8 to -9)	-15.1 (-20 to -9.7)
	Deaths	45.4 (34.8 to 59.6)	35.9 (23.2 to 54.5)	57.7 (44.5 to 76.4)	29.5 (24.2 to 34.2)	20.3 (14.6 to 25.2)	40.1 (32.3 to 46.7)	-35 (-52.1 to -14.2)	-43.4 (-65.7 to -10.6)	-30.4 (-49.3 to -6.9)
	DALYs	1183.1 (956.8 to 1419.4)	983.9 (738.5 to 1283.9)	1401.4 (1108.7 to 1770.9)	829.6 (702.8 to 951.9)	659.9 (548.9 to 773.6)	1011.3 (839.6 to 1161.7)	-29.9 (-42.7 to -14.9)	-32.9 (-50.8 to -10.5)	-27.8 (-43.6 to -9.4)
	YLLs	833.5 (637.8 to 1064.9)	646 (425.5 to 945)	1040.5 (774.1 to 1392)	489.9 (405.3 to 564.4)	329.1 (245.1 to 412.8)	663.7 (536.8 to 774.1)	-41.2 (-55.9 to -22)	-49.1 (-67.4 to -17.3)	-36.2 (-54.1 to -11.4)
	YLDs	349.6 (264.6 to 444)	337.9 (255.5 to 431.9)	360.9 (269.9 to 462.5)	339.7 (263.6 to 425.5)	330.9 (256.5 to 414.8)	347.5 (265.7 to 438.3)	-2.8 (-8.7 to 3.6)	-2.1 (-9.2 to 5.7)	-3.7 (-10.5 to 4.1)
Sistan and Baluchistan	Incidence	1111.5 (966.6 to 1296.4)	1081.8 (936.3 to 1257.9)	1139.3 (980.8 to 1345.3)	1015.1 (883.1 to 1176.3)	997.7 (868.4 to 1154)	1030.5 (885.6 to 1200.8)	-8.7 (-13.6 to -4)	-7.8 (-12.6 to -2.1)	-9.6 (-15.6 to -3.9)
	Prevalence	7107.7 (6351.1 to 8009.7)	7037.9 (6274.1 to 7922.5)	7167.2 (6358.8 to 8145.6)	6040.5 (5426.6 to 6782.8)	5826.1 (5225.9 to 6578)	6247.8 (5593.7 to 7063.3)	-15 (-18.8 to -10.7)	-17.2 (-21.9 to -12.1)	-12.8 (-17.9 to -7)
	Deaths	95.9 (46.2 to 122.8)	87.5 (28.4 to 128.7)	103.5 (57.1 to 136.1)	47.1 (27.9 to 56.6)	42.1 (18.1 to 55.3)	52 (34.6 to 65.8)	-50.9 (-62.7 to -33.8)	-51.9 (-68.7 to -26.1)	-49.7 (-64.6 to -26.6)
	DALYs	2264.1 (1336.5 to 2765.2)	2075.1 (974.9 to 2787.8)	2414.8 (1411.4 to 3084.4)	1300.1 (923.2 to 1513.1)	1200.2 (750.1 to 1471.9)	1400.1 (1024.2 to 1686)	-42.6 (-53.9 to -21.6)	-42.2 (-57.8 to -17.4)	-42 (-57.3 to -14.4)
	YLLs	1858.2 (938.4 to 2346.2)	1668.1 (582.2 to 2389.5)	2010.6 (1041.4 to 2667.5)	907.1 (546.4 to 1086.3)	811.2 (373.5 to 1073.5)	1003.2 (642.3 to 1260.7)	-51.2 (-62.8 to -28)	-51.4 (-67.6 to -21.9)	-50.1 (-65.3 to -18.9)
	YLDs	405.9 (305.1 to 520.8)	406.9 (310 to 523.8)	404.2 (299.9 to 520.1)	393 (305.5 to 489.5)	388.9 (304.3 to 482.9)	396.9 (304 to 501.3)	-3.2 (-8.7 to 3.4)	-4.4 (-10.5 to 2.8)	-1.8 (-8.1 to 5.9)

Province	Measure	Age-standardized rate (per 100,000)						% Change (1990 to 2019)		
		1990			2019					
		Both	Female	Male	Both	Female	Male	Both	Female	Male
South Khorasan	Incidence	1089.2 (939 to 1281.4)	1029.1 (894.5 to 1193)	1143.1 (975.2 to 1355.7)	1017.8 (884.3 to 1185.5)	981.7 (853.7 to 1134.1)	1051.6 (903.1 to 1236.4)	-6.6 (-11.4 to -1.6)	-4.6 (-9.9 to 1.2)	-8 (-13.9 to -2.3)
	Prevalence	6899.5 (6144.6 to 7837.5)	6532.8 (5804 to 7421.3)	7239.2 (6386.6 to 8232.3)	5932.4 (5278.1 to 6705.9)	5613.2 (4961.6 to 6320.7)	6243 (5540.2 to 7086.3)	-14 (-18 to -9.6)	-14.1 (-18.9 to -8.5)	-13.8 (-18.7 to -8.2)
	Deaths	70.2 (43.6 to 87.6)	56.6 (27.6 to 77.8)	83.8 (52.5 to 110.9)	42.9 (25.2 to 50.4)	34.2 (16.8 to 43)	53.5 (32.8 to 63.8)	-38.8 (-52.5 to -24.1)	-39.6 (-58.3 to -7)	-36.1 (-53.3 to -15.7)
	DALYs	1737.3 (1233.7 to 2067.1)	1463.1 (893.7 to 1862.6)	1992.5 (1386.6 to 2485.8)	1102.4 (796 to 1256)	946.4 (644 to 1114)	1274.8 (917.9 to 1487)	-36.5 (-47 to -25.6)	-35.3 (-49.5 to -11.1)	-36 (-49.5 to -20.3)
	YLLs	1358.6 (854.1 to 1683.2)	1095.6 (536.2 to 1486.9)	1603.8 (1002.1 to 2107.1)	723.8 (412.5 to 844.3)	577.1 (290.1 to 719.8)	887.3 (522.3 to 1071.8)	-46.7 (-57.9 to -33.6)	-47.3 (-62.5 to -16.5)	-44.7 (-59.2 to -26.2)
	YLDs	378.7 (283 to 488.9)	367.5 (278.1 to 475.3)	388.7 (285.9 to 504.5)	378.6 (293.1 to 471.7)	369.3 (288.9 to 460.3)	387.5 (295.1 to 491.4)	0 (-6.6 to 7.8)	0.5 (-6.6 to 9)	-0.3 (-8.2 to 9)
Tehran	Incidence	905.2 (777.3 to 1059.1)	871.4 (754.1 to 1027)	939.6 (800.1 to 1101)	925.4 (788.6 to 1085.8)	909.7 (783.1 to 1066.5)	940 (795.4 to 1107.2)	2.2 (-1.7 to 5.8)	4.4 (-0.5 to 8.9)	0 (-4.8 to 4.5)
	Prevalence	4976.6 (4351.8 to 5658.2)	4696.9 (4109.3 to 5420.1)	5264.5 (4577.8 to 6000.2)	4899.7 (4293.2 to 5649.5)	4578.6 (3989.1 to 5303.8)	5213.7 (4557 to 5964.8)	-1.5 (-5.6 to 2.8)	-2.5 (-8.3 to 3.3)	-1 (-6.1 to 4.6)
	Deaths	27.4 (19.8 to 37.9)	25.4 (14.9 to 40.9)	29.4 (21.3 to 43.6)	14.5 (11.9 to 17.6)	13.3 (10.2 to 18.2)	15.7 (12.1 to 19.3)	-47.1 (-62.2 to -26.4)	-47.5 (-67.8 to -9.9)	-46.5 (-65.8 to -24.1)
	DALYs	761.3 (619.2 to 931.2)	690.5 (508.9 to 938.3)	828.3 (659.8 to 1083.4)	553.2 (470.1 to 644.2)	500.8 (413.6 to 616.6)	605.7 (504.2 to 709.9)	-27.3 (-40.3 to -12.7)	-27.5 (-45 to -4.7)	-26.9 (-43.6 to -10.5)
	YLLs	465.6 (344.8 to 634.1)	411.3 (241.8 to 653.4)	515.8 (373.1 to 764.5)	227 (188.9 to 287.4)	194 (150.6 to 289.6)	260.1 (202.4 to 320.3)	-51.3 (-65.1 to -32.3)	-52.8 (-70.5 to -19.5)	-49.6 (-66.8 to -27.3)
	YLDs	295.7 (227 to 375.5)	279.3 (213.8 to 354.3)	312.5 (235.9 to 401.5)	326.2 (254.6 to 404.8)	306.8 (238.8 to 379.4)	345.6 (264.4 to 436.2)	10.3 (3.9 to 17.5)	9.9 (1.8 to 18.9)	10.6 (3.3 to 19.2)

Province	Measure	Age-standardized rate (per 100,000)						% Change (1990 to 2019)		
		1990			2019					
		Both	Female	Male	Both	Female	Male	Both	Female	Male
West Azarbayejan	Incidence	1020.7 (890.1 to 1182.6)	995.7 (868.3 to 1158.2)	1043.4 (898.6 to 1209.8)	951.1 (822.4 to 1107.1)	931.2 (813.5 to 1084.3)	970.3 (828.5 to 1136.3)	-6.8 (-11.4 to -2.5)	-6.5 (-11.5 to -1.4)	-7 (-12.4 to -1.6)
	Prevalence	6300.1 (5591.2 to 7071.7)	6167.8 (5493.7 to 6919.7)	6415.5 (5664.1 to 7262.3)	5351.7 (4757.2 to 6084)	5124.8 (4548.9 to 5826.7)	5583 (4942.4 to 6394.3)	-15.1 (-19.3 to -10.3)	-16.9 (-21.9 to -11.4)	-13 (-18.2 to -7)
	Deaths	67.5 (52.8 to 81.1)	56.6 (36.1 to 80.2)	78.6 (60.7 to 99.2)	38.5 (33 to 43.7)	31.6 (24.5 to 38.3)	46.1 (38.5 to 53.6)	-43 (-54.8 to -29)	-44.2 (-64.2 to -18.4)	-41.4 (-56.1 to -23.4)
	DALYs	1542.3 (1297.2 to 1791.7)	1335.2 (1011.3 to 1710.1)	1733.5 (1393.4 to 2100.5)	952 (838.4 to 1066.1)	819.4 (695.5 to 951.8)	1095.1 (943.5 to 1255.1)	-38.3 (-48 to -27.7)	-38.6 (-53.7 to -20.1)	-36.8 (-49.5 to -21.7)
	YLLs	1183.5 (955.5 to 1400.8)	978.9 (666.6 to 1338.9)	1373.2 (1068.3 to 1719.4)	606.7 (523.8 to 684.1)	483 (391.4 to 582)	740.1 (618.2 to 875.5)	-48.7 (-59.1 to -36.9)	-50.7 (-66.2 to -27.7)	-46.1 (-59.9 to -28.2)
	YLDs	358.8 (270.5 to 459.5)	356.4 (270.4 to 456.9)	360.2 (266.3 to 465.5)	345.4 (266.7 to 430)	336.4 (260.1 to 420.9)	355 (270.8 to 445.2)	-3.8 (-9.7 to 2.6)	-5.6 (-12.4 to 1)	-1.5 (-8.3 to 7.4)
Yazd	Incidence	961.4 (832.8 to 1122.1)	915.6 (793.7 to 1065.1)	1014.7 (873.6 to 1185.7)	931 (801.4 to 1090.8)	904.3 (779.5 to 1056.6)	955.8 (817 to 1125.1)	-3.2 (-7.8 to 0.8)	-1.2 (-6.6 to 3.8)	-5.8 (-11.3 to -0.8)
	Prevalence	5853 (5190 to 6638.3)	5536 (4898.3 to 6282.1)	6244 (5549.4 to 7042.2)	5173.8 (4592.5 to 5892.8)	4839.1 (4275.5 to 5500.8)	5494.1 (4833.4 to 6232.7)	-11.6 (-15.9 to -7.3)	-12.6 (-18 to -7.2)	-12 (-17.2 to -6.1)
	Deaths	49.6 (38.3 to 63.2)	37.1 (25.5 to 56.3)	67.4 (50.4 to 89.2)	34.6 (26.7 to 40.7)	24.4 (17.5 to 30.4)	46.9 (33.8 to 57)	-30.1 (-46.6 to -8.1)	-34.2 (-57.8 to 1)	-30.5 (-50 to -5.7)
	DALYs	1235.5 (1015.7 to 1481.8)	985.7 (765.7 to 1300)	1537.9 (1190.9 to 1908.3)	891.8 (751.3 to 1025.7)	703.8 (584.6 to 833.9)	1086.7 (879.9 to 1287.4)	-27.8 (-40.5 to -11.8)	-28.6 (-45 to -5.3)	-29.3 (-45.9 to -9.7)
	YLLs	892.1 (692.8 to 1113.1)	656 (457.8 to 952.9)	1176.6 (860.3 to 1554.1)	548.9 (432.8 to 644.8)	378.2 (280.5 to 479.8)	727.9 (524.4 to 895.4)	-38.5 (-52.7 to -17.6)	-42.4 (-60.5 to -8.9)	-38.1 (-56.9 to -13.2)
	YLDs	343.4 (260.8 to 433.4)	329.6 (251.2 to 417.5)	361.3 (270.7 to 461.8)	342.9 (266.8 to 427.7)	325.7 (254.8 to 401)	358.9 (273.2 to 450)	-0.1 (-6.3 to 6.7)	-1.2 (-8.4 to 7.2)	-0.7 (-7.8 to 7.2)

Province	Measure	Age-standardized rate (per 100,000)						% Change (1990 to 2019)		
		1990			2019					
		Both	Female	Male	Both	Female	Male	Both	Female	Male
Zanjan	Incidence	941 (811.3 to 1095.2)	917.2 (793.2 to 1070)	963.2 (818.9 to 1121.1)	917.3 (787.7 to 1076.6)	905.1 (779.9 to 1057.2)	927.2 (788.2 to 1098.6)	-2.5 (-6.5 to 1.9)	-1.3 (-6.1 to 4.3)	-3.7 (-9 to 1.5)
	Prevalence	5725.5 (5087 to 6466.2)	5566.4 (4947.6 to 6274.3)	5867.3 (5200.4 to 6714.7)	5101.1 (4512.8 to 5789.8)	4887.6 (4334.7 to 5553.7)	5314 (4681.8 to 6062.8)	-10.9 (-15.2 to -6.3)	-12.2 (-17.2 to -6.5)	-9.4 (-14.9 to -3.6)
	Deaths	41.3 (32.9 to 53.5)	34.7 (24 to 55)	49.2 (37.9 to 64.1)	29.3 (24.9 to 33.5)	21.7 (16.4 to 26.8)	37.9 (32 to 44.2)	-29 (-45.4 to -9.7)	-37.5 (-60.9 to -10.4)	-23.1 (-42.6 to 2.1)
	DALYs	1129.7 (946.8 to 1357.7)	994.3 (784.3 to 1336.8)	1262.3 (1017.3 to 1555.9)	834.9 (731.6 to 940.5)	698 (587.9 to 811.3)	983.3 (854.2 to 1121.2)	-26.1 (-38.2 to -13.2)	-29.8 (-45.9 to -11.8)	-22.1 (-38 to -3.3)
	YLLs	789.7 (635.2 to 1005.3)	652.8 (456.4 to 985)	925.8 (702.8 to 1218.1)	494.5 (421.3 to 564.9)	362.4 (270.3 to 460.3)	638.5 (537.6 to 746.8)	-37.4 (-51.1 to -20)	-44.5 (-62.3 to -19.6)	-31 (-49.2 to -6.7)
	YLDs	339.9 (259.1 to 432.4)	341.5 (263.6 to 432)	336.5 (251.8 to 434.2)	340.4 (264.5 to 419.4)	335.6 (259.9 to 414.2)	344.8 (263.6 to 428.1)	0.1 (-5.6 to 6.4)	-1.7 (-8 to 5.1)	2.5 (-5.1 to 11.1)

Data in parentheses are 95% Uncertainty Intervals (95% UIs); DALYs= Disability-Adjusted Life Years; YLLs= Years of Life Lost; YLDs= Years Lived with Disability
