# Supplementary material for: National, subnational and risk attributed burden of chronic respiratory diseases in Iran from 1990 to 2019
Source: Respir Res. 2023 Mar 11;24:74. doi: 10.1186/s12931-023-02353-1 (PMC10006557; doi:10.1186/s12931-023-02353-1)
Supplement: Supplementary file 7 — Additional file 7: Table S3. Attributed burden measures of CRDs to all risk factors for all ages number and ASR with percentage change by sex at national level, 1990 vs 2019. Data in parentheses are 95% Uncertainty Intervals (95% UIs); DALYs= Disability-Adjusted Life Years; YLLs= Years of Life Lost; YLDs= Years Lived with Disability [file 12931_2023_2353_MOESM7_ESM.pdf]

Risk factor	Measure	Age (metric)	Year						% Change (1990 to 2019)		
			1990			2019					
			Both	Female	Male	Both	Female	Male	Both	Female	Male
Ambient ozone pollution	Deaths	All ages (number)	497 (223 to 776)	190 (81 to 326)	307 (137 to 491)	1,794 (842 to 2,766)	689 (319 to 1,090)	1,105 (519 to 1,708)	261.3 (192.6 to 323.9)	262.6 (163.8 to 545.7)	260.5 (176.4 to 335.6)
		Age-standardized (rate per 100,000)	2.8 (1.3 to 4.5)	2.3 (1 to 4)	3.4 (1.5 to 5.4)	2.9 (1.4 to 4.5)	2.3 (1.1 to 3.7)	3.5 (1.6 to 5.4)	2.3 (-17.9 to 20.3)	1.1 (-26.5 to 72)	1.9 (-21.5 to 23.2)
	DALYs	All ages (number)	11,053 (5,014 to 17,277)	3,974 (1,732 to 6,637)	7,079 (3,172 to 11,335)	31,852 (15,156 to 48,998)	11,509 (5,373 to 18,039)	20,344 (9,507 to 31,223)	188.2 (132.6 to 236.7)	189.6 (116.4 to 441.2)	187.4 (116.3 to 246.2)
		Age-standardized (rate per 100,000)	47.6 (21.3 to 74.2)	36.8 (15.9 to 62.4)	58 (26.1 to 92.6)	46 (21.9 to 70.8)	34.3 (15.9 to 54.1)	57.6 (27 to 88.7)	-3.4 (-21.4 to 13.1)	-6.7 (-30.6 to 66)	-0.7 (-24.3 to 20.4)
	YLLs	All ages (number)	11,053 (5,014 to 17,277)	3,974 (1,732 to 6,637)	7,079 (3,172 to 11,335)	31,852 (15,156 to 48,998)	11,509 (5,373 to 18,039)	20,344 (9,507 to 31,223)	188.2 (132.6 to 236.7)	189.6 (116.4 to 441.2)	187.4 (116.3 to 246.2)
		Age-standardized (rate per 100,000)	47.6 (21.3 to 74.2)	36.8 (15.9 to 62.4)	58 (26.1 to 92.6)	46 (21.9 to 70.8)	34.3 (15.9 to 54.1)	57.6 (27 to 88.7)	-3.4 (-21.4 to 13.1)	-6.7 (-30.6 to 66)	-0.7 (-24.3 to 20.4)
	YLDs	All ages (number)	-	-	-	-	-	-	-	-	-
		Age-standardized (rate per 100,000)	-	-	-	-	-	-	-	-	-

Risk factor	Measure	Age (metric)	Year						% Change (1990 to 2019)		
			1990			2019					
			Both	Female	Male	Both	Female	Male	Both	Female	Male
Ambient particulate matter pollution	Deaths	All ages (number)	893 (659 to 1,181)	337 (218 to 497)	556 (405 to 749)	2,954 (2,152 to 3,784)	1,141 (785 to 1,484)	1,813 (1,337 to 2,319)	230.8 (164.5 to 293.2)	238.1 (136.6 to 495)	226.3 (144.5 to 293.9)
		Age-standardized (rate per 100,000)	5.1 (3.7 to 6.8)	4.1 (2.7 to 6.1)	6.2 (4.5 to 8.4)	4.8 (3.5 to 6.1)	3.8 (2.6 to 5)	5.7 (4.2 to 7.3)	-6.8 (-26.8 to 11.8)	-6.2 (-34.5 to 60.5)	-8.3 (-30.3 to 9.6)
	DALYs	All ages (number)	28,787 (21,748 to 36,704)	11,226 (8,049 to 15,112)	17,561 (13,201 to 22,798)	85,148 (63,783 to 107,389)	34,328 (25,151 to 44,505)	50,820 (37,937 to 64,388)	195.8 (151.2 to 235.8)	205.8 (145.6 to 325.8)	189.4 (133.5 to 235.4)
		Age-standardized (rate per 100,000)	116.9 (87.5 to 149.9)	95.5 (67.2 to 132)	137.6 (103.5 to 179.4)	117.9 (88.1 to 149.4)	96.7 (70.3 to 124.5)	139.4 (104.4 to 177)	0.9 (-14.5 to 15.3)	1.2 (-21.4 to 42.7)	1.3 (-18.4 to 17.9)
	YLLs	All ages (number)	19,808 (14,699 to 26,163)	7,013 (4,445 to 10,224)	12,794 (9,369 to 17,269)	52,471 (38,866 to 66,925)	19,086 (13,386 to 24,713)	33,386 (24,787 to 42,489)	164.9 (110.7 to 214.4)	172.1 (99.8 to 397.3)	160.9 (93.5 to 216.5)
		Age-standardized (rate per 100,000)	85.5 (63.2 to 112.7)	65.3 (42.3 to 95.5)	105.2 (77.1 to 141.2)	75.7 (55.5 to 96.6)	56.9 (39.8 to 73.5)	94.5 (70.2 to 120.6)	-11.5 (-29 to 5.1)	-12.9 (-36.7 to 53.6)	-10.2 (-32.3 to 8)
	YLDs	All ages (number)	8,979 (6,545 to 11,850)	4,212 (3,074 to 5,595)	4,767 (3,411 to 6,361)	32,676 (23,676 to 43,230)	15,243 (11,011 to 20,303)	17,434 (12,330 to 22,998)	263.9 (239.4 to 294.9)	261.9 (234.5 to 297.7)	265.7 (240.7 to 299.2)
		Age-standardized (rate per 100,000)	31.4 (22.8 to 41.4)	30.2 (22 to 40.1)	32.4 (23.2 to 43.2)	42.3 (30.5 to 55.9)	39.8 (28.5 to 52.8)	44.8 (31.9 to 59.5)	34.7 (26.2 to 45.8)	31.6 (22.4 to 43.8)	38.5 (29.6 to 49.6)

Risk factor	Measure	Age (metric)	Year						% Change (1990 to 2019)		
			1990			2019					
			Both	Female	Male	Both	Female	Male	Both	Female	Male
High body–mass index	Deaths	All ages (number)	694 (360 to 1,156)	363 (175 to 652)	331 (147 to 582)	1,071 (661 to 1,563)	545 (319 to 803)	526 (288 to 821)	54.3 (9.7 to 120.5)	50.2 (-10.6 to 125.9)	58.9 (19.3 to 145.9)
		Age-standardized (rate per 100,000)	3.3 (1.7 to 5.8)	3.6 (1.6 to 6.9)	3.1 (1.3 to 5.6)	1.6 (1 to 2.4)	1.7 (1 to 2.6)	1.6 (0.9 to 2.5)	-50.6 (-67.3 to -27.1)	-51.7 (-73.5 to -23)	-48.7 (-62.8 to -17.7)
	DALYs	All ages (number)	27,026 (15,166 to 42,581)	14,799 (8,179 to 23,743)	12,227 (6,304 to 20,697)	45,173 (28,239 to 65,667)	23,463 (14,357 to 34,457)	21,709 (12,615 to 33,250)	67.1 (33 to 117.9)	58.5 (18.9 to 110.4)	77.6 (39.2 to 155.5)
		Age-standardized (rate per 100,000)	89.3 (48.3 to 146.8)	99.6 (52.1 to 167)	79.3 (39 to 138.1)	57 (36.3 to 81.8)	59.8 (36.6 to 87.3)	54.4 (32.1 to 82.7)	-36.2 (-52.1 to -12)	-40 (-59 to -16.1)	-31.4 (-48.1 to 5.6)
	YLLs	All ages (number)	17,928 (9,804 to 28,580)	9,396 (4,869 to 15,625)	8,532 (4,038 to 14,652)	22,572 (14,194 to 32,396)	11,105 (6,549 to 16,192)	11,468 (6,555 to 17,870)	25.9 (-5.7 to 77.1)	18.2 (-23.5 to 70.5)	34.4 (1.6 to 110.6)
		Age-standardized (rate per 100,000)	66.4 (34.6 to 110.1)	71.7 (34.8 to 128.2)	61.2 (27.2 to 107.9)	30.7 (19.2 to 44.2)	30.7 (18.2 to 44.9)	30.8 (17.5 to 48)	-53.8 (-67.5 to -33.4)	-57.2 (-74.3 to -34.8)	-49.6 (-61.9 to -19)
	YLDs	All ages (number)	9,098 (4,625 to 15,498)	5,403 (2,774 to 9,082)	3,695 (1,752 to 6,610)	22,600 (12,562 to 36,157)	12,358 (6,814 to 20,640)	10,242 (5,426 to 17,415)	148.4 (117.2 to 208.4)	128.7 (99.9 to 179.1)	177.2 (133.4 to 275.8)
		Age-standardized (rate per 100,000)	22.8 (11.5 to 39.2)	27.9 (14.2 to 47.4)	18.1 (8.2 to 33.6)	26.3 (14.6 to 42.3)	29.1 (16.4 to 47.7)	23.6 (12.7 to 40.1)	15.1 (-2.9 to 45.1)	4.2 (-10.3 to 28.7)	30.1 (3.8 to 83)

Risk factor	Measure	Age (metric)	Year						% Change (1990 to 2019)		
			1990			2019					
			Both	Female	Male	Both	Female	Male	Both	Female	Male
High temperature	Deaths	All ages (number)	5 (-46 to 47)	2 (-18 to 19)	3 (-28 to 29)	74 (-26 to 175)	29 (-10 to 70)	46 (-17 to 108)	1469.1 (-4185 to 3777)	1359.2 (-4627.3 to 4362.3)	1547.3 (-4090.9 to 3057.6)
		Age-standardized (rate per 100,000)	0 (-0.2 to 0.3)	0 (-0.2 to 0.2)	0 (-0.3 to 0.3)	0.1 (0 to 0.3)	0.1 (0 to 0.2)	0.1 (-0.1 to 0.3)	303 (-1347.2 to 942)	273.9 (-1431.7 to 1241.8)	321.1 (-1203.4 to 1125.2)
	DALYs	All ages (number)	104 (-1,186 to 1,159)	39 (-464 to 446)	64 (-748 to 739)	1,372 (-588 to 3,308)	503 (-215 to 1,240)	870 (-370 to 2,087)	1222.5 (-2488.7 to 2818.1)	1173.9 (-3315.4 to 2446.5)	1252.4 (-2964.9 to 2558.5)
		Age-standardized (rate per 100,000)	0.5 (-4.4 to 4.5)	0.4 (-3.4 to 3.7)	0.5 (-5.4 to 5.5)	2 (-0.8 to 4.7)	1.5 (-0.6 to 3.7)	2.4 (-0.9 to 5.9)	329.8 (-1095.1 to 1019.9)	290.7 (-1158.8 to 1038.8)	362.4 (-1124.1 to 820.4)
	YLLs	All ages (number)	104 (-1,186 to 1,159)	39 (-464 to 446)	64 (-748 to 739)	1,372 (-588 to 3,308)	503 (-215 to 1,240)	870 (-370 to 2,087)	1222.5 (-2488.7 to 2818.1)	1173.9 (-3315.4 to 2446.5)	1252.4 (-2964.9 to 2558.5)
		Age-standardized (rate per 100,000)	0.5 (-4.4 to 4.5)	0.4 (-3.4 to 3.7)	0.5 (-5.4 to 5.5)	2 (-0.8 to 4.7)	1.5 (-0.6 to 3.7)	2.4 (-0.9 to 5.9)	329.8 (-1095.1 to 1019.9)	290.7 (-1158.8 to 1038.8)	362.4 (-1124.1 to 820.4)
	YLDs	All ages (number)	-	-	-	-	-	-	-	-	-
		Age-standardized (rate per 100,000)	-	-	-	-	-	-	-	-	-

Risk factor	Measure	Age (metric)	Year						% Change (1990 to 2019)		
			1990			2019					
			Both	Female	Male	Both	Female	Male	Both	Female	Male
Household air pollution from solid fuels	Deaths	All ages (number)	204 (86 to 393)	93 (38 to 183)	111 (45 to 220)	7 (2 to 16)	3 (1 to 8)	4 (1 to 8)	-96.6 (-98.7 to -91.4)	-96.3 (-98.7 to -90.4)	-96.8 (-98.8 to -91.7)
		Age-standardized (rate per 100,000)	1.2 (0.5 to 2.2)	1.1 (0.4 to 2.2)	1.2 (0.5 to 2.3)	0 (0 to 0)	0 (0 to 0)	0 (0 to 0)	-99 (-99.6 to -97.6)	-99 (-99.6 to -97.3)	-99.1 (-99.7 to -97.6)
	DALYs	All ages (number)	6,598 (2,812 to 12,490)	3,136 (1,336 to 5,968)	3,462 (1,376 to 6,732)	206 (74 to 476)	106 (39 to 242)	100 (34 to 226)	-96.9 (-98.8 to -92.3)	-96.6 (-98.7 to -91.7)	-97.1 (-98.9 to -92.6)
		Age-standardized (rate per 100,000)	26.6 (11.3 to 51.1)	26.2 (11 to 50.6)	26.8 (10.8 to 52.1)	0.3 (0.1 to 0.6)	0.3 (0.1 to 0.7)	0.3 (0.1 to 0.6)	-99 (-99.6 to -97.4)	-98.9 (-99.6 to -97.2)	-99 (-99.6 to -97.5)
	YLLs	All ages (number)	4,590 (1,911 to 8,819)	2,002 (830 to 3,918)	2,588 (1,036 to 5,079)	132 (45 to 309)	62 (22 to 150)	69 (23 to 158)	-97.1 (-98.9 to -92.9)	-96.9 (-98.8 to -92)	-97.3 (-99 to -93)
		Age-standardized (rate per 100,000)	19.6 (8.3 to 38)	18.1 (7.5 to 35.3)	21 (8.4 to 41.6)	0.2 (0.1 to 0.4)	0.2 (0.1 to 0.4)	0.2 (0.1 to 0.4)	-99.1 (-99.6 to -97.7)	-99 (-99.6 to -97.4)	-99.1 (-99.7 to -97.6)
	YLDs	All ages (number)	2,007 (844 to 3,868)	1,134 (494 to 2,120)	874 (350 to 1,815)	74 (28 to 168)	43 (17 to 101)	31 (11 to 72)	-96.3 (-98.6 to -90.9)	-96.2 (-98.5 to -90.6)	-96.5 (-98.6 to -91.2)
		Age-standardized (rate per 100,000)	7 (3 to 13.5)	8.1 (3.5 to 14.9)	5.9 (2.4 to 12.2)	0.1 (0 to 0.2)	0.1 (0 to 0.3)	0.1 (0 to 0.2)	-98.7 (-99.5 to -96.7)	-98.6 (-99.5 to -96.6)	-98.7 (-99.5 to -96.7)

Risk factor	Measure	Age (metric)	Year						% Change (1990 to 2019)		
			1990			2019					
			Both	Female	Male	Both	Female	Male	Both	Female	Male
Low temperature	Deaths	All ages (number)	654 (518 to 863)	255 (172 to 376)	400 (310 to 534)	1,973 (1,598 to 2,352)	764 (584 to 947)	1,209 (969 to 1,439)	201.7 (143.3 to 251.6)	200.1 (114.8 to 434)	202.6 (128.3 to 261.6)
		Age-standardized (rate per 100,000)	3.6 (2.8 to 4.8)	3 (2 to 4.3)	4.3 (3.3 to 5.8)	3.2 (2.6 to 3.8)	2.6 (2 to 3.2)	3.8 (3.1 to 4.5)	-11.7 (-29.9 to 4.6)	-12.6 (-38.7 to 48.3)	-12 (-32.4 to 5.2)
	DALYs	All ages (number)	16,219 (12,910 to 20,755)	6,124 (3,982 to 8,751)	10,094 (7,843 to 13,603)	35,460 (28,705 to 42,102)	12,979 (10,014 to 16,303)	22,481 (17,951 to 26,600)	118.6 (74.8 to 153.1)	111.9 (58.7 to 314.8)	122.7 (65.5 to 168.3)
		Age-standardized (rate per 100,000)	62.5 (49.4 to 82.3)	49.1 (33.4 to 73.3)	75.4 (58.5 to 100.6)	51.4 (41.8 to 60.9)	38.9 (29.6 to 48.8)	64 (51.4 to 75.7)	-17.7 (-33.1 to -4.1)	-20.8 (-41.7 to 43.2)	-15.2 (-35.9 to 1.2)
	YLLs	All ages (number)	16,219 (12,910 to 20,755)	6,124 (3,982 to 8,751)	10,094 (7,843 to 13,603)	35,460 (28,705 to 42,102)	12,979 (10,014 to 16,303)	22,481 (17,951 to 26,600)	118.6 (74.8 to 153.1)	111.9 (58.7 to 314.8)	122.7 (65.5 to 168.3)
		Age-standardized (rate per 100,000)	62.5 (49.4 to 82.3)	49.1 (33.4 to 73.3)	75.4 (58.5 to 100.6)	51.4 (41.8 to 60.9)	38.9 (29.6 to 48.8)	64 (51.4 to 75.7)	-17.7 (-33.1 to -4.1)	-20.8 (-41.7 to 43.2)	-15.2 (-35.9 to 1.2)
	YLDs	All ages (number)	-	-	-	-	-	-	-	-	-
		Age-standardized (rate per 100,000)	-	-	-	-	-	-	-	-	-

Risk factor	Measure	Age (metric)	Year						% Change (1990 to 2019)		
			1990			2019					
			Both	Female	Male	Both	Female	Male	Both	Female	Male
Occupational asthmagens	Deaths	All ages (number)	194 (149 to 245)	17 (11 to 25)	177 (133 to 226)	134 (112 to 157)	15 (11 to 20)	119 (98 to 141)	-31.1 (-47 to -5.3)	-12.9 (-48.3 to 44.2)	-32.9 (-49.5 to -5.8)
		Age-standardized (rate per 100,000)	0.7 (0.5 to 0.9)	0.1 (0.1 to 0.2)	1.2 (0.9 to 1.5)	0.2 (0.1 to 0.2)	0 (0 to 0)	0.3 (0.2 to 0.4)	-75.1 (-81.4 to -65.2)	-70.6 (-83.4 to -47.5)	-74.7 (-81.4 to -63.8)
	DALYs	All ages (number)	9,676 (7,616 to 12,076)	1,062 (749 to 1,502)	8,614 (6,722 to 10,801)	10,217 (7,976 to 13,421)	1,570 (1,119 to 2,167)	8,648 (6,702 to 11,254)	5.6 (-13.8 to 30.7)	47.8 (6.2 to 108.2)	0.4 (-19 to 26)
		Age-standardized (rate per 100,000)	27.4 (21.1 to 34.2)	5.9 (4.2 to 8.3)	47.1 (36.3 to 59.6)	11.1 (8.8 to 14.3)	3.3 (2.4 to 4.5)	18.7 (14.7 to 24.2)	-59.6 (-67.6 to -49)	-44.3 (-60.7 to -20.6)	-60.3 (-68.9 to -49.2)
	YLLs	All ages (number)	6,156 (4,820 to 7,599)	599 (404 to 842)	5,557 (4,291 to 6,979)	4,505 (3,784 to 5,279)	553 (417 to 733)	3,952 (3,189 to 4,680)	-26.8 (-42.6 to 0.5)	-7.6 (-42 to 48.1)	-28.9 (-45.5 to -0.2)
		Age-standardized (rate per 100,000)	18.9 (14.7 to 23.6)	3.7 (2.4 to 5.2)	32.9 (24.9 to 41.6)	5.1 (4.3 to 6)	1.2 (0.9 to 1.6)	9.1 (7.4 to 10.8)	-72.9 (-79 to -62.5)	-66.9 (-80 to -45.4)	-72.4 (-79 to -61.2)
	YLDs	All ages (number)	3,520 (2,182 to 5,351)	463 (272 to 727)	3,056 (1,888 to 4,700)	5,712 (3,613 to 8,588)	1,016 (615 to 1,563)	4,696 (2,981 to 7,108)	62.3 (38.9 to 85.8)	119.3 (67 to 195.3)	53.6 (29.8 to 78.2)
		Age-standardized (rate per 100,000)	8.4 (5.2 to 12.7)	2.3 (1.3 to 3.5)	14.2 (8.8 to 21.6)	5.9 (3.8 to 8.8)	2.1 (1.3 to 3.2)	9.6 (6.1 to 14.5)	-29.7 (-39.1 to -20)	-7.5 (-29.3 to 22.6)	-32.1 (-41.8 to -21.5)

Risk factor	Measure	Age (metric)	Year						% Change (1990 to 2019)		
			1990			2019					
			Both	Female	Male	Both	Female	Male	Both	Female	Male
Occupational exposure to asbestos	Deaths	All ages (number)	2 (2 to 3)	1 (0 to 1)	2 (1 to 3)	13 (10 to 21)	3 (1 to 5)	10 (8 to 17)	495.6 (304.3 to 827.6)	527.1 (237.5 to 1072.7)	485.3 (242 to 889.9)
		Age-standardized (rate per 100,000)	0 (0 to 0)	0 (0 to 0)	0 (0 to 0)	0 (0 to 0)	0 (0 to 0)	0 (0 to 0.1)	101.7 (34.8 to 211.8)	78.6 (-5.7 to 242.3)	114.4 (19.6 to 260.5)
	DALYs	All ages (number)	136 (100 to 184)	83 (55 to 120)	53 (35 to 78)	1,067 (759 to 1,463)	851 (554 to 1,249)	215 (173 to 314)	684.9 (511.9 to 856.2)	931.5 (747.1 to 1103)	303.6 (147.8 to 537)
		Age-standardized (rate per 100,000)	0.4 (0.3 to 0.5)	0.5 (0.3 to 0.7)	0.3 (0.2 to 0.5)	1.3 (0.9 to 1.7)	2 (1.3 to 2.9)	0.6 (0.5 to 0.9)	219.2 (146.8 to 293.3)	323.8 (253.3 to 390)	76.5 (7.2 to 175.2)
	YLLs	All ages (number)	64 (44 to 93)	14 (7 to 26)	50 (32 to 75)	288 (231 to 408)	76 (25 to 118)	212 (169 to 309)	350.8 (201.8 to 585.2)	434.5 (169.3 to 947.5)	326.6 (154.4 to 596.2)
		Age-standardized (rate per 100,000)	0.2 (0.1 to 0.3)	0.1 (0 to 0.2)	0.3 (0.2 to 0.5)	0.4 (0.3 to 0.6)	0.2 (0.1 to 0.3)	0.6 (0.4 to 0.9)	84.6 (24.9 to 176.8)	96.3 (-0.5 to 263.8)	86 (10.3 to 198.1)
	YLDs	All ages (number)	72 (45 to 110)	68 (43 to 104)	4 (2 to 6)	779 (489 to 1,175)	775 (486 to 1,170)	4 (2 to 7)	981.6 (826 to 1145.4)	1035.7 (871.8 to 1210.4)	0.5 (-35.5 to 34.4)
		Age-standardized (rate per 100,000)	0.2 (0.1 to 0.3)	0.4 (0.2 to 0.5)	0 (0 to 0)	0.9 (0.6 to 1.3)	1.8 (1.1 to 2.7)	0 (0 to 0)	366.5 (313 to 421)	389.9 (334.4 to 446.8)	-58.5 (-71.4 to -45.4)

Risk factor	Measure	Age (metric)	Year						% Change (1990 to 2019)		
			1990			2019					
			Both	Female	Male	Both	Female	Male	Both	Female	Male
Occupational exposure to silica	Deaths	All ages (number)	4 (2 to 5)	0 (0 to 1)	3 (2 to 5)	15 (6 to 20)	3 (1 to 4)	13 (4 to 17)	326 (52.6 to 754.8)	656.2 (308.3 to 1207)	287.5 (0.1 to 752.5)
		Age-standardized (rate per 100,000)	0 (0 to 0)	0 (0 to 0)	0 (0 to 0)	0 (0 to 0)	0 (0 to 0)	0 (0 to 0.1)	49.7 (-46.7 to 196.2)	140.7 (20.8 to 314.1)	36.2 (-63.9 to 200.7)
	DALYs	All ages (number)	107 (68 to 150)	13 (8 to 21)	94 (56 to 137)	396 (187 to 497)	68 (35 to 104)	327 (117 to 427)	268.8 (58.4 to 586.9)	430.8 (231.3 to 762.8)	246.8 (15.4 to 603.8)
		Age-standardized (rate per 100,000)	0.4 (0.2 to 0.5)	0.1 (0.1 to 0.1)	0.6 (0.4 to 0.9)	0.5 (0.3 to 0.7)	0.2 (0.1 to 0.3)	0.9 (0.3 to 1.2)	45.7 (-36.7 to 165.1)	118.8 (33.5 to 241.6)	39.3 (-52.8 to 178.7)
	YLLs	All ages (number)	99 (60 to 142)	11 (7 to 20)	87 (49 to 129)	354 (150 to 453)	63 (30 to 99)	290 (82 to 388)	257.5 (36.9 to 618.2)	450.4 (232.6 to 841.8)	232.2 (-14.6 to 630.9)
		Age-standardized (rate per 100,000)	0.3 (0.2 to 0.5)	0.1 (0 to 0.1)	0.6 (0.3 to 0.8)	0.5 (0.2 to 0.6)	0.2 (0.1 to 0.3)	0.8 (0.2 to 1)	41.4 (-46.7 to 178.8)	130.6 (30.6 to 275.5)	33.6 (-65.3 to 194.1)
	YLDs	All ages (number)	8 (5 to 13)	1 (1 to 2)	7 (4 to 11)	42 (26 to 64)	5 (3 to 7)	37 (23 to 58)	403.4 (298.8 to 560.5)	259.4 (204.1 to 382)	430.5 (313.9 to 604.3)
		Age-standardized (rate per 100,000)	0 (0 to 0.1)	0 (0 to 0)	0.1 (0 to 0.1)	0.1 (0 to 0.1)	0 (0 to 0)	0.1 (0.1 to 0.2)	88.2 (52.5 to 143.7)	34.4 (17 to 71.5)	96.7 (57.1 to 160)

Risk factor	Measure	Age (metric)	Year						% Change (1990 to 2019)		
			1990			2019					
			Both	Female	Male	Both	Female	Male	Both	Female	Male
Occupational particulate matter, gases, and fumes	Deaths	All ages (number)	467 (357 to 609)	42 (26 to 66)	425 (317 to 562)	1,370 (1,054 to 1,717)	138 (94 to 191)	1,232 (915 to 1,582)	193.6 (119 to 263.5)	226.7 (129.2 to 451.8)	190.3 (107.7 to 267)
		Age-standardized (rate per 100,000)	2.4 (1.8 to 3.2)	0.5 (0.3 to 0.8)	4.5 (3.2 to 6.1)	2.2 (1.6 to 2.8)	0.5 (0.3 to 0.6)	3.9 (2.8 to 5)	-9.7 (-29.9 to 7.3)	-8.7 (-36.9 to 51.8)	-14.1 (-34.6 to 3.7)
	DALYs	All ages (number)	14,656 (11,700 to 18,213)	1,364 (952 to 1,943)	13,292 (10,317 to 16,809)	38,763 (31,681 to 45,942)	4,264 (3,295 to 5,319)	34,499 (27,723 to 41,403)	164.5 (112.8 to 208)	212.6 (141.2 to 331)	159.5 (102.5 to 207.8)
		Age-standardized (rate per 100,000)	57.4 (45.3 to 72.1)	11.7 (8 to 17)	102.6 (79.2 to 130.1)	54 (44 to 64.7)	11.9 (9 to 15.2)	96.1 (76.2 to 117.1)	-5.9 (-22.8 to 7.8)	1.7 (-21 to 43.3)	-6.4 (-24.7 to 8.4)
	YLLs	All ages (number)	10,739 (8,300 to 13,921)	893 (556 to 1,323)	9,846 (7,438 to 13,155)	25,599 (20,795 to 30,593)	2,470 (1,817 to 3,180)	23,128 (18,381 to 28,058)	138.4 (79.2 to 191.1)	176.7 (101.8 to 399.3)	134.9 (70.3 to 190.8)
		Age-standardized (rate per 100,000)	43.7 (34 to 56.3)	8.2 (5.1 to 12.6)	79.1 (59.6 to 104.5)	36.7 (29.5 to 44.2)	7.2 (5.2 to 9.6)	66.2 (52.3 to 81.1)	-16 (-35 to -0.2)	-11.7 (-36.6 to 54.8)	-16.3 (-37.2 to 0.9)
	YLDs	All ages (number)	3,917 (2,944 to 4,923)	471 (330 to 651)	3,446 (2,518 to 4,399)	13,164 (10,112 to 16,246)	1,794 (1,312 to 2,291)	11,370 (8,577 to 14,309)	236 (210.1 to 268)	280.6 (223.7 to 352)	229.9 (201 to 264.3)
		Age-standardized (rate per 100,000)	13.7 (10.3 to 16.9)	3.5 (2.5 to 4.8)	23.5 (17.3 to 29.9)	17.3 (13.2 to 21.7)	4.7 (3.4 to 6.1)	29.9 (22.3 to 38.4)	26.5 (18.8 to 35.4)	33.2 (15.7 to 55.4)	27.1 (19.7 to 35.5)

Risk factor	Measure	Age (metric)	Year						% Change (1990 to 2019)		
			1990			2019					
			Both	Female	Male	Both	Female	Male	Both	Female	Male
Secondhand smoke	Deaths	All ages (number)	388 (195 to 600)	165 (77 to 266)	223 (107 to 350)	1,171 (603 to 1,773)	503 (253 to 774)	668 (344 to 1,012)	202 (138 to 266.4)	205 (117.3 to 436.5)	199.8 (121.6 to 276.7)
		Age-standardized (rate per 100,000)	2.2 (1.1 to 3.3)	1.9 (0.9 to 3.1)	2.5 (1.2 to 3.9)	1.9 (1 to 2.8)	1.7 (0.8 to 2.5)	2.1 (1.1 to 3.2)	-13.1 (-31.8 to 5.1)	-11.6 (-37.7 to 51.6)	-15 (-37.3 to 7.5)
	DALYs	All ages (number)	13,109 (6,763 to 19,808)	6,234 (3,114 to 9,533)	6,876 (3,481 to 10,747)	35,733 (18,543 to 53,775)	17,399 (8,936 to 26,058)	18,335 (9,586 to 28,181)	172.6 (134.3 to 212.4)	179.1 (131.4 to 284.3)	166.7 (109.5 to 222.8)
		Age-standardized (rate per 100,000)	51.6 (26.2 to 78.3)	49 (23.9 to 76.3)	54.1 (27.6 to 83.8)	48.8 (25.4 to 73.4)	46.8 (24.3 to 70)	51 (26.6 to 78.3)	-5.3 (-20 to 9)	-4.5 (-23.6 to 31.1)	-5.8 (-26.5 to 14.3)
	YLLs	All ages (number)	8,817 (4,398 to 13,694)	3,751 (1,739 to 5,947)	5,066 (2,457 to 8,032)	21,314 (11,060 to 31,872)	9,164 (4,675 to 13,987)	12,149 (6,167 to 18,269)	141.7 (92.9 to 192.3)	144.3 (82.2 to 344.2)	139.8 (74.6 to 202.4)
		Age-standardized (rate per 100,000)	37 (18.7 to 57.1)	32.3 (15 to 51.8)	41.7 (20 to 65.2)	30.5 (15.9 to 45.9)	26.3 (13.4 to 40.2)	34.8 (17.8 to 52.3)	-17.7 (-34.7 to -0.6)	-18.7 (-40.4 to 46)	-16.6 (-38.4 to 4.6)
	YLDs	All ages (number)	4,292 (2,209 to 6,645)	2,483 (1,287 to 3,856)	1,809 (904 to 2,890)	14,420 (7,434 to 22,153)	8,234 (4,160 to 12,591)	6,185 (3,129 to 9,535)	236 (208.7 to 264.7)	231.7 (204.1 to 261)	241.8 (196.4 to 292.8)
		Age-standardized (rate per 100,000)	14.5 (7.4 to 22.5)	16.7 (8.6 to 25.9)	12.4 (6.2 to 19.7)	18.3 (9.2 to 28.2)	20.5 (10.4 to 31.4)	16.2 (8 to 25.2)	26.2 (16.7 to 36.5)	22.8 (13.1 to 33.2)	30.6 (14.5 to 48.2)

Risk factor	Measure	Age (metric)	Year						% Change (1990 to 2019)		
			1990			2019					
			Both	Female	Male	Both	Female	Male	Both	Female	Male
Smoking	Deaths	All ages (number)	2,366 (1,991 to 2,923)	315 (207 to 447)	2,052 (1,716 to 2,629)	5,300 (4,650 to 5,936)	645 (475 to 827)	4,654 (4,058 to 5,251)	124 (79.5 to 163.1)	105.1 (42.2 to 237.8)	126.9 (74 to 172.6)
		Age-standardized (rate per 100,000)	11.6 (9.7 to 14.3)	3.1 (2 to 4.5)	20.8 (17.2 to 26.1)	8.3 (7.3 to 9.4)	2 (1.5 to 2.6)	14.5 (12.5 to 16.4)	-28.1 (-42.7 to -16.4)	-34.5 (-55.2 to 8.7)	-30.5 (-46.6 to -17.5)
	DALYs	All ages (number)	73,912 (62,396 to 88,555)	10,496 (7,559 to 14,117)	63,415 (53,168 to 77,585)	155,978 (136,634 to 173,945)	22,110 (17,450 to 27,581)	133,868 (117,002 to 149,297)	111 (75.1 to 140.2)	110.6 (59 to 199.8)	111.1 (71.2 to 144.6)
		Age-standardized (rate per 100,000)	285.2 (242.9 to 341.7)	84 (59.6 to 112.7)	484 (409.2 to 591.9)	216 (189.9 to 240.8)	61.4 (48 to 76.9)	369.8 (324.9 to 411.6)	-24.3 (-37.1 to -14.3)	-26.9 (-44.9 to 4.3)	-23.6 (-37.7 to -11.9)
	YLLs	All ages (number)	55,141 (46,564 to 68,508)	7,158 (4,744 to 9,897)	47,983 (40,037 to 61,265)	101,466 (89,211 to 112,257)	12,718 (9,647 to 16,187)	88,748 (77,018 to 98,623)	84 (45.8 to 115.1)	77.7 (27.7 to 184.4)	85 (40.3 to 122.7)
		Age-standardized (rate per 100,000)	219.4 (183.7 to 270.2)	59.5 (38.8 to 84.1)	378 (314 to 481.6)	144.7 (128.4 to 160.7)	36.7 (27.5 to 46.7)	252.4 (220.3 to 281.1)	-34 (-47.4 to -23.2)	-38.4 (-56.2 to 1.4)	-33.2 (-48.9 to -20.5)
	YLDs	All ages (number)	18,771 (14,228 to 23,199)	3,339 (2,319 to 4,474)	15,432 (11,633 to 19,024)	54,512 (42,575 to 65,572)	9,392 (7,083 to 11,865)	45,120 (35,286 to 54,837)	190.4 (165.7 to 218.3)	181.3 (120.1 to 262.4)	192.4 (169.3 to 220.1)
		Age-standardized (rate per 100,000)	65.9 (50.5 to 81.1)	24.5 (17.2 to 32.8)	106 (81 to 130.4)	71.3 (56.3 to 85.2)	24.8 (18.7 to 31.4)	117.4 (92.1 to 142)	8.2 (-0.6 to 18.4)	1.2 (-20.1 to 30.3)	10.7 (2.3 to 20.4)

Data in parentheses are 95% Uncertainty Intervals (95% UIs)

DALYs= Disability-Adjusted Life Years; YLLs= Years of Life Lost; YLDs= Years Lived with Disability
